# Multi‐Physically Programmable Tubular Origami Metamaterials: Exploitable Nexus of Geometry, Folding Mechanics and Stimuli‐Responsive Physics

**DOI:** 10.1002/advs.202505089

**Published:** 2025-08-25

**Authors:** A. Sharma, S. Naskar, T. Mukhopadhyay

**Affiliations:** ^1^ School of Engineering University of Southampton Southampton SO16 7QF UK

**Keywords:** active multi‐physical mechanics, kirigami metamaterials, origami metamaterials, programmable matter, tubular origami

## Abstract

Metamaterials and metastructures developed based on tubular origami‐inspired structural forms can leverage the convolution of geometry, crease mechanics and stimuli‐responsive physics to provide unique mechanical and functional properties, including geometric efficiency and compactness, deployability and reconfigurability, structural integration ability in complex shapes, stiffness and strength modulation, constitutive programming and deformation mode coupling, high specific energy absorption, multi‐stability, and programmable dynamic behavior, leading to diverse applications in the field of mechanical, robotics, space, electronic devices and communication, biomedical, and architecture. With stupendous advancement over the last decade in computational and manufacturing capabilities to realize complex crease architectures along with on‐demand programmability through coupling folding‐driven mechanics with stimuli‐responsive physics of electrical or magnetic fields, temperature, light, controlled chemical reactions, and pneumatic actuation, the field of origami‐inspired mechanical metamaterials has been attracting wide attention due to immense potential of achieving unprecedented multi‐physical and multi‐functional attributes that are typically not attainable in naturally‐occurring materials or traditional structures. This article endeavours to review the developments reported in relevant literature concerning mechanical and multi‐physical property modulation of tubular origami metamaterials, highlighting the broad‐spectrum potential in innovative applications across the length scales along with critically analysing the emerging trends, challenges and potential future research landscape.

## Graphical abstract

This article presents an insightful review on the exploitable nexus of geometry, folding mechanics and stimuli‐responsive physics for achieving active programmability in tubular origami metamaterials and metastructures, highlighting the broad‐spectrum potential in innovative applications across the length scales, along with critically analysing the emerging trends, challenges and potential future research landscape.

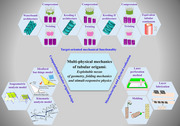

## 1 Introduction

***Metamaterials and Their Structural Classes*** Over the past decade, mechanical metamaterials have surged in popularity due to their innovative approach in incorporating hierarchical bottom‐up lower‐length scale architectures with intricate micro‐ and nano‐scale designs to achieve unprecedented mechanical and multi‐physical attributes (at a relatively higher length scale) which are not available in naturally‐occurring materials. The effective mechanical attributes in metamaterials are often driven by the geometry of unit cells along with the intrinsic material properties, leading to the possibility of modulating the effective mechanical behavior as a function of lower‐length scale architectures maintaining invariance of the base intrinsic material. The word metamaterial is derived from the Greek word “meta” i.e. “beyond”, representing an innovative class of materials engineered to exhibit extraordinary properties beyond the limits of conventional engineering materials. These architected materials are artificially manufactured using conventional materials (referred to as intrinsic materials), often organized in repetitive patterns at scales smaller than the wavelengths they influence for a dynamic system. In the case of evaluating static effective properties, it is ensured that there exists a significant difference in the length scales of unit cell geometry and the higher length scale at which the effective properties are defined, resulting in a converged number of unit cells. In case of the absence of an adequate number of unit cells such that homogenised properties cannot be evaluated, the term ‘metastructure’ is defined in the literature (note that even metastructures possess exotic properties attributed to the designed geometric architecture). In general, metamaterials derive their unique capabilities not only from their constituent materials unlike conventional materials, but from the deliberate design of their lower‐scale architectures as well through precise shaping, sizing, orientation, and arrangement.^[^ ^1^, ^2^, ^3^, ^4^, ^5^, ^6^, ^7^ ^]^ This leads to the manipulation of static and dynamic properties exceeding the capabilities of traditional materials, offering a wide range of possibilities for various engineering applications.^[^ ^8^, ^9^, ^10^, ^11^, ^12^, ^13^, ^14^ ^]^ Two primary classes of metamaterial architectures are typically conceptualized based on lattice and origami base patterns.^[^ ^12^, ^15^ ^]^ The central theme of this paper is origami‐inspired mechanical metamaterials wherein we will focus on tubular origami architectures due to their inherent advantages as discussed later in this section.

**Figure 1:**
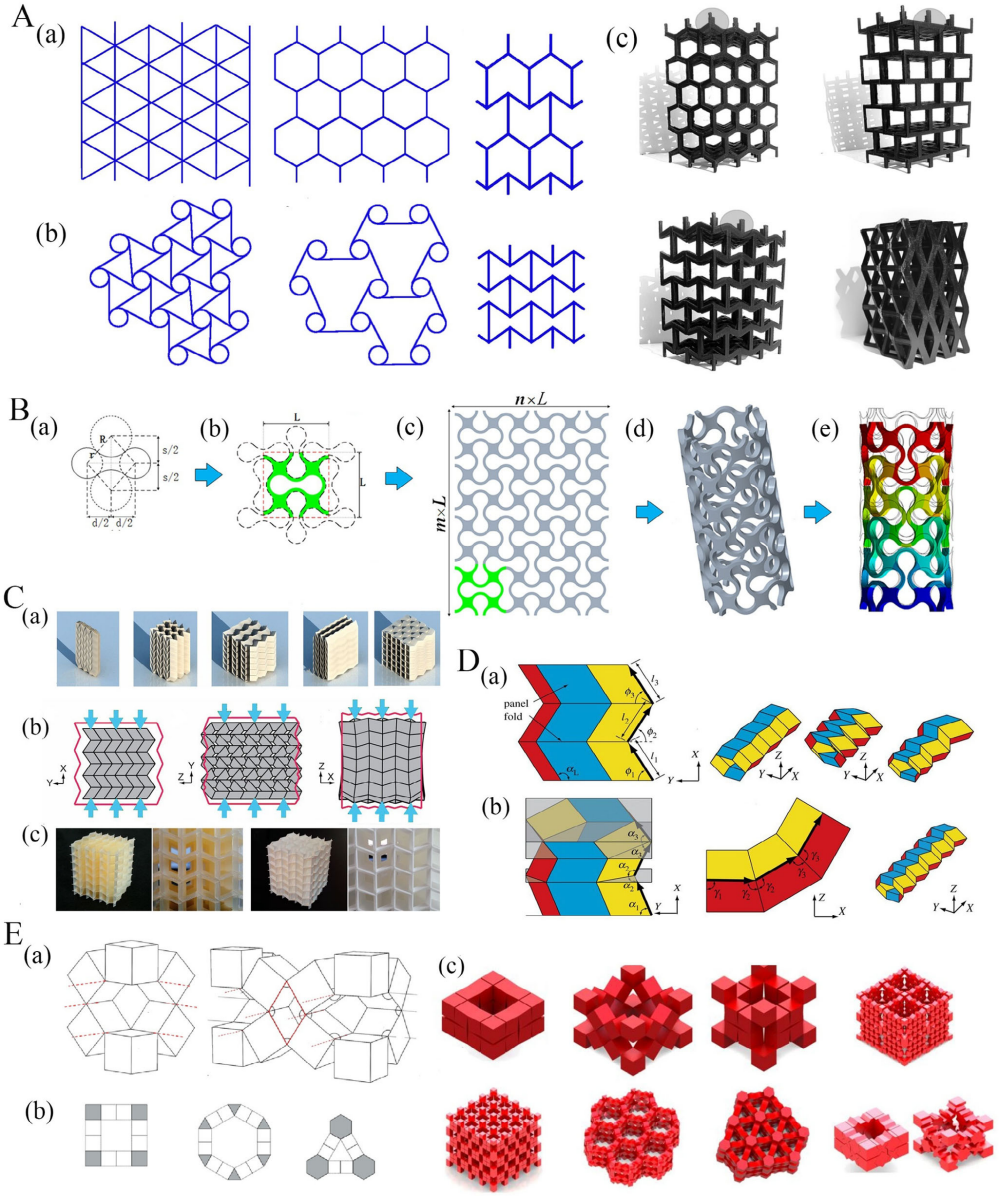
Interplay of geometry, material and mechanics for developing mechanical metamaterials and their emerging classes. A) 2D and 3D lattice based metamaterial architectures including positive, negative and zero Poisson's ratios (Reproduced with permission.^[^ ^16^ ^]^ Copyright 2018, Elsevier).^[^ ^12^, ^16^, ^17^, ^18^ ^]^ B) Lattice‐based tubular metamaterials showing the (a, b) unit cell, (c) planar auxetic lattice sheet obtained by tessellating the unit cell and (d) formation of tubular structure along with its deformation behavior (Reproduced with permission.^[^ ^19^ ^]^ Copyright 2021, Elsevier). C) Cellular origami based tubular metamaterials (a) Kinematic folding sequence of assembly (b) Compressive stiffness of assembly with initial (red line) and deformed geometry (c) Cellular metamaterial prototype constructed from additive manufacturing with resin for soft metamaterial and polyamide for hard metamaterials (Reproduced with permission.^[^ ^20^ ^]^ Copyright 2015, National Academy of Science). D) Rigid and flat foldable polygonal origami tubes showing the unit cell and its 3D representations (Reproduced with permission.^[^ ^21^ ^]^ Copyright 2016, The Royal Society). E) Sarrus modular origami metamaterials (a) Cubic unit cell and its interconnection to form Sarrus mechanism (b) Corner cube unit cell variation to obtain different tilting patterns (c) Large‐scale shape morphing (Reproduced under the terms of the CC‐BY 4.0 license.^[^ ^22^ ^]^ Copyright 2020, arXiv).

Out of the different classes of metamaterials, lattice‐based materials typically consist of cellular architectures where unit cells are organized periodically in one, two or 3D space (refer to **Figure** **1**A) to meet application‐specific (multi‐)functional requirements. These lattice‐based metamaterial architectures are widely utilized in weight‐critical applications such as aircraft, automotive structures, armours, and rotor blades due to their ability to attain high specific strength, stiffness and energy absorption capability.^[^ ^23^, ^24^ ^]^ The lattice metamaterials may also possess graded, quasi‐periodic, or non‐periodic microstructures.^[^ ^11^, ^25^, ^26^, ^27^, ^28^, ^29^ ^]^ Covering effective static, quasi‐static and dynamic mechanical properties, passive (the effective mechanical properties cannot be actively modulated once the lattice is manufactured^[^ ^30^, ^31^, ^32^, ^33^, ^34^, ^35^, ^36^, ^37^, ^38^ ^]^) and active lattice (the effective mechanical properties can be actively changed as a function of external stimuli like electric or magnetic field, air pressure etc.^[^ ^39^, ^40^, ^41^, ^42^, ^43^, ^44^ ^]^) architectures have been proposed in the literature. Considering the focus of this article to be tubular metamaterial architectures, it may be noted that lattice‐based tubular metamaterials have attracted wide attention where inspiration is often drawn from natural tubular structures like bamboo and oesophageal pipes. The lattice‐based tubular metamaterials are constructed from different two‐dimensional (2D) and three‐dimensional (3D) periodic architectures having different unit cell geometries like honeycomb, chiral, anti‐chiral, and re‐entrant^[^ ^45^, ^46^ ^]^ (refer to Figure 1B). These tubes can exhibit unprecedented mechanical properties due to their configuration and base lattice unit cell geometry, including a negative Poisson's ratio, low weight, resistance to indentation, shear resistance, fracture resistance, and high energy absorption capacity.^[^ ^47^, ^48^, ^49^, ^50^, ^51^, ^52^, ^53^ ^]^ However, since the main focus of this paper is origami‐inspired tubular metamaterials, we will limit our discussions concerning lattice‐based architectures here and introduce origami‐inspired metamaterials and their tubular architectures in the following paragraphs.

***Origami‐Inspired Metamaterials***. Just as lattice‐based metamaterials derive their unique properties from intricate beam, plate or shell‐based unit cells,^[^ ^12^ ^]^ origami and kirigami‐inspired metamaterials primarily exploit geometric principles and mechanics of folding to create periodic functional architectures. Origami and kirigami‐based metamaterials (refer to Figure 1C–E) have generated significant attention over the last few years due to their versatile attributes including deployability, shape morphing, conformal behavior, Poisson's ratio modulation, specific energy absorption capability, multi‐stability and constitutive law programmability.^[^ ^54^, ^55^, ^56^, ^57^, ^58^, ^59^, ^60^, ^61^, ^62^ ^]^ Origami, a traditional Japanese paper‐folding technique, involves the transformation of 2D sheets of material into intricate 3D architecture through the introduction of predefined creases for folding. In comparison, kirigami is a technique that involves cutting paper along with folding. Metamaterials are formed when such origami or kirigami base patterns are tessellated in one, two or 3D spaces to form a periodic architecture. Over time, for engineering applications of origami and kirigami, there has been a transition from traditional paper to a diverse array of materials such as metals, smart material alloys, polymers, and hydrogels, expanding the possibilities and functionalities of these origami and kirigami‐inspired metamaterials. Origami‐based tubes (refer to Figure 1D) are formed by folding a sheet of material into a tessellated architecture and often rolling it to give a hollow cylindrical shape, which leads to a range of enhanced mechanical and multi‐physical features. Our primary focus in this paper hereafter will be concentrated on such origami‐inspired tubular metamaterials.

***Unique Attributes of Tubular Origami Metamaterials***. Tubular origami metamaterials are a class of engineered materials that leverage the principles of origami folding patterns in thin sheets to create hollow cylindrical structures with unique mechanical, acoustic, or thermal properties. While the tubular geometry adds specific advantages as discussed in the following paragraph, origami tubes require more complex assembly methods (such as the use of adhesive materials to achieve structural integrity) unlike the seamless transformation achievable through pure folding techniques in conventional origami metamaterials.

**Figure 2:**
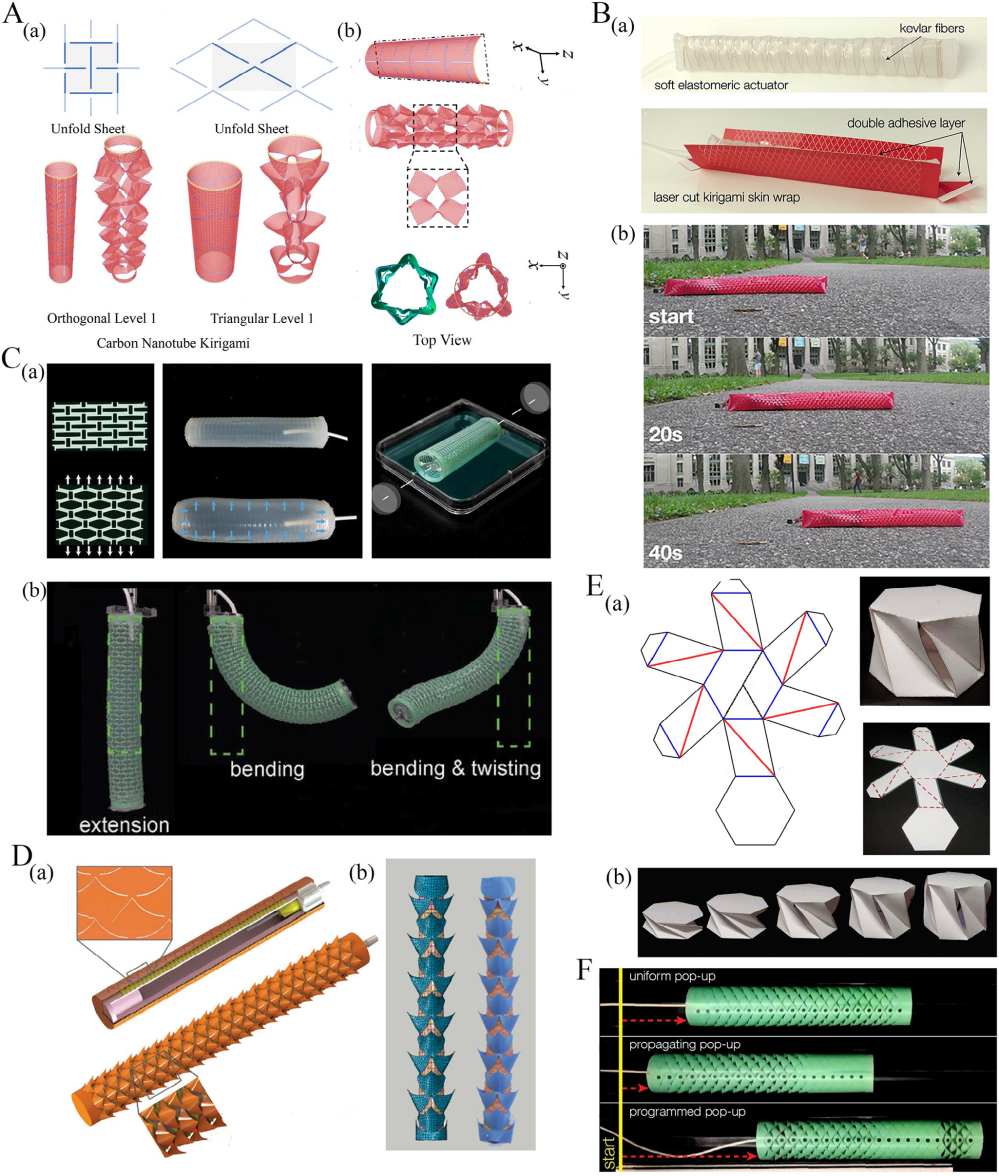
Kirigami‐based tubular metamaterials. A) Schematics of carbon nanotube kirigami (a) Level 1 carbon nanotube kirigami formed from orthogonal and triangular cuts (b) Symmetrical initial molecular dynamics model (Reproduced with permission.^[^ ^101^ ^]^ Copyright 2021, Elsevier). B) Kirigami skin‐based soft crawler (a) Kirigami skin, fabricated by embedding an array of cuts into a thin plastic sheet (b) Locomotion features of untethered kirigami‐skinned soft crawler (Reproduced with permission.^[^ ^102^ ^]^ Copyright 2018, The Authors, some rights reserved; exclusive licensee AAAS). C) Shape morphing of kirigami‐inspired inflatable metastructures showing (a) kirigami sheet deformation, elastomeric balloon inflation, and its fabrication process (b) Robotic deformation behavior with extension, bending, and bending‐twisting modes (Reproduced with permission.^[^ ^103^ ^]^ Copyright 2020, Wiley). D) Injectable kirigami stents showing (a) cut pattern and deployed configuration along with (b) FEM and experimental images of oesophageal stent (Reproduced with permission.^[^ ^70^ ^]^ Copyright 2021, Springer Nature Limited). E) Poly twist cell kinematic design showing valley (red), and mountain (blue) creases along with deployment stages (Reproduced with permission.^[^ ^104^ ^]^ Copyright 2017, Elsevier). F) Instability propagation in kirigami skin‐based tubes with triangular cuts showing uniform propagation and programmed pop‐up (Reproduced with permission.^[^ ^105^ ^]^ Copyright 2019, National Academy of Sciences).

Tubular origami metamaterials offer several distinct advantages over conventional origami metamaterials due to their tubular geometry and the specific design flexibility it provides. 1) The tubular design inherently provides greater structural integrity, resisting buckling and deformation under load better compared to flat or planar origami structures that are more prone to out‐of‐plane deformations and may lack the same level of structural robustness. 2) The closed or semi‐closed cross‐sectional geometry offers superior load distribution and higher specific stiffness and strength, depending on the applications. While lightweight, planar designs typically do not achieve the same level of specific strength and rigidity without additional supports or reinforcements. 3) Tubular origami structures excel at absorbing and dissipating energy (high specific energy absorption capability), making them ideal for crash protection, vibration damping, and impact mitigation. The planar origami designs may not offer the same degree of energy absorption, as they lack the enclosed geometry to effectively distribute and dissipate forces. 4) Tubular geometries efficiently use 3D space, providing high strength and functionality per unit volume compared to conventional planar origami metamaterials. 5) Tubular origami structures can often collapse and expand along a single axis, allowing for efficient packing (storage efficiency) and straightforward targeted deployment compared to conventional planar origami architectures. 6) Tubular origami designs can be anisotropic, providing tailored mechanical responses (e.g., stiffness or flexibility) along specific directions and achieving tailored Poisson's ratios along the axial direction. 7) Tubular origami architectures can accommodate multifunctionality, integrating structural support, fluid flow, and mechanical adaptability in a single design which can be crucial for developing robotic metamaterials with stationary applications and locomotion. In general, cylindrical or elongated shapes are better suited for constrained environments, such as medical stents, drug delivery, pipelines, inflatable booms, bellows or robotic actuators.^[^ ^63^, ^64^, ^65^, ^66^, ^67^ ^]^ 8) Tubular origami designs are more scalable for applications requiring larger structures, such as architecture or aerospace systems. In addition, tubular architectures offer a better scope for integrating these into the host structures, or compounding them in different ways to form more complex architectures for enhanced functionalities. 9) The principles of tubular origami metamaterials can further be extended to kirigami tubes involving both folding and cutting of the sheet of material. The incorporation of cuts in kirigami tubes enables localized expansion or contraction, thereby enhancing their flexibility and dynamic movement (refer to **Figure** **2**A–F), out‐of‐plane deformations, conformability and large‐scale shape morphing.^[^ ^68^, ^69^, ^70^ ^]^

***Scope of the Current Article***. The advantages of tubular origami metamaterials as discussed above make these architectures particularly suitable for engineering applications requiring high‐performance, compact, and multifunctional attributes, distinguishing them from conventional planar or non‐tubular origami designs. The primary aim of this article is to offer an insightful account of the recent advancements in artificially engineered tubular origami and kirigami metamaterials, with a particular emphasis on their multifunctionality across the length scales. In the following sections, we start with different classes of tubular origami metamaterials from the perspectives of functionality and origami base architecture. Subsequently, we delve into passive and active multi‐physical property modulation capabilities of tubular origami metamaterials, followed by in‐depth discussions on their computational modeling approaches and physical realizations (i.e. manufacturing). We have summarized the rapidly evolving, yet remarkable progress in this field concerning programmable constitutive behavior, multistability and modulation of energy landscape, shape morphing capabilities, conformable features, robotic motion, and dynamic actuation. This discussion then transitions into investigating mode coupling of normal and twisting deformations. Subsequently, we present an insightful perspective on the emerging trends and future roadmaps for the necessary research and innovation in the field of such tubular origami metamaterials and concluding remarks.

## 2 Emerging Classes of Tubular Origami Metamaterials

Origami metamaterials can typically be categorized according to their functionality and base architectural geometry. It is essential to emphasize that recent advancements in this field have shown that origami metamaterials are designed to achieve objectives across a wide range of physical domains, demonstrating their multifunctionality. This section provides a concise overview of various emerging classes of origami metamaterials focusing on tubular geometries.

### 2.1 Classification Based on Functionality

Functionality‐based metamaterials are formed with a focus on intentional utility and application. These materials are artificially engineered at micro‐ or nano‐scales to harness unprecedented properties at the macroscopic level. According to their intended functionalities, metamaterials are primarily categorized into optical metamaterials, electromagnetic metamaterials, acoustic metamaterials, and mechanical metamaterials. In this section, we present a concise overview of various types of functionally identified metamaterials, with a particular emphasis on origami/kirigami‐inspired mechanical metamaterials, aligning with the main theme of this article.

#### 2.1.1 Optical Metamaterials

Optical metamaterials represent a class of artificially engineered materials characterized by their ability to manipulate optical responses within a range of light frequencies. At a microscopic scale, these materials derive their distinctive properties not from their constituent materials but rather from the geometric parameters of subwavelength unit cells. Optical metamaterials possess extraordinary properties, including but not limited to negative refraction, invisibility cloaking, super‐resolution imaging, and energy harvesting.^[^ ^71^, ^72^, ^73^, ^74^, ^75^, ^76^ ^]^ However, their inherent rigidity typically leads to poor flexibility and tunability. Such limitation is addressed by the incorporation of soft materials into metamaterial compositions or the development of responsive metamaterial architectures. It allows the possibility of structural reconfiguration of optical metamaterials through various external stimuli, including optical, thermal, electrical, and mechanical, thereby unlocking novel programmable optical functionalities and expanding their potential applications.^[^ ^77^ ^]^

Origami‐based optical metamaterials integrate the principles of origami folding with metamaterials to achieve expanded functionalities. Origami‐based optical metamaterials can introduce controllable optical properties and structural deployability based on the degree of folding and orientation of creases, leading to advanced applications and functionalities for optical devices, cloaking, and superlens.^[^ ^78^, ^79^, ^80^, ^81^ ^]^

#### 2.1.2 Electromagnetic Metamaterials

Electromagnetic metamaterials are artificially engineered materials comprised of periodic structural arrangements, which show distinctive properties that are absent in natural materials and exhibit unusual electromagnetic responses within specific frequency bands. These metamaterials are characterized by their uniformity, in which the structural unit cell dimensions are smaller than the wavelengths of guided electromagnetic waves. A prominent feature of these materials is that they can possess negative magnetic permeability and negative electric permittivity. Negative magnetic permeability allows the material to form a magnetic dipole opposite in direction to the applied magnetic field, while negative electric permittivity indicates that the electric field and electric displacement vector oppose one another. Such electromagnetic materials find practical utility in diverse applications, including perfect lenses, electromagnetic cloaks, and a variety of non‐destructive testing sensors.^[^ ^82^, ^83^, ^84^, ^85^, ^86^ ^]^

Origami‐based electromagnetic metamaterials can manipulate electromagnetic waves in real time based on folding percentage and origami crease configuration. Further, the aspects of deployability and compact storage are critical for a range of applications. These metamaterials possess negative refractive index, tuneability, frequency selectivity, cloaking, and controlled wave propagation properties, leading to a wide range of applications, including invisibility cloaking, antenna, absorbers, sensors, and energy harvesting devices.^[^ ^87^, ^88^, ^89^, ^90^, ^91^, ^92^ ^]^

#### 2.1.3 Acoustic Metamaterials

Acoustic metamaterials represent artificially engineered materials tailored to govern, direct, and modulate sound waves or phonons across a variety of media, including gases, liquids, and solids. These metamaterials are characterized by the capability to exhibit unconventional properties, such as a negative bulk modulus^[^ ^93^ ^]^ or negative bulk density.^[^ ^94^ ^]^ The emergence of double negative refraction^[^ ^95^ ^]^ phenomena occurs when both the bulk modulus and bulk density of the metamaterial are negative. These distinctive characteristics arise from the precise manipulation of monopole and dipole resonances within the material structure. Further, the utilization of external control mechanisms enables the understanding of effective metamaterial properties that exceed those inherent in passive materials. Therefore, this field of research has accelerated the development of dynamically reconfigurable, loss‐compensating, and parity‐time symmetric materials, which increase the possibilities to extend the boundaries of sound manipulation.^[^ ^96^ ^]^ The utilization of acoustic metamaterials extends across a wide range of applications, from acoustic cloaking and noise shelters^[^ ^97^, ^98^ ^]^ to acoustic imaging and superlensing techniques.^[^ ^99^, ^100^ ^]^

Origami‐based acoustic metamaterials involve creating structures with specific crease pattern arrangements to manipulate sound waves in a unique and on‐demand manner as a function of different folded configurations along the motion path depending on the origami crease pattern. These metamaterials possess negative density, active noise control, and shape reconfiguration, leading to their wide range of advanced applications in acoustic imaging, phononic crystal lenses and soundproof devices.^[^ ^106^, ^107^, ^108^, ^109^, ^110^ ^]^

#### 2.1.4 Mechanical Metamaterials

In the past decade, inspired by the other classes of metamaterials, mechanical metamaterials have emerged as a distinct category of metamaterials showcasing remarkable mechanical and multi‐physical properties as a function of their artificially engineered micro‐ or nano‐architectures. This field has swiftly evolved from its initial aim of achieving unconventional values for well‐known physical parameters such as Poisson's ratio, bulk density, and bulk modulus to the evolution of novel classes of mechanical metamaterials, offering unprecedented functionalities and programmable constitutive laws along with paving the way for the development of advanced materials with tailored properties. While two and 3D lattice metamaterials have multi‐physical characteristics extending over multiple length scales, thereby making them suitable for application‐specific needs,^[^ ^28^, ^111^, ^112^, ^113^, ^114^, ^115^, ^116^ ^]^ origami‐inspired mechanical metamaterials have emerged rapidly over the last few years. In the following paragraphs, we further delve into origami and kirigami‐based metamaterials as per the focus of this article.

As denoted by their nomenclature, origami and kirigami‐inspired metamaterials utilize the principles of folding (and cutting in the case of kirigami) along with periodically assembling planar or two‐dimensional materials into complex 3D structures. The configurations of these structures are primarily defined by two fundamental parameters: creases and vertices, while their overall forms are governed by the magnitude, quantity, sequence, and orientation of the folds (and cuts, additionally in case of kirigami).^[^ ^117^, ^118^ ^]^ Some notable examples of origami‐inspired metamaterials include patterns such as Miura‐ori and its derivatives,^[^ ^119^, ^120^ ^]^ Kresling,^[^ ^121^, ^122^ ^]^ waterbomb,^[^ ^123^, ^124^ ^]^ chiral origami,^[^ ^125^ ^]^ Yoshimura,^[^ ^126^ ^]^ Egg box,^[^ ^127^ ^]^ Square twist,^[^ ^128^ ^]^ Tachi–Miura Polyhedron (TMP),^[^ ^129^ ^]^ and Ron Resch origami,^[^ ^130^ ^]^ each offering distinct mechanical characteristics and deformation behaviors suited for various engineering applications.

Origami‐based metamaterials are classified into two principal categories based on the landscape of elastic energy during folding and unfolding processes: rigid origami and deformable origami. Rigid origami involves folding and unfolding along the creases without inducing deformation within the panel, with energy predominantly stored within the folding creases. For origami to be rigid, three criteria need to be fulfilled: 1) the pattern must be mathematically foldable; 2) the panel must be much stiffer than the crease; 3) the material should be subjected to uniform loading along the folding and unfolding direction, rather than concentrated, bending and twisting. In comparison, deformable origami involves energy storage within creases and panels throughout folding (i.e. the panels also fold along with creases). In rigid origami, although the panels are theoretically supposed to remain rigid during folding, they may still deform in reality. As a result, these patterns are sometime categorised as deformable rigid origami. In contrast, for other patterns that do not fold according to mathematical principles, the panels must undergo deformation in order to be folded.

In contrast to origami‐based metamaterials, kirigami‐based metamaterials employ the fundamental concept of paper cutting along with crease folding, wherein tessellation of unit cell patterns can lead to mechanical metamaterials designed for targeted functionalities. Often regarded as an extension of origami, kirigami distinguishes itself by incorporating cutting processes that result in distinctive features such as the ability to undergo out‐of‐plane buckling and in‐plane rotation, large deformation and morphing, alleviating stress concentration, and enabling the fabrication of complex 3D configurations. Kirigami‐based metamaterials are classified into two main types based on their deformation mechanisms: rigid kirigami (refer to Figure 2C,D) and deformable kirigami (refer to Figure 2B, E, F). Rigid kirigami is developed when the linkages are relatively small and rigid compared to the panels. In contrast, deformable kirigami involves structural deformation, in which rigid body motion coexists with bending strains among the panels.^[^ ^131^, ^132^, ^133^, ^134^, ^135^ ^]^

The coupling of origami and kirigami techniques results in hybrid metamaterials which often require assembling kirigami or origami modules.^[^ ^136^ ^]^ Hybrid origami and kirigami‐based metamaterials employ the concept of both cutting and folding. These hybrid metamaterials can be further divided into rigid and deformable architectures based on elastic energy landscape. In general, origami and kirigami‐based metamaterials utilize the plate‐link mechanism, where plates correspond to faces and hinges act like creases. This mechanism uses minimal energy while allowing unconstrained motion. Origami/kirigami‐based metamaterials exhibit unprecedented properties, such as stiffness modulation including contact‐driven nonlinear programming, negative Poisson ratio, shape morphing capabilities, tunability of different physical properties, multistability, and efficient energy dissipation.^[^ ^137^, ^138^, ^139^, ^140^, ^141^, ^142^ ^]^ The principles of origami and kirigami are often analogous to the design of compliant mechanisms (rigid origami and kirigami are modeled with creases considered joints and facets considered links), where simplicity leads to complex and rich functionality.^[^ ^143^, ^144^, ^145^, ^146^, ^147^, ^148^ ^]^ The inherent characteristics of origami and kirigami‐based metamaterials make them intriguing choices for a wide range of applications, including mechanical parts, deployable structures, electronics, robotics, biomedical devices, and shape‐morphing materials.

### 2.2 Classification Based on Origami Architecture

**Figure 3:**
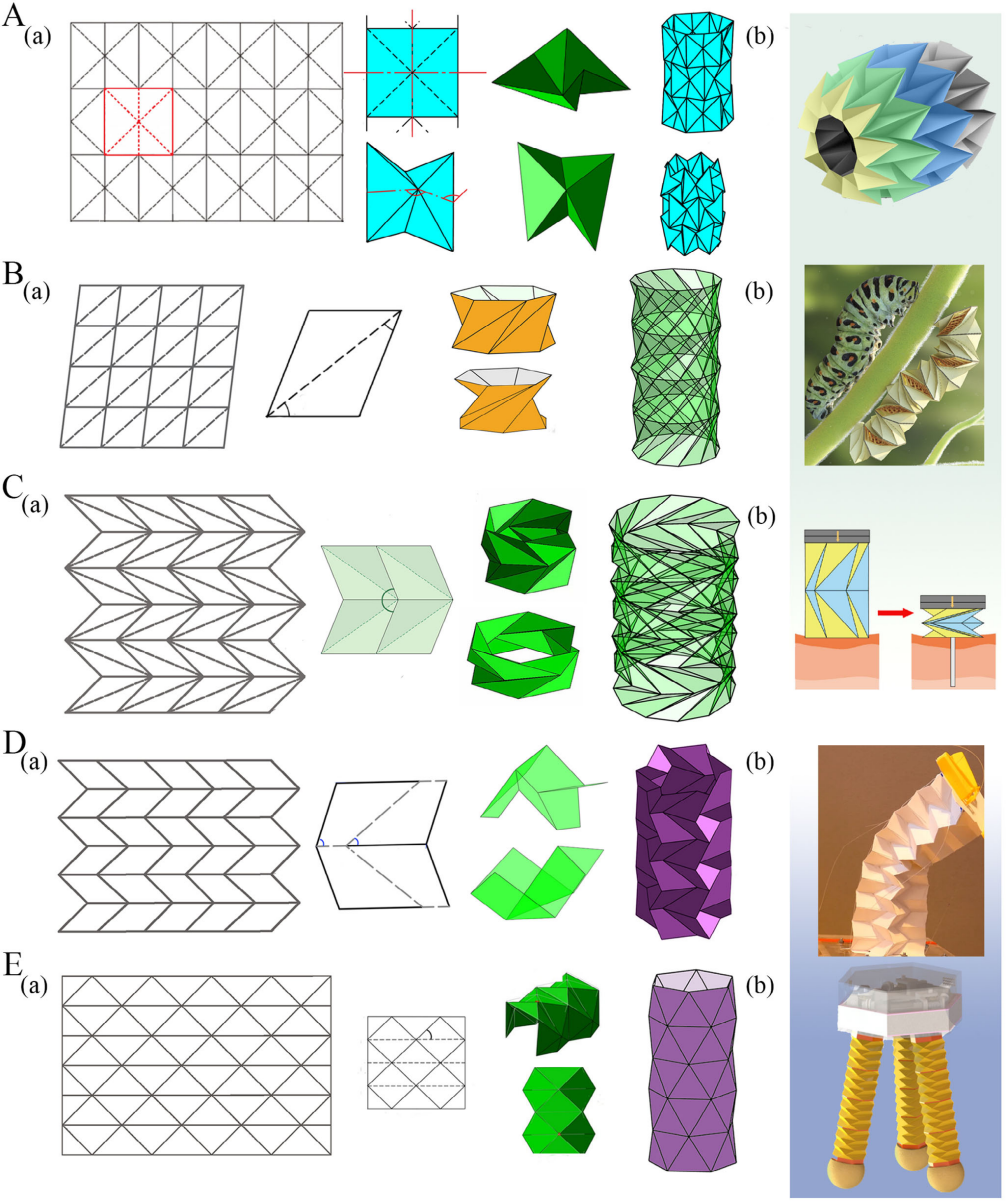
Crease architecture of typical tubular metamaterials. A) Waterbomb tube (a) Crease pattern, unit cell folding, and tubular configuration (b) Waterbomb‐based wheel of origami robot for autonomous navigation (Reproduced under the terms of the CC‐BY 4.0 license.^[^ ^149^ ^]^ Copyright 2024, The Authors). B) Kresling I tube (a) Crease pattern, unit cell folding, and tubular configuration (b) Kresling I based untethered origami robot resembling a caterpillar reprogrammed by electro‐thermal actuation (Reproduced with permission.^[^ ^150^ ^]^ Copyright 2024, National Academy of Sciences). C) Kresling II tube (a) Crease pattern, unit cell folding, and tubular configuration (b) Kresling II based magnetically actuated biopsy robot (Reproduced with permission.^[^ ^151^ ^]^ Copyright 2024, Elsevier). D) Miura‐ori tube (a) Crease pattern, unit cell folding, and tubular configuration (b) Miura‐based tendon actuated reconfigured robotic manipulator (Reproduced with permission under the terms of the CC‐BY 4.0 license.^[^ ^152^ ^]^ Copyright 2021, The Authors, IEEE). E) Yoshimura tube (a) Crease pattern, unit cell folding, and tubular configuration (b) Yoshimura tube‐based three‐limbs hybrid origami gripper (Reproduced under the terms of the CC‐BY 4.0 license.^[^ ^153^ ^]^ Copyright 2024, Exclusive licensee Beijing Institute of Technology Press. No claim to original U.S. Government Works).

Origami‐based metamaterials can be categorized according to their architectural configurations, described by distinct crease patterns that lead to tunable and programmable mechanical behavior. A range of base unit patterns, either adopted from conventional origami art, or designed through the intuitive understanding of folding mechanics and algorithmically designed crease orientations can be adopted for developing a range of planar and tubular metamaterials.^[^ ^54^ ^]^ Prominent examples of origami unit cells exploited in tubular origami metamaterials include the Miura‐ori and its derivatives, Kresling, waterbomb, and Yoshimura (refer to **Figure** **3**). In this section, we present a brief introduction to the crease pattern of different tubular origami architectures along with a notion of the scope of applications. Although it is imperative to emphasize that the applications described herein reflects a subset of the countless engineering possibilities inherent to this class of metamaterials.

***Miura‐Ori Based Tubular Origami***. In recent years, the utilization of origami folding patterns has expanded across various disciplines, particularly drawing attention to rigid origami configurations. Rigid origami is a subset that enables seamless transitions between folding states without requiring bending or twisting motions. Among these, the Miura‐ori pattern emerges as a fundamental rigid structure, popular for its suitability in engineering applications owing to inherent properties such as negative Poisson's ratio, single degree of freedom, deployability, and foldability. The Miura‐ori unit cell is composed of four trapezoids arranged adjacent to each other. This unit cell comprises a total of four creases, with one serving as mountain creases (represented by solid lines) and the remaining three as valley creases (represented by dotted lines). Additionally, the valley creases adjacent to two collinear creases do not align parallel to the edge creases (refer to Figure 3D(a)). Through repetitive tessellation in both longitudinal and circumferential directions, Miura‐ori unit cells form tubular structures known as Miura‐ori tubes. This origami tube can be constructed by rigid panels only where deformation only occurs along the crease lines, leading to strict axial folding. While these tubes may lack rigidity in folding depending on the mechanical behavior of creases, they exhibit multistability.^[^ ^154^, ^155^ ^]^ The concepts of Miura‐ori tubes are further extended to polygonal and translational symmetric cross‐sections, that can reconfigure into different geometries while maintaining flat and rigid foldability. The proposed tubes may not be straight and can be constructed to follow a nonlinear curved line when deployed as per application‐specific requirements.^[^ ^21^ ^]^

Among their potential applications, Miura‐ori tube‐based tendon‐actuated robotic manipulators stand out for their reconfigurability, facilitating convenient storage and deployment.^[^ ^152^ ^]^ Other applications with appropriate modifications include multi‐limb jugglers for overcoming challenges in dynamical dexterity for robots.^[^ ^156^ ^]^

***Waterbomb Based Tubular Origami***. Waterbomb unit cells, as typically adopted in tubular metamaterials, comprise of a square sheet of material having six creases: four diagonal creases acting as valley creases intersecting at a common vertex and two collinear sides acting as mountain creases. Through repetitive tessellation of the waterbomb unit cell in both longitudinal and circumferential directions, a waterbomb tube is formed, exhibiting rich mechanical characteristics^[^ ^124^ ^]^ in terms of programming constitutive laws, auxetic behavior and shape modulation (refer to Figure 3A). Additionally, the symmetrical folding of the waterbomb tube makes it a single‐degree‐of‐freedom system^[^ ^123^, ^157^, ^158^, ^159^ ^]^ that requires limited actuation. Interestingly, it may be noted that since each of the waterbomb units is inherently bistable along the radial direction, it leads to a possibility of further programming the mechanical behavior based on selective stable state distribution.

**Figure 4:**
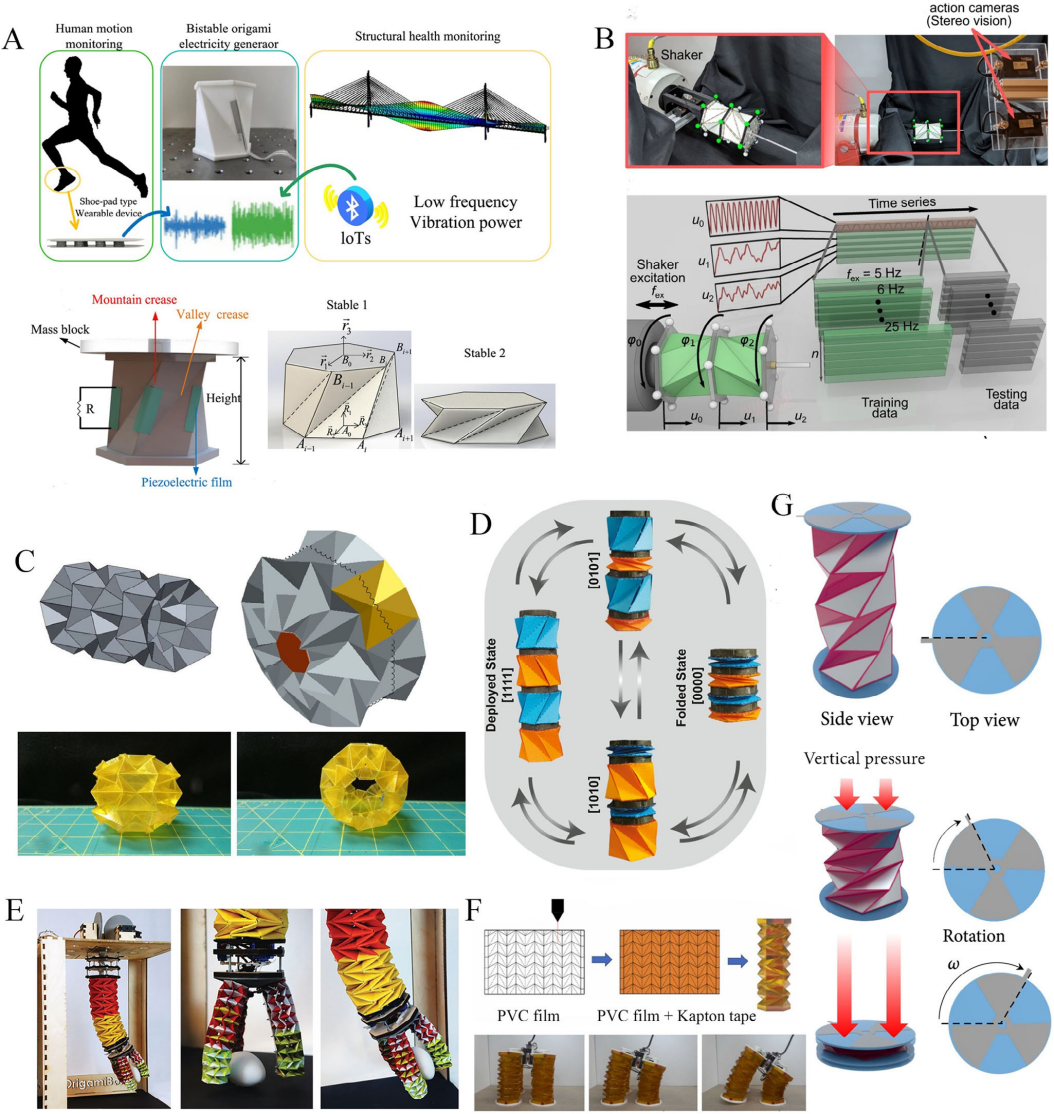
Multiple functionalities of tubular origami metamaterials. A) Bistable Kresling origami energy generator for wearable devices and bridge monitoring devices (Reproduced with permission.^[^ ^172^ ^]^ Copyright 2022, Elsevier). B) Two triangulated cylindrical origami used in a vibrating environment to analyse and control the dynamic behavior (Reproduced under the terms of CC‐BY 4.0 license.^[^ ^173^ ^]^ Copyright 2020, The Authors). C) Waterbomb origami wheel with shape memory alloy actuator for variable functionalities (Reproduced with permission.^[^ ^161^ ^]^ Copyright 2020, National Academy of Sciences). D) Untethered origami microrobots with magnetic actuation^[^ ^174^ ^]^ E) Three‐finger origami‐based robotic manipulator (Reproduced with permission.^[^ ^162^ ^]^ Copyright 2017, Cambridge University Press). F) A quadrupedal robot with tendon‐driven origami legs (Reproduced with permission.^[^ ^156^ ^]^ Copyright 2024, Elsevier). G) A piezoelectric/triboelectric energy generator based on triangulated origami cylinders (Reproduced under the terms of CC‐BY 4.0 license.^[^ ^175^ ^]^ Copyright 2021, The Authors, exclusive Licensee Science and Technology Review Publishing House).

Waterbomb tubes find critical applications in the construction of origami robots, medical devices^[^ ^160^ ^]^ and the design of wheels suitable for autonomous navigation.^[^ ^149^ ^]^ Fonseca et al. proposed an expandable origami wheel, constructed based on the fundamental principles of the waterbomb pattern undergoing transitions between various configurations via thermal actuation enabled by shape memory alloy actuators.^[^ ^161^ ^]^ Its deployment gives rise to a lot of benefits, including trajectory adjustments through alteration of configuration, obstacle traversal capabilities, and enhanced stability during rolling movements (refer to **Figure** **4**C). Li et al. proposed the architecture of simple soft gripper involving waterbomb origami^[^ ^162^ ^]^. This gripper is evaluated by capturing a range of everyday objects as well as a set of test objects with diverse geometries.

***Yoshimura‐Based Tubular Origami***. The Yoshimura tube constitutes an origami structure derived from the crease pattern created by the tessellation of Yoshimura‐based unit cells. These unit cells consist of two diamond shapes linked at a shared vertex. Yoshimura unit cell possesses ten creases, with eight corresponding to mountain folds and the remaining two corresponding to valley creases. The tessellation process of the Yoshimura tube involves the replication of a fundamental unit cell along both longitudinal and circumferential directions (refer to Figure 3E(a)). Yoshimura tubes possess the characteristic of non‐rigid foldability, and monostability.^[^ ^163^, ^164^, ^165^, ^166^ ^]^

A notable application of Yoshimura tubes lies in the realm of robotic manipulation, demonstrated by the three‐limbed hybrid origami gripper,^[^ ^153^ ^]^ which showcases an exceptional capacity to modulate finger stiffness in response to alterations in internal pressure and finger length in the manipulation of cable length adjustment (refer to Figure 3E). A variant of the Yoshimura tube, termed the modified Yoshimura pattern, maintains the basic Yoshimura structure, but with a notable distance i.e., the two diamonds are spatially separated rather than being connected at a common vertex.^[^ ^167^ ^]^

***Kresling Origami Tubes***. One of the most versatile bases in the design of origami tubes is Kresling which can show a range of programmable mechanical characteristics based on the crease pattern and assembly. We have referred to the two primary Kresling patterns here as Kresling I and II. The Kresling I base consists of a parallelogram sheet designated by five creases, with four sides of the parallelogram acting as mountain folds and one vertical diagonal functioning as a valley fold. Through repetitive tessellation of Kresling I unit cells in both longitudinal and circumferential directions, a Kresling I tube is formed (refer to Figure 3B(a)). Notably, the Kresling I tube lacks rigid foldability and cannot deviate from its nominal expanded state through crease manipulation. Upon reaching its fully folded state, the upper and lower triangular planes align with each other. A distinguishing characteristic of the Kresling I tube lies in its multi‐stability, which depends upon the tube's height and the length of its diagonal.^[^ ^168^, ^169^, ^170^, ^171^ ^]^ One notable application of the Kresling I structure is in the development of multi‐degree‐of‐freedom origami robots that achieve motion by electro‐thermal actuation, resembling a caterpillar.^[^ ^150^ ^]^ This technology offers several advantages, including its lightweight design, ability to execute steering motions by assembling with other robotic units and cargo transportation (refer to Figure 3B(b)).

A modified version of the Kresling I tube involves the unit cell composed of a congruent parallelogram with two diagonals, resulting in six creases.^[^ ^176^ ^]^ Four of these creases indicate the sides of the parallelogram, corresponding to mountain folds, while one diagonal acts as a valley fold, and the other diagonal serves as a mountain fold. This arrangement enables the formation of modified Kresling I tubes which can exhibit multi‐stability.

The Kresling II pattern draws inspiration from the geometric structure of unfolded tree leaves. It is characterized by a tessellation process where Kresling II bases are repetitively arranged longitudinally and circumferentially to form a tube. Kresling II base comprises two identical parallelograms positioned adjacent with sharing a common side and it additionally includes two vertical diagonals in opposite alignment converging at a common vertex. Kresling II unit cells comprise nine creases, with all seven sides acting as mountain creases and the two diagonals acting as valley creases (refer to Figure 3C(a)). Kresling II tubes exhibit partial flat folding exclusively at the region of valley creases, while remaining essentially 3D in already bent regions^[^ ^177^, ^178^, ^179^, ^180^ ^]^ However, it lacks rigid foldability and can exhibit mono‐stability and multi‐stability depending on the geometric parameters. Among its practical applications, the Kresling II design can find its utility in the development of magnetically actuated biopsy robot.^[^ ^151^ ^]^ In this application, the proposed structure enables the robot to fold, deploy the needle into the targeted tissue, effectively capture the tissue and provide the retraction force when external magnetic force is removed. The permanent magnet on the top of the robot generates magnetic force and torque in response to external magnetic field, allowing the robot to effectively perform the rolling and sampling motion (refer to Figure 3C(b)). Another application of the Kresling II design is a quadrupedal soft robot.^[^ ^181^ ^]^ This robot is constructed using four Kresling II pattern origami cylinders as legs, utilising their high axial stiffness for effective locomotion. Furthermore, a tendon‐driven actuation mechanism is used to fold and extend the legs using two motors, requiring no external device. This design ensures durability and efficient force transmission, resulting in faster movement. Additionally, the origami structure helps absorb shocks during operation (refer to Figure 4F).

Huang et al. proposed a flexible wearable power demonstration featuring an origami‐based shoe pad engineered to harvest energy from the cyclic motion of the human body. This energy harvesting mechanism holds considerable potential in harvesting power extraction efficiency, thereby enabling the realization of self‐powered devices optimized for human motion or structural health monitoring applications (refer to Figure 4A)^[^ ^172^ ^]^ Yasuda et al. proposed triangulated origami cylinders for vibration analysis and dynamic characteristics of structures, Chung et al. demonstrated these cylinders to serve as piezoelectric generators (refer to Figure 4B,G),^[^ ^173^, ^175^ ^]^ while Jeong et al. developed an origami‐inspired twisted tower‐based three‐finger robotic manipulator (refer to Figure 4E).^[^ ^182^ ^]^ Further applications based on Kresling origami include untethered origami robots propelled by magnetic actuation (refer to Figure 4D).^[^ ^174^ ^]^ Masana et al. proposed a memory switch based on Kresling origami that can be activated by providing harmonic mechanical excitation at its base.^[^ ^183^ ^]^ The memory switch can move between states by applying harmonic excitation with specific frequencies and amplitudes unique to each state of the switch. These parameters vary for switches with different design characteristics. Yasuda et al. developed non‐volatile mechanical memory storage devices inspired by Kresling origami.^[^ ^184^ ^]^ When two monostable Kresling origami springs with opposite chirality are joined at their ends, they form a mechanical bit. The two binary states of the bits correspond to the two distinct stable states of the Kresling origami spring, under specific prestress levels. Additionally, the bit can be actuated using a controlled torsional input at one end.

***Supplementary Note on Thick‐Panel Origami***. Traditionally, origami‐based models are mostly created from thin sheets, resulting in panels with negligible thickness compared to other dimensions. These origami are known as thin origami. Thin origami suffers from many engineering challenges for large‐scale applications, such as less stiffness and structural failure due to large self‐weight and surrounding loads. In these practical engineering applications, the thickness of the facets can not be ignored, so the need for thick panels of origami, known as thick origami, becomes necessary. Thick origami not only maintains structural integrity but also provides better stiffness and developability to the structure. A wide range of methods are proposed to fold the thick panels, like the offset at the edges of panels, replacement of fold with parallel panels, and use of tapered surface.^[^ ^185^, ^186^, ^187^, ^188^, ^189^, ^190^ ^]^

## 3 Multi‐Physical Functionality of Tubular Metamaterials

Tubular origami metamaterials have drawn tremendous interest from the scientific community in recent years due to their exciting multi‐functional mechanical capabilities, such as stiffness and shape modulation, rich dynamic properties, energy absorption, and robotic motion (both stationary and locomotion) along with active control of the physical properties. In this section, we will delve deeper into the multi‐physical properties of origami‐based tubular metamaterials.

### 3.1 Target‐Oriented Mechanical Functionality

In the case of traditional lattice‐based metamaterials, passive metamaterials can be defined as the lattice architecture in which alteration (i.e. on‐demand modulation) of properties can only be done before the fabrication process.^[^ ^12^ ^]^ Once these conventional lattices are manufactured as per designed unit cell geometry, the properties normally become fixed. In the case of active lattices (such as magneto‐active and piezo‐active architectures), the mechanical properties can be modulated following an on‐demand programmable approach. However, for origami‐based metamaterials, mechanical properties can often be modulated post‐manufacturing as a function of crease‐level folding percentage (i.e. different states of the motion path at different folding angles) as origami‐based architectures normally have large deformation with significant change in the shape, and therefore in the structural matrices (such as stiffness and mass matrix). In this section, we emphasize different functional characteristics of tubular origami architectures such as constitutive behavior including multi‐stability and deformation mode coupling, shape morphing, energy absorption, robotic motion and dynamic characteristics.^[^ ^12^, ^124^, ^191^, ^192^ ^]^

#### 3.1.1 Programming Constitutive Behavior and Multi‐Stability

Origami architectures have gained significant attention as potential candidates for designing metamaterials with programmable elastic properties, including deformation, stiffness, and multistability. These metamaterials and metastructures (note that these two terms are interchangeably used in many instances in the literature, but there exists a subtle difference as explained in the introduction section) can be adaptable and responsive to external loading, boundary conditions, and the prevailing physical environment in which they operate. The programmable constitutive behavior of origami metamaterials primarily depends upon the details of the crease pattern and the arrangement of mountain and valley creases. Here we focus on the tubular origami architectures with unprecedented mechanical constitutive relationships.

**Figure 5:**
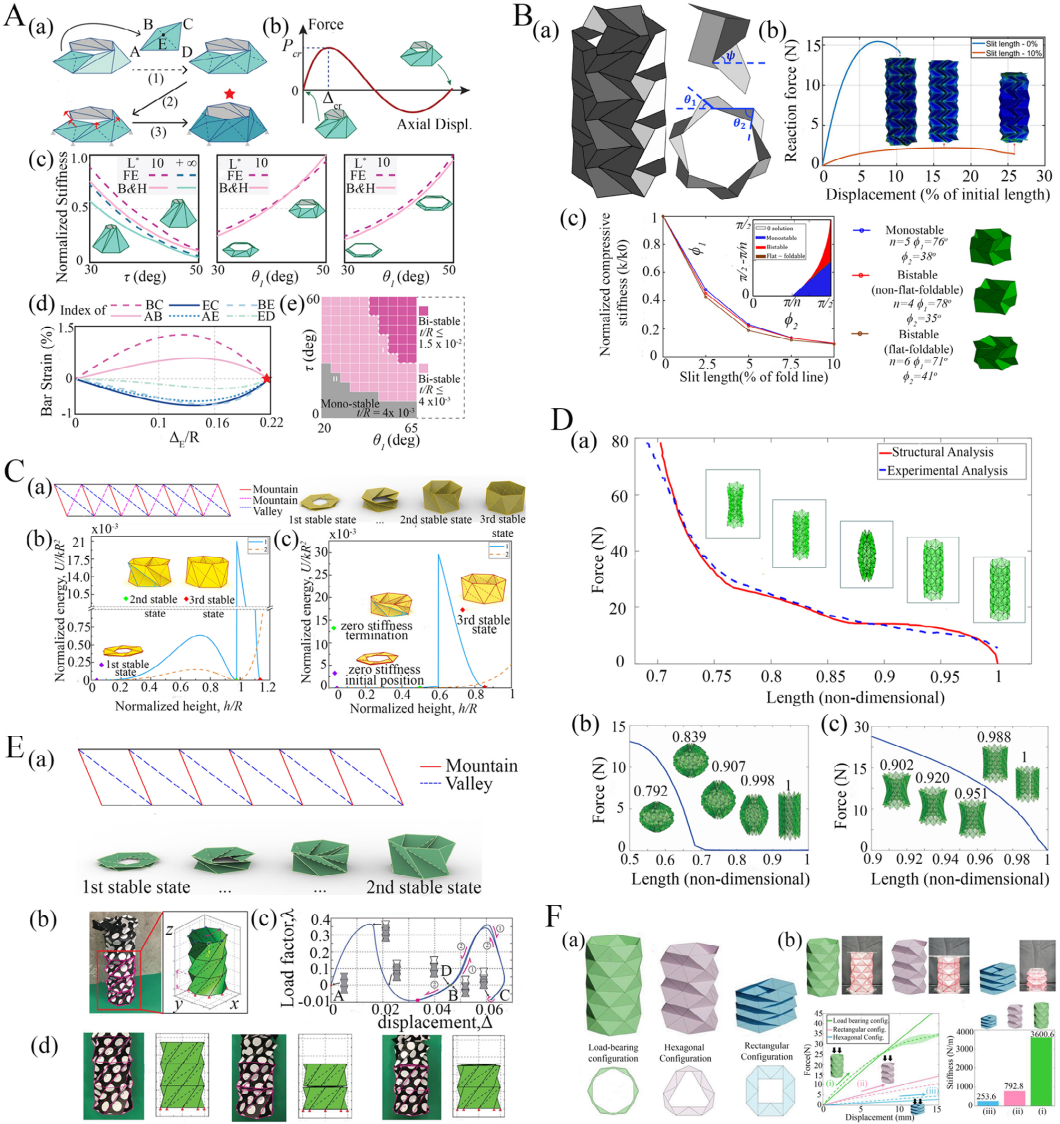
Programmable constitutive behavior and multi‐stability characterization. A) Multi‐stable Kresling tubes with conical geometry (a) Geometric features (b‐c) Axial loading and normalized stiffness response showing multi‐stability (d‐e) Numerical prediction of bar strain and multi‐stable behavior of pop‐up configuration (Reproduced with permission.^[^ ^193^ ^]^ Copyright 2022, Elsevier). B) Axial loading response and multi‐stable behavior of origami bellow (a‐b) Miura‐ori bellow and its mechanical response with varying slit length (c) Representation of mono‐stable, bi‐stable, and bi‐stable flat‐foldable behavior of Miura‐ori bellow (Reproduced under the terms of the CC‐BY 4.0 license.^[^ ^194^ ^]^ Copyright 2023, The Authors). C) Multiple triangles cylindrical origami geometric and multi‐stability features (a) Schematic diagram of crease pattern and folding configuration (b) Normalised energy vs normalized height response to express the tri‐stable state (Reproduced under the terms of the CC‐BY 4.0 license.^[^ ^176^ ^]^ Copyright 2023, The Authors). D) Programmable shape and (contact‐driven) stiffness modulation of waterbomb origami tubes through varying the number of rows and columns (Reproduced with permission.^[^ ^124^ ^]^ Copyright 2019, Elsevier). E) Large‐scale shape morphing of Kresling origami tubes showing the crease architecture, folding behavior, load‐displacement constitutive behavior and multi‐stable states (Reproduced with permission.^[^ ^195^ ^]^ Copyright 2017, The Royal Society). F) Self‐reconfigurable Yoshimura origami tubes with multiple configurations (a) Geometric representation of load‐bearing, hexagonal and rectangular configurations (b) Origami‐based structure prototypes made from shape memory alloy material exhibiting self‐reconfigurability and stiffening features (Reproduced with permission.^[^ ^196^ ^]^ Copyright 2022, Wiley).

Waterbomb‐inspired tubular origami metamaterials have garnered significant attention due to their distinctive crease pattern and negative Poisson ratio.^[^ ^197^ ^]^ Mukhopadhyay et al. conducted a comprehensive investigation, including structural and experimental simulation, to analyze the mechanical constitutive behavior of the waterbomb tubes.^[^ ^124^ ^]^ The waterbomb tube is initially parameterized using a uniform radius along its length, where length is taken as a dimensionless ratio of change in length to original length.^[^ ^158^ ^]^ The investigation showed that the constitutive curve exhibits a marked increase in stiffness at a specific non‐dimensional deformation, showing the transition from pure rigid motion to structural deformation initiated by vertex contact (refer to **Figure** **5**Da). Interestingly, such force‐deformation constitutive curves can be programmed to have a sudden rise in stiffness by designing such contact based on the waterbomb origami architecture. This allows us to design megastructures where high stiffness can be provided in the deformation process when it is needed for most optimal designs. Note that it is a significant parallel achievement in the general domain of architected materials and structures where it is normally possible to have spatially variable stiffness based on graded unit cells, leading to zones of high and low stiffness as necessary. Furthermore, the investigation of the spatial profile of the waterbomb tube showed that with a decrease in sector angles, the tube forms a single concave surface, while with an increase in sector angle induces a curvature reversal, resulting in a concave surface formation. It is also possible to have a target configuration of bulges or waviness profiles in such origami tubes.^[^ ^124^, ^198^ ^]^ The findings of these investigations suggest that far‐field force can effectively alter the surface waviness without the need for local controllers or actuators, leading to tailorable‐action‐at‐a‐distance metamaterials (refer to Figure 5D(b,c).

An intriguing property in certain origami tubular architectures is bi‐stability or multi‐stability, depending on the tubular configuration and number of rows (or storeys). Multi‐stability in tubular origami metamaterials is the ability of the structure to maintain multiple stable configurations during the deformation process. In traditional mono‐stable origami, the final form of the structure is fixed and pre‐defined (a single stable state at the rest angle configuration), while it deforms continuously under applied external loads. In contrast, the multi‐stable origami structures can seamlessly show the transition between various shapes or states while maintaining stability in each configuration.^[^ ^138^, ^199^, ^200^, ^201^, ^202^, ^203^ ^]^ For transitioning from one stable state to the other, an energy barrier needs to be crossed through the application of external energy. In the following paragraphs, we delve deeper into the constitutive behavior and multi‐stable properties exhibited by origami‐based tubular metamaterials.

We start with an understanding of the constitutive behavior of the simplest origami, the Miura‐ori. Miura‐ori‐based sheets show in‐plane stiffness and continuous deformation when the panels of Miura‐ori origami are rigid. At the same time, Miura‐ori sheets can show out‐of‐plane deformation and bending when the panels are non‐rigid. Miura‐ori‐based sheets also exhibit local bi‐stability, a stable pop‐up configuration that occurs when a vertical load is applied to the vertex of the Miura‐ori sheet.^[^ ^55^, ^204^, ^205^, ^206^ ^]^ The arrangement of these origami sheets in a cylindrical form results in the formation of the origami tubes or bellows.^[^ ^194^ ^]^ The Miura‐ori tube unit cell, inspired by the alternation of creases of simple Miura‐ori pattern, has undergone extensive exploration and application across various domains (refer to Figure 5B(a). The mechanical characteristics of Miura‐ori bellow was investigated by Schenk et.al by introducing a slit into the miura bellow pattern, maintaining planar boundary conditions at the top and bottom ends to provide contraction and expansion during compression. It has been observed in this investigation that the incorporation of a slit^[^ ^207^, ^208^ ^]^ induces a non‐linear response and reduces the axial stiffness relative to slit‐free bellows. In addition, as the slit increases from 0 to 10% of the fold length of the pattern, compressive properties experience a sharp decline (refer to Figure 5B(b). Furthermore, varying the sector angle and the number of Miura‐ori units in the circumferential direction resulted in mono‐stable, non‐flat foldable bi‐stable, and flat foldable bi‐stable configurations of the Miura bellow (refer to Figure 5B(c).

Another compelling origami metamaterial is the Kresling pattern, which is a variant of cylinder shell origami that exhibits multi‐stable characteristics. Liu et al. developed a foldable bag which was fabricated from a Kresling pattern, and was capable of forming stable structures in both folded and deployed states (refer to Figure 5E(a,b).^[^ ^195^ ^]^ The multi‐stable characteristics of the Kresling cylinder are realised by the variation in crease length.^[^ ^209^ ^]^ Additionally, the multi‐stable behavior of the Kresling cylinder arises from panel stretching rather than panel bending. When the Kresling cylinder was subjected to uniform unit compression at the apex nodes, the Kresling cylinder initiated deformation from point A and assumed multi‐stable states at points B and C before retracing its path to point D (refer to Figure 5E(c). A variety of multi‐stable states of the Kresling cylinder are shown in Figure 5E(d). Kresling patterns can be extended to diverse modifications, including alterations in crease alignment and orientation. These modified versions also exhibit bi‐stability and multi‐stability (with programmable energy barriers among the stable states), thus opening the way for numerous engineering applications. We will now discuss some of these modified versions of the Kresling pattern.

Wo et al. developed Kresling origami‐based corrugated tube, which draws its inspiration from the flexibility observed in drinking straws.^[^ ^193^ ^]^ The fundamental unit of the corrugated tube comprises two conical Kresling origami frusta.^[^ ^210^, ^211^, ^212^ ^]^ Within a planar configuration, each frustum side corresponds to quadrilateral ABCD, intersecting diagonals at point E, with one diagonal acting as a valley crease (refer to Figure 5A(a). Through finite modelling, critical force corresponding to the critical displacement was determined. At the same time, normalized stiffness was assessed across configurations such as twisting collapses and large and small strain conditions relative to twisting and slant angles, respectively (refer to Figure 5A(b,c). It was observed that the pop‐up deformation occurs without panel stretching, and the physical prototype of Kresling frustum did not exhibit any damage after undergoing multiple pop‐up cycles.^[^ ^213^ ^]^ Additionally, the pop‐up frustum exhibits bi‐stable characteristics when the sheet thickness is minimal, transitioning to a mono‐stable state as the sheet thickness increases (refer to Figure 5A(d,e).

Han et al. proposed a quasi‐zero‐stiffness vibration isolators constructed using a Kresling origami module.^[^ ^216^ ^]^ The study reveals that the behavior of the isolator can be influenced by the distinct bifurcation points, including supercritical pitchfork, semi‐subcritical pitchfork, saddle‐node and transcritical, with supercritical pitchfork bifurcation being the key to achieving zero stiffness at equilibrium. The Kresling origami module was designed at the boundary between the monostable and bi‐stable configurations demonstrating the quasi‐zero stiffness properties, meaning stiffness is zero at equilibrium and greater than zero around that equilibrium. Furthermore, the bifurcation diagram confirmed that the Kresling origami module achieves quasi‐zero stiffness at its deployed position in the supercritical pitchfork bifurcation point, while other bifurcation points do not exhibit this property. These findings were further confirmed through the analysis of potential energy, vertical restoring force, and vertical stiffness.

Another modified version of the Kresling tube proposed by Wang et al. is the multi‐triangle cylindrical origami, which introduces an additional mountain diagonal to the Kresling pattern^[^ ^176^, ^217^, ^218^, ^219^ ^]^ that results in tri‐stable states (refer to Figure 5C(a). In terms of deployability, the multi‐triangle cylindrical origami was categorized into four cases: locally deployable bistable, locally deployable tristable, fully deployable bistable, and fully deployable with zero stiffness. The energy landscape of these cylindrical origami structures featured three local extreme points, signifying tri‐stable states (refer to Figure 5C(b). The normalized energy exhibited an extremely low rate of gradual increase, around zero energy, for a considerable duration. The results of this investigation revealed interesting findings that the multi‐triangle cylindrical origami simultaneously possessed zero stiffness and three stable states (refer to Figure 5C(c). In the context of Kresling tubes, Wang et al. further demonstrated tristability in one‐storey cylindrical and conical architectures, where the third stable state is the radially popped‐through stiffened configuration.^[^ ^220^ ^]^ Further insights on multistability and axial‐twist mode coupling of Kresling and waterbomb tubes are presented later in section 3.1.5 with a comparative perspective.

After discussing the constitutive and multistable behavior of Kresling and its modified version, we shift our attention to another compelling tubular origami architecture, Yoshimura‐based tubular metamaterial. Yoshimura architectures are renowned for their exceptional axial stiffness, load‐bearing capacity, and buckling strength.^[^ ^221^, ^222^, ^223^ ^]^ These tubular structures comprise triangular facets and outward‐falling vertices. Suh et al. proposed Yoshimura tubes which can be reconfigured into various configurations, including load‐bearing, hexagonal‐compliant, and rectangular‐compliant. These configurations were achieved by altering the reverse vertices (refer to Figure 5F(a).^[^ ^196^ ^]^ The load‐bearing configuration was the foundation for obtaining hexagonal and rectangular compliant configuration, so transitions between all three configurations go through the load‐bearing configuration. Furthermore, these configurations exhibit multi‐stability, ensuring robustness in each configuration such that when one configuration changes into another configuration by actuation, they do not require additional efforts to maintain their current shape. The experimental and numerical results were obtained for three self‐reconfigurable shape memory polymer‐based tubes: load‐bearing, hexagonal compliant, and rectangular compliant. These results indicate that the load‐bearing configuration possesses significantly higher stiffness relative to the hexagonal and rectangular compliant configurations (refer to Figure 5F(b).

#### 3.1.2 Large‐Scale Shape Morphing

Large‐scale shape morphing involves the transformation of shapes exploiting the mechanics of origami folding. These structures often possess unprecedented properties, including deployability, scalability, flat‐foldability, reconfigurability, and stability at multiple shapes. Deployability implies origami's capability to transit from a compact 2D/3D configuration into their final 3D structure with large volume. It provides easy storage in compact spaces and subsequent deployment into larger structures.^[^ ^224^, ^225^, ^226^, ^227^, ^228^, ^229^ ^]^ Often, origami‐based designs possess flat‐foldability, i.e. the ability to fold an origami model into a flat shape without damaging it or adding new creases. Scalability refers to the ease by which origami structures can be scaled to mili‐, micro‐, and nano‐scale due to their inherent crease pattern,^[^ ^230^, ^231^, ^232^, ^233^, ^234^ ^]^ while the basic mechanics of folding based on the crease pattern remains unaltered. Reconfigurability denotes alteration in origami design to achieve different 3D configurations from a single origami crease pattern without further altering the geometric parameters, and this property is mostly beneficial in the design of multi‐stable structures.^[^ ^89^, ^128^, ^235^, ^237^, ^238^ ^]^ In this context, self‐actuation means the capability of origami to initiate deployment without using an external actuator,^[^ ^239^, ^240^, ^241^, ^242^, ^243^ ^]^ while tunability involves the adjustment of origami's crease or geometric parameters to achieve a specific task.^[^ ^244^, ^245^, ^246^ ^]^

Large‐scale shape morphing structures find extensive applications in various technologically‐demanding fields, such as deployable space structures, robotics, folded core structures, electronics, and biomedical devices. Such large‐scale shape morphing typically occurs through actuation, which can be achieved via magnetic, pneumatic, electric, or thermal means, as discussed later in this paper in more detail. Shape morphing can be classified based on the use of materials into two types: active shape morphing and passive shape morphing. Active shape morphing refers to a structure that can change its shape on‐demand by applying an external stimulus, like electricity or pressure, to actuators embedded within the material, while passive shape morphing describes a structure that changes shape based solely on environmental conditions without requiring any external control, relying on the material's inherent properties to deform in response to stimuli like temperature or humidity. While origami‐based non‐tubular architectures including their derivatives and graded configurations have been exploited to obtain a range of symmetric and asymmetric custom curvatures,^[^ ^119^ ^]^ we will explore a few works related to the large‐scale shape morphing shown by tubular origami metamaterials in this section.

**Figure 6:**
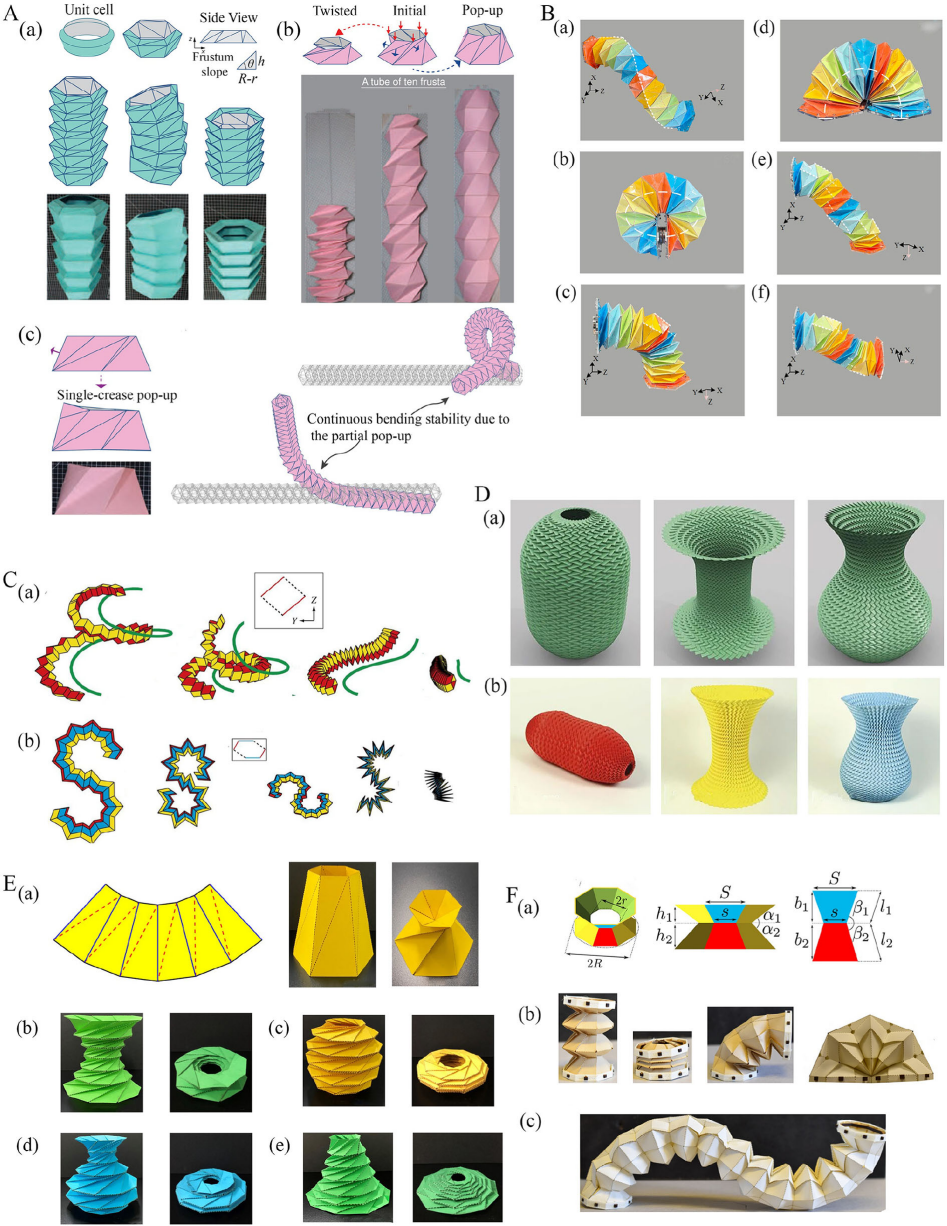
Crease‐architecture driven large‐scale shape morphing. A) Shape modulation of Kresling frustum tubes showing the geometric features along with initial, twisted, and pop‐up states (Reproduced with permission.^[^ ^193^ ^]^ Copyright 2022, Elsevier). B) Folding of tubular Kresling origami at variable angles for crawling robot application (Reproduced under the terms of CC‐BY 4.0 license.^[^ ^176^ ^]^ Copyright 2023, The Authors). C) Origami motion behavior of polygonal tubes showing rigid foldable S‐shaped and spiral tubes in 3D space (Reproduced with permission.^[^ ^21^ ^]^ Copyright 2016, The Royal Society). D) Programming curvatures based on origami tubes with optimal surface geometry (Reproduced with permission.^[^ ^57^ ^]^ Copyright 2016, Springer Nature Limited). E) Large‐scale morphing of conical Kresling tubes showing the target surface attainment in numerical models and physical prototypes (Reproduced with permission.^[^ ^214^ ^]^ Copyright 2022, The Royal Society). F) Polygonal origami robotic behavior showing programmable shape morphing (a) Representation of geometric features (b‐c) Deployment sequence and multi‐stage deformation (Reproduced with permission.^[^ ^215^ ^]^ Copyright 2021, ASME).

Thin‐walled origami tubes are formed by folding thin sheets into 3D tubular structures, which are generally non‐developable.^[^ ^21^ ^]^ Origami tubes with polygonal cross‐sections proposed by Filipov et al.^[^ ^21^ ^]^ can be widely utilised in various domains, such as architecture, robotics, pipelines, and metamaterials, owing to their remarkable mechanical and multi‐physical characteristics. These characteristics include programmable functionalities, including rigid foldability, flat foldability, compatibility, adaptability to various shapes, and enhanced out‐of‐plane stiffness (refer to **Figure** **6**C(a). The proposed rigid foldable S‐shaped cross‐section was achieved by varying the projection angles while preserving the symmetry between the polygonal cross‐section and the projection vector within X‐Y planes. Furthermore, the rigid and flat foldable 3D spiral shape was obtained by projecting a four‐sided origami cross‐section along a spiral in 3D space (refer to Figure 6C(b). These different cross‐sectional shapes obtained in this investigation represented the large‐scale morphing features of thin‐walled origami tubes.

Miura‐ori pattern stands out in engineering applications because of its deployability, foldability, high degree of symmetry, and single degree of freedom. Dudte et al. proposed a Miura‐based programmable origami tessellation which was achieved by fitting the Miura‐ori tessellation onto the surface characterized by intrinsic curvature.^[^ ^57^ ^]^ This investigation proposed a variety of large‐scale morphing shapes, including pills, candlesticks and vases. The pill shape was obtained by adopting the Miura‐ori tessellation on a cylindrical waist featuring positive curvature cap. Similarly, the candlestick shape was obtained by fitting the Miura‐ori origami tessellation on a cylindrical waist with negative curvature cap. The vase shape was achieved by fitting the Miura‐ori origami tessellation on a positive curved base with a negative curvature neck (refer to Figure 6D(a). The efficacy and versatility of programmable optimal shapes were validated through physical prototypes (refer to Figure 6D(b).

Large‐scale shape morphing has been demonstrated in various modified versions of Kresling origami‐based tubular architectures. The conical Kresling origami is a modified version of traditional Kresling origami composed of isosceles trapezoidal unit cells exhibiting bi‐stability (refer to Figure 6E(a). The conical Kresling origami has emerged as a promising option for designing multi‐stable structures offering desirable folded configurations and energy landscapes.^[^ ^214^, ^247^ ^]^ Lu et al. proposed a conical Kresling origami, which is utilised in applications that need target shapes and mechanical responses such as origami antennae and energy‐absorbing origami structures. The desired target shapes for these conical Kresling origami were achieved in two inverse frameworks. The first design approximated arbitrary surfaces of revolution (such as hyperboloid, ellipsoid, and sinusoid) with positive, negative, or mixed Gaussian curvature (refer to Figure 6E(b–d). These approximated designs had arbitrary radii along the vertical axis and exhibited non‐developability. The second design approximated the arbitrary surface of revolution whose cross‐section was monotonic, thus ensuring developability (refer to Figure 6E(e).

Another instance of large‐scale shape morphing is origami‐based bendy straw‐like structures that draw inspiration from drinking bendy straws for developing one‐dimensional metamaterials. The origami‐based bendy straw developed by Bernardes et al. consists of multiple conical frustum formed from isosceles trapezoides (refer to Figure 6F(a).^[^ ^215^ ^]^ These conical frusta were engineered to exhibit axial multi‐stability, allowing the structure to collapse the smallest frustum in its stable compressed state. Furthermore, this origami‐based bendy straw configuration allowed the partial collapse of the smallest frustum, enabling the structure to maintain a bent state (refer to Figure 6F(b). This proposed origami‐based bendy straws were non‐rigid and these can demonstrate stability when in an S‐shape configuration (refer to Figure 6F(c). In this configuration, half of its cells bend in one direction while the remaining half bends in the opposite direction, contributing to the structural integrity of the overall assembly.

One more instance of programmable large‐scale shape morphing is corrugated tubes developed by Wo et al., comprised of Kresling frustums serving as unit cells and these are repeatedly arranged along the longitudinal direction. When the initial configuration of the corrugated tube was shallow and less twisted, the resulting deployment of the corrugated straw demonstrated multi‐stable axial inversion and bending.^[^ ^193^ ^]^ This programmable deployment of a corrugated tube was validated through a paper prototype (refer to Figure 6A(a). A corrugated tube formed from the initial configuration showed a transition into the twisted and pop‐up tubes. With a steeper and more twisted initial configuration, the frustum collapsed into a bi‐stable twisted state, forming a twisted corrugated tube by repeatedly interconnecting the twisted configuration. The application of force to pop up the valley creases of the initial configuration results in a stable pop‐up state. The pop‐up configuration cannot be reversed to the initial configuration by external loading. The pop‐up configuration was repeatedly interconnected in the longitudinal direction to form a pop‐up corrugated tube (refer to Figure 6A(b). A one‐crease pop‐up configuration was achieved when only one valley crease of the initial configuration was raised through loading. A corrugated tube is formed by repeatedly interconnecting the one‐crease pop‐up configuration. This tube maintained stability in a knot shape, and when intermediate units were tilted, they demonstrated a stable L shape (refer to Figure 6A(c). The knot shape and stable L shape achieved by corrugated tubes exemplify the large shape morphing feature of corrugated origami tubes.

An origami‐based robotic arm developed by Wang et al.,^[^ ^176^ ^]^ which draws inspiration from multi‐triangle cylindrical origami, showed multiple stable motions attributed to the unprecedented properties of the multi‐triangle cylindrical origami, including three stable states, the presence of non‐zero terminating stiffness, and enhanced motion characteristics. The programmable twelve‐unit robotic origami arm exhibited free extension motion characterized by the presence of three stable states along the white dash line and the retention of non‐zero terminating stiffness for the remaining unit cells (refer to Figure 6B(a). Moreover, the robotic arm demonstrated stability at bending angles of 360^°^ (refer to Figure 6B(b) and 180^°^ (refer to Figure 6B(d). The stability motion of the robotic arm at a bending angle of 90^°^ could be assessed either when the robotic arm exhibits three stable states for three middle unit cells and non‐zero termination stiffness exhibited by the remaining nine unit cells or when ten unit cells show three stable states in a spiral pattern (refer to Figure 6B(c,e). Additionally, the robotic arm can attain stability at a bending angle of 0^°^ through a twisting motion (refer to Figure 6B(f). The variety of robotic motion capabilities shown by the robotic arm demonstrates the large‐scale morphing of multi‐triangle cylindrical origami.

#### 3.1.3 Energy Absorption

Energy absorbing metamaterials and structures exhibit the capacity to absorb and dissipate kinetic energy upon impact. These structures, owing to the enhancement in crashworthiness, have been extensively utilised in a range of applications, including vehicles, ships, nuclear reactors, crash barriers, aerospace, robotics, and deployable structures. Thin‐walled tubes formed from origami‐based designs utilise the geometric imperfections of origami patterns to reduce peak crushing forces (along with alteration of failure modes) and facilitate collapse in accordance with pre‐defined folded shapes. Unlike traditional foldable origami architectures, the energy absorbing origami tubes have rigid creases and facets. Such origami‐based thin‐walled tubes possess properties such as lightweight design, high energy dissipation capability, and cost‐effectiveness. Energy absorption capacity of thin‐walled tubes can be accessed by three important parameters including, mean crushing force, initial peak force, and specific energy absorption capability. Here, we are going to discuss critically the aspect of energy absorption achieved through origami‐based thin‐walled tubes.^[^ ^191^, ^253^, ^254^, ^255^ ^]^

**Figure 7:**
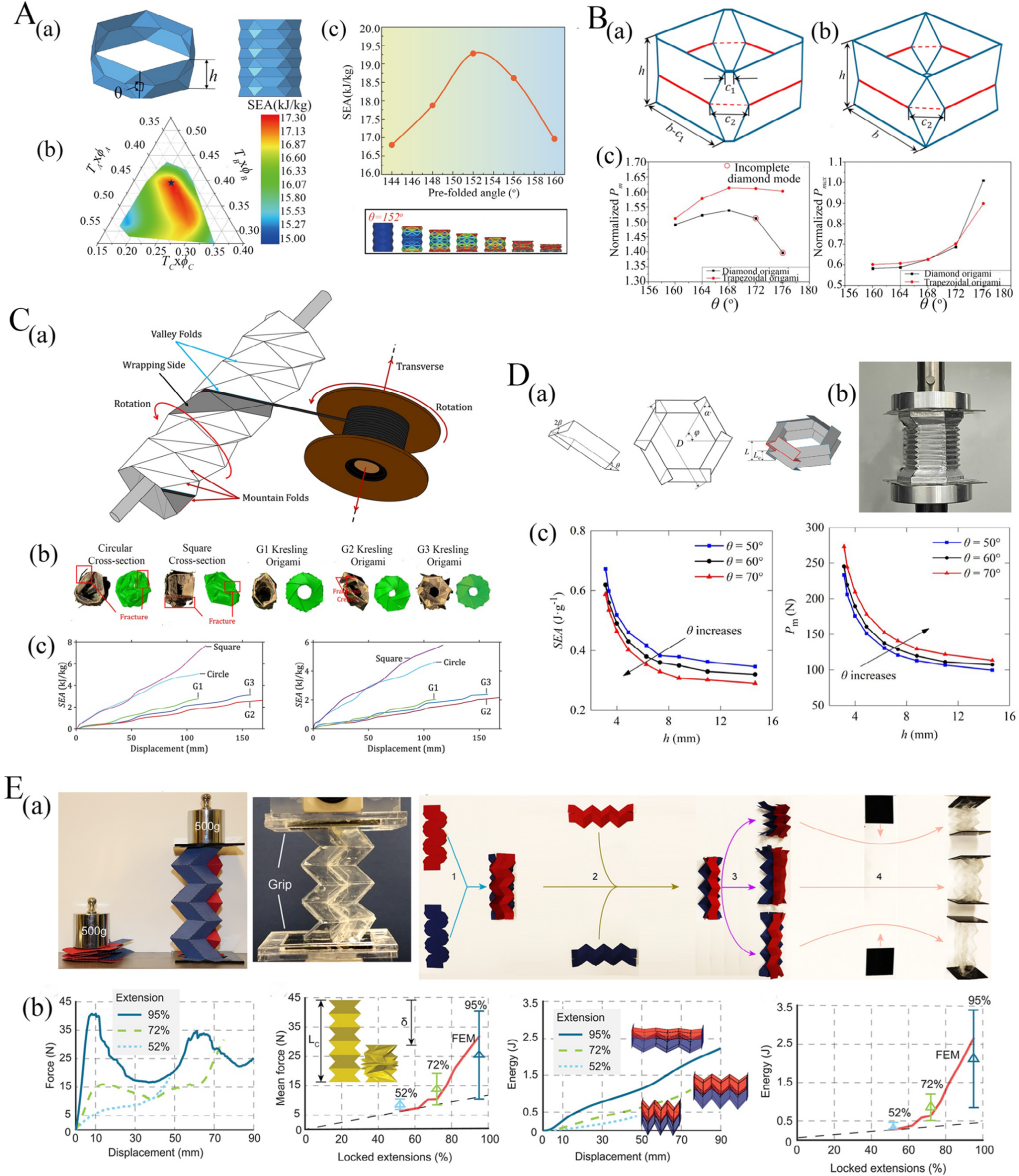
Energy absorption capabilities of tubular metamaterials. A) Pre‐folded non‐uniformizing tube with improved energy absorption capacity (a) Geometric parameters of the thin‐walled tube (b) Contour plot of specific energy absorption (SEA) against optimized thickness at different locations (c) Variation of SEA with folding angle (Reproduced with permission.^[^ ^248^ ^]^ Copyright 2023, Springer Nature). B) Trapezoidal and diamond origami crash‐worthiness design (a) Trapezoidal crash boxes (b) Diamond crash boxes (c) Variation of normalized mean and maximum crushing force with dihedral angle (Reproduced with permission.^[^ ^249^ ^]^ Copyright 2017, Elsevier). C) Pre‐folded filament wounded Kresling tube energy absorption characteristics (a) Schematic representation of the process of filament wounding on Kresling tube (b) Top‐view of various cross‐sections (c) Experimental and Numerical results of specific energy absorption vs displacement for different thin‐walled tubes (Reproduced with permission.^[^ ^250^ ^]^ Copyright 2022, Elsevier). D) Energy absorption characteristics of origami bellows (a‐b) Parametric representation and fabrication of origami bellow (c) Numerical results on the variation of energy absorption with geometric parameters (Reproduced under the terms of the CC‐BY 4.0 license.^[^ ^251^ ^]^ Copyright 2023, The Authors). E) Energy absorption behavior of locking zipper‐coupled origami tube (a) Fabrication procedure of locking zipper‐coupled tubes. (b) Mean loading response and mean absorbed energy curves for different experimental testing and comparison with numerical results (Reproduced with permission.^[^ ^252^ ^]^ Copyright 2022, Elsevier).

Diamond‐shaped pre‐folded thin‐walled tubes proposed by Niu et al. were investigated to analyze the effect of non‐uniformity on the energy absorption performance (refer to **Figure** **7**A(a).^[^ ^248^ ^]^ The thin‐walled tube demonstrated high specific energy absorption, particularly at a folding angle 152^°^ (refer to Figure 7A(c). Furthermore, the energy absorption capacity of pre‐folded thin‐walled tubes was enhanced by categorizing the deformation degree of the tube based on plastic strain into three distinct regions: region A, characterized by high plastic deformation; region B, exhibiting moderate plastic deformation; and region C, showcasing minimal plastic deformation. A contour plot was generated to illustrate the relationship between specific energy absorption and the thickness of regions A, B, and C. This plot highlighted that the peak specific energy absorption value occurred at specific thickness values within regions A, B, and C (refer to Figure 7A(b), leading to the notion of further geometric design accounting for the interplay of thicknesses and other dimensions.

Zhou et al. proposed a trapezoidal origami‐based crash box, which was demonstrated as an excellent energy absorption device. A comparative analysis between the trapezoidal and diamond‐shaped crash boxes was conducted to investigate the optimal design for such crash boxes (refer to Figure 7B(a,b).^[^ ^249^ ^]^ The investigation involved plotting the mean crushing force and maximum crushing force against the folding angle for both types of crash boxes. It was observed that there was not much difference in the maximum crushing force between the two designs. However, the mean crushing force exhibited by the trapezoidal origami crash box was higher than that of the diamond crash box. Furthermore, the difference between the curves depicting mean crushing force escalated with increasing folding angle (refer to Figure 7B(c). Therefore, it can be concluded from this investigation that the trapezoidal origami crash box emerges as the preferred choice for energy absorption compared to the diamond crash box.

Neil et al. proposed a Kresling origami‐based thin‐walled tube.^[^ ^250^ ^]^ Kresling origami structures derive energy absorption capability based on their mechanism to dissipate energy through creases and facets, thereby reducing initial peak force and mean peak force compared to conventional straight, thin‐walled tubes (refer to Figure 7C(a). Neil et al. developed a carbon fibre‐based Kresling origami tube which was fabricated using the filament winding technique, resulting in the creation of three distinct geometries denoted as G1, G2, and G3 Kresling origami tubes. These Kresling tubes were compared with conventional tubes with circle and square cross‐sections manufactured using the same method to investigate the energy absorption ability (refer to Figure 7C(b). The experimental and numerical analyses conducted to evaluate the specific energy absorption performance of all the tubes under consideration showed that Kresling origami tubes do not demonstrate superior energy absorption capabilities over conventional tubes. However, they exhibit characteristics such as high collapse stability and reduced load variation (refer to Figure 7C(c). Furthermore, among the three Kresling tubes, the tubes with smaller heights and greater twists were noted for their enhanced crushing force efficiency. From this study, it becomes evident that different designs should be benchmarked appropriately, along with a comparative performance analysis of other possible alternatives before finalising the architecture.

Zhang et al. investigated the tensile energy absorption performance of origami bellows with varying wall thickness.^[^ ^251^ ^]^ Origami bellow is a thin‐walled tube exhibiting a pleated pattern as depicted in Figure 7D(a,b). The result of the conducted investigation demonstrated the influence of folding angle and unit width on specific energy absorption and mean tensile force. It was observed that an increase in folding angle resulted in decreases in the specific energy absorption and increases in mean tensile force, thereby positioning the origami bellow as a favourable option for applications requiring energy absorption under tensile loading conditions (refer to Figure 7D(c).

Wo et al. proposed a zipper‐coupled tube, formed by repeating and connecting identical Miura‐ori cells.^[^ ^252^ ^]^ Miura ori‐inspired deployable energy absorbing tubes have garnered extensive research attention owing to their capacity to extend the crushing distance and adjust energy absorption capabilities. In this study, the longitudinal configuration of zipper tube was defined by the percentage of extension (i.e., ratio of current length to maximum extended length) rather than dihedral angle. The tube is fully deployed at 100% extension and fully compacted at 0% extension (refer to Figure 7E(a). The experimental results obtained from quasi‐static tests conducted on polyester zipper tube locked at three distinct state of extensions (52%, 72%, and 95%), revealing that tube with higher extension (95%) demonstrate high axial stiffness, high peak force, and enhanced energy absorption capacities over the same crushing distance. Furthermore, the comparative analysis between computational and experimental results, presented in terms of mean reaction force and absorbed energy, showed that the deploying and locking of zipper tube at different extension can be used to increases the reaction forces and the total amount of energy absorbed (refer to Figure 7E(b).

Yang et al. proposed new designs by assembling multiple Kresling tubes in a honeycomb‐like cellular configuration (each tube acts like the unit cell that is tessellated in two directions to form a periodic honeycomb lattice) to achieve optimized mechanical energy absorption capabilities.^[^ ^261^, ^262^ ^]^ The Kresling unit was chosen in the study because it exhibits distinct deformation modes under different boundary conditions including twist free deformation (elastic deformation concentrated at creases) and twist limited deformation (elastic deformation concentrated in facets). The study revealed that energy absorption capabilities of the Kresling units can be adjusted by changing boundary conditions and the connection mechanism among the tubular origami units. The results based on finite element analysis and experimental investigation demonstrated that the 2D Kresling honeycomb arrays can exhibit significantly enhanced specific energy absorption, crushing force efficiency, mean compressive force, and peak compressive force when compared to a traditional honeycomb array. The study investigated three different Kresling metamaterial configurations including tiered array, hourglass array, and fusiform array, among which fusiform array at 30^°^ orientation provided superior mechanical properties.

In general, the investigations on origami‐based tubular metamaterials for energy absorption reveal that the crease architectures should be chosen based on a number of considerations, including tensile and compressive modes of loading, strain rate and failure mode. Another aspect concerning energy absorption that needs significant attention is the realistic situation of having combined loading scenarios, including axial, bending, shearing and twisting modes. Further, there exists tremendous scope of multi‐objective design optimisation under different loading scenarios considering the crease architectures, different patterns, their geometries and intrinsic materials. The aspect of gradation to deal with application‐specific strain rates is largely unexplored, which holds significant potential for increasing specific energy absorption capability.

#### 3.1.4 Programmable Dynamic Characteristics

Dynamic analysis of tubular metamaterials is performed to analyze the mechanical behavior of tubular metamaterials under dynamic loading and further programming the crease architectures to achieve target behavior. Dynamic analyses ensure structural integrity, deployment and folding mechanism, optimization of performance, and enhancing stability across a wide range of applications. Origami plays a crucial role in vibration attenuation across various frequencies. The target attenuation of the signal at the specific frequencies can be achieved by passing the signal through architected origami folds, which results in resonance behavior and causes the signal to be absorbed and reflected for effective diminishing of signal strength. Through the research on folding mechanics and deployment is predominant in the field of origami, the dynamic behavior of such structures is increasingly getting attention as we briefly discuss in this section.

Zhou et al. proposed a stacked miura origami structure which is bi‐stable and exhibits two types of dynamic behavior under harmonic base excitation, including small intrawell and large interwell amplitude oscillation. The transmissibility analysis conducted on these stacked Miura origami suggested that at some frequency intervals, the intrawell oscillation attenuates the excitation, and the interwell oscillation amplifies the excitation.^[^ ^263^, ^264^ ^]^

**Figure 8:**
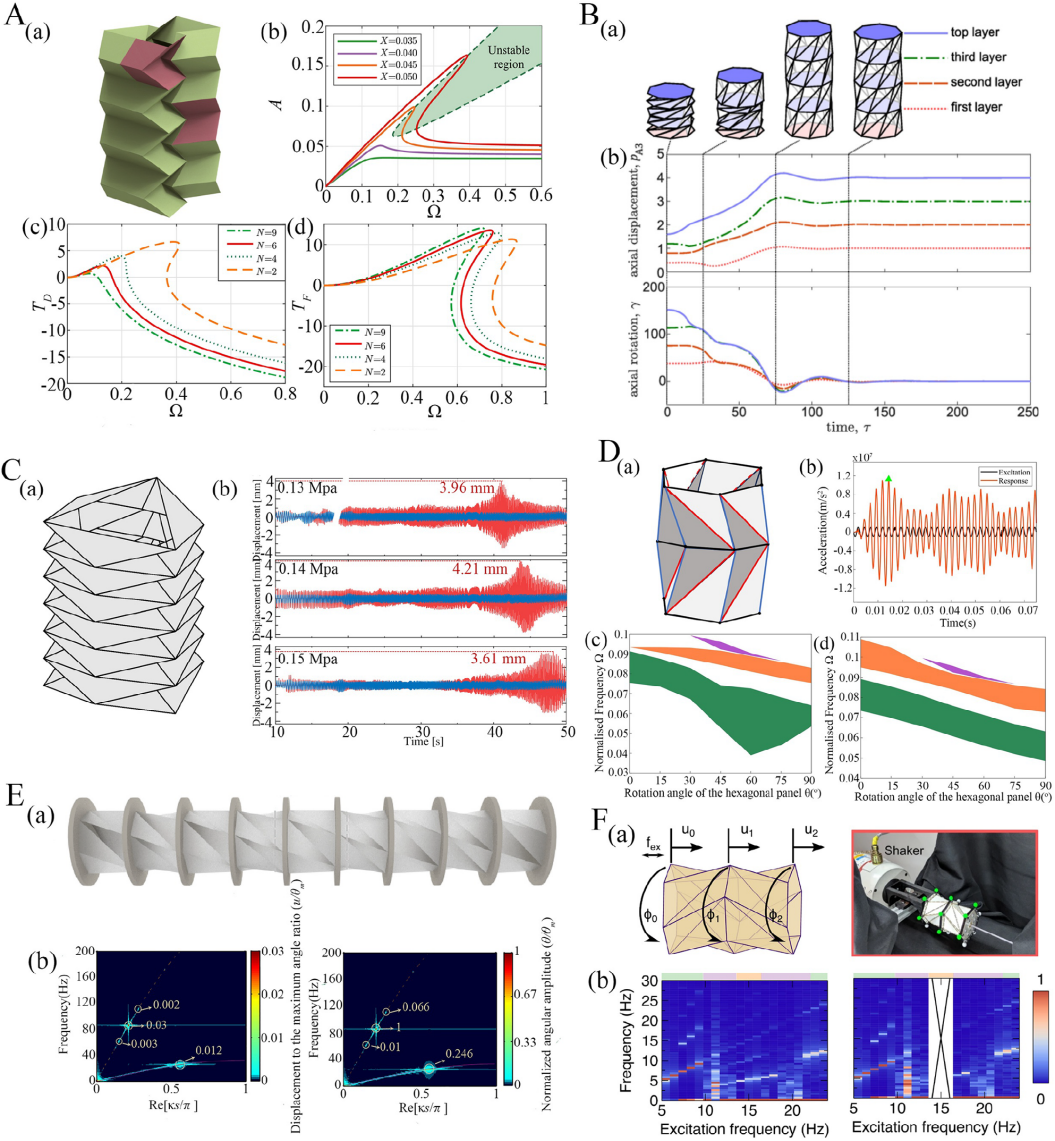
Dynamics of tubular origami metamaterial. A) Tachi Miura origami tubes for vibration isolation (a) Geometric configuration of Tachi Miura origami (b) Amplitude vs frequency response for variable excitation (c) Displacement transmissibility curve (d) Force transmissibility curve (Reproduced with permission.^[^ ^256^ ^]^ Copyright 2021, Elsevier). B) Dynamic analysis of tubular Kresling origami (a) Deployment stages of Kresling origami (b) Axial displacement, axial rotation vs time curve showing the dynamic deployment behavior (Reproduced with permission.^[^ ^257^ ^]^ Copyright 2020, American Physical Society). C) Tunable dynamic analysis of Yoshimura origami tubes under pneumatic actuation (a) Yoshimura origami architecture (b) Sweep test results showing displacement vs time curve at different inflatable pressure (Reproduced with permission.^[^ ^127^ ^]^ Copyright 2022, Elsevier). D) Dynamic analysis of Kresling origami metamaterials for vibration isolation (a) Kresling origami cylinder geometry (b) Acceleration vs time curve for excitation and structural response (c) Longitudinal band gap response for variable rotational angle θ (d) Transverse band gap response for variable rotational angle θ (Reproduced with permission.^[^ ^258^ ^]^ Copyright 2023, Elsevier). E) Non‐linear dynamic analysis of Kresling origami based metamaterials (a) Kresling origami‐based metamaterial architecture (b) Numerical results of displacement to maximum angular ratio and angular amplitude for different frequencies (Reproduced with permission.^[^ ^259^ ^]^ Copyright 2024, Elsevier). F) Dynamic mode analysis of Kresling origami (a) Schematic diagram of Kresling origami and vibration testing setup (b) Spectrum analysis of experimental and predicted axial displacement for the first panel (Reproduced with permission.^[^ ^260^ ^]^ Copyright 2023, Elsevier).

Liu et al. conducted a frequency domain analysis on the dynamic behavior of Tachi‐Miura‐based vibration isolator.^[^ ^256^, ^266^ ^]^ The Tachi‐Miura tubular origami configuration comprises multiple identical Miura‐ori^[^ ^267^, ^268^, ^269^ ^]^ unit cells arranged in series in the vertical direction (refer to **Figure** **8**A(a). The frequency domain analysis suggested that the curve exhibited a rightward bending tendency due to hardening stiffness, and as the excitation frequency increased, the bending trend became stronger. Furthermore, when the response curve reaches a specific degree of curvature, a jump phenomenon exists, and at that point, two frequencies with higher excitation are inherently unstable. As the excitation frequency increases, concurrent system amplitude decreases, ultimately converging toward a stable equilibrium (refer to Figure 8A(b). Additionally, varying the geometric parameter (*N*) shows dissimilar responses in displacement transmissibility (*T*_*D*_), characterised by a relatively mild non‐linear response, contrasted with force transmissibility (*T*_*F*_), which shows an apparent non‐linear response concerning frequency ratios (refer to Figure 8A(c,d).

Kidambi et al. conducted a deployment analysis on Kresling tubes. Tubular structures inspired by Kresling's principles exhibit multi‐stability and adjustable stiffness characteristics provided by manipulating geometric parameters.^[^ ^257^ ^]^ The deployment analysis of the four‐layer Kresling^[^ ^270^, ^271^ ^]^ tube involved base fixing, and initial compression to 40 % of original height followed by releasing the compressed Kresling tube. The structural behavior was observed at its initial, intermediate, and final stages, wherein the results suggested that the deployment process did not progress sequentially through intermediate states due to pronounced geometric non‐linearity and reaction forces influenced by fixed boundary conditions.^[^ ^272^ ^]^ Additionally, the final stage of deployment showed a slight overshoot, a phenomenon reasonably attributable to the selection of the damping ratio (refer to Figure 8(B(a‐b)). A recent study has introduced conicity in the tubular Kresling architecture, leading to rich nonlinear dynamics with programmable features in an expanded design space.^[^ ^247^ ^]^

Yu et al. investigated a non‐linear tension‐torsion coupling motion within Kresling origami structures^[^ ^259^, ^273^ ^]^ to explore unconventional wave phenomena occurring during large deformations. The metamaterial was fabricated using a sequence of bi‐stable state Kresling origami units arranged with circular plates (refer to Figure 8E(a). Each mass block within the system went through combined translational and rotational motion upon excitation. The numerical analysis of displacement and rotation angle concerning wave propagation was conducted through 2D Fourier transformation (refer to Figure 8E(b,c). The findings of this investigation indicated the consistency in displacement and rotation angle amplitude ratios across various frequency components within their respective modes.

Li et al. investigated the intricate dynamic motion of a dual origami‐based structure when subjected to excitation.^[^ ^260^ ^]^ This dual system comprises bistable Kresling origami elements exhibiting opposite chirality, denoted by variables *u*_*o*_, ϕ_*o*_, *u*_1_, ϕ_1_ and *u*_2_, ϕ_2_, representing axial and rotational displacements at the first, second, and third separator levels (refer to Figure 8F(a). The spectral analysis of frequencies obtained through Fourier transformation of experimental data revealed that responses along the diagonal direction of each panel exhibit the same frequency as the excitation frequency. Furthermore, periodic and chaotic motion can be distinguished by observing an increase in lower frequencies. The predictive analysis indicated that in interwell periodic motion, a large difference in axial and rotational modes exists across the origami separator (refer to Figure 8E(b).

Zhang et al. conducted a dynamic analysis on pneumatic actuator‐based Yoshimura tubular structures.^[^ ^127^ ^]^ The Yoshimura structure, known for its cylindrical form, hollow interior, and exceptional deformability, presented an ideal platform for achieving tunable origami dynamics by incorporating the pneumatic actuator inside the Yoshimura^[^ ^165^, ^274^ ^]^ hollow interior (refer to Figure 8C(a).^[^ ^127^, ^275^ ^]^ The results of the dynamic sweep test showed that for the increase in pressure value to 0.14 MPa, an increase is observed in the maximum output displacement value to 4.21 mm. However, this trend was not uniformly sustained at a pressure of 0.15 MPa, where the output value diminishes due to the influence of shaker power (refer to Figure 8C(b). Conclusively, the dynamic experimentation confirmed the capacity to modulate pneumatic Yoshimura cells through pressure inflation, thereby strengthening the adaptability and versatility of these origami tubes.

Liu et al. conducted an experimental investigation on a metamaterial assembly employing Kresling origami‐based compression twist composed of beams and plates with the aim of analyzing its vibrational behavior (refer to Figure 8D(a).^[^ ^258^, ^276^, ^277^, ^278^ ^]^ The results of the structural response in the time domain revealed that at a specific frequency, the excitation acceleration passed through response acceleration, resulting in a maximum amplification of the vibration response by a factor of 11.3 (refer to Figure 8D(b). Moreover, it was observed that the central frequencies of six distinct bandgaps in both longitudinal and transverse orientations decrease as the initial angle difference (θ) increases (refer to Figure 8D(c,d). Therefore, the results concluded that the structure tends to decrease performance in frequency isolation as the initial angle difference increases.

#### 3.1.5 Programmable Axial‐Twisting Mode Coupling, Poynting Effect and Energy Landscape

**Figure 9:**
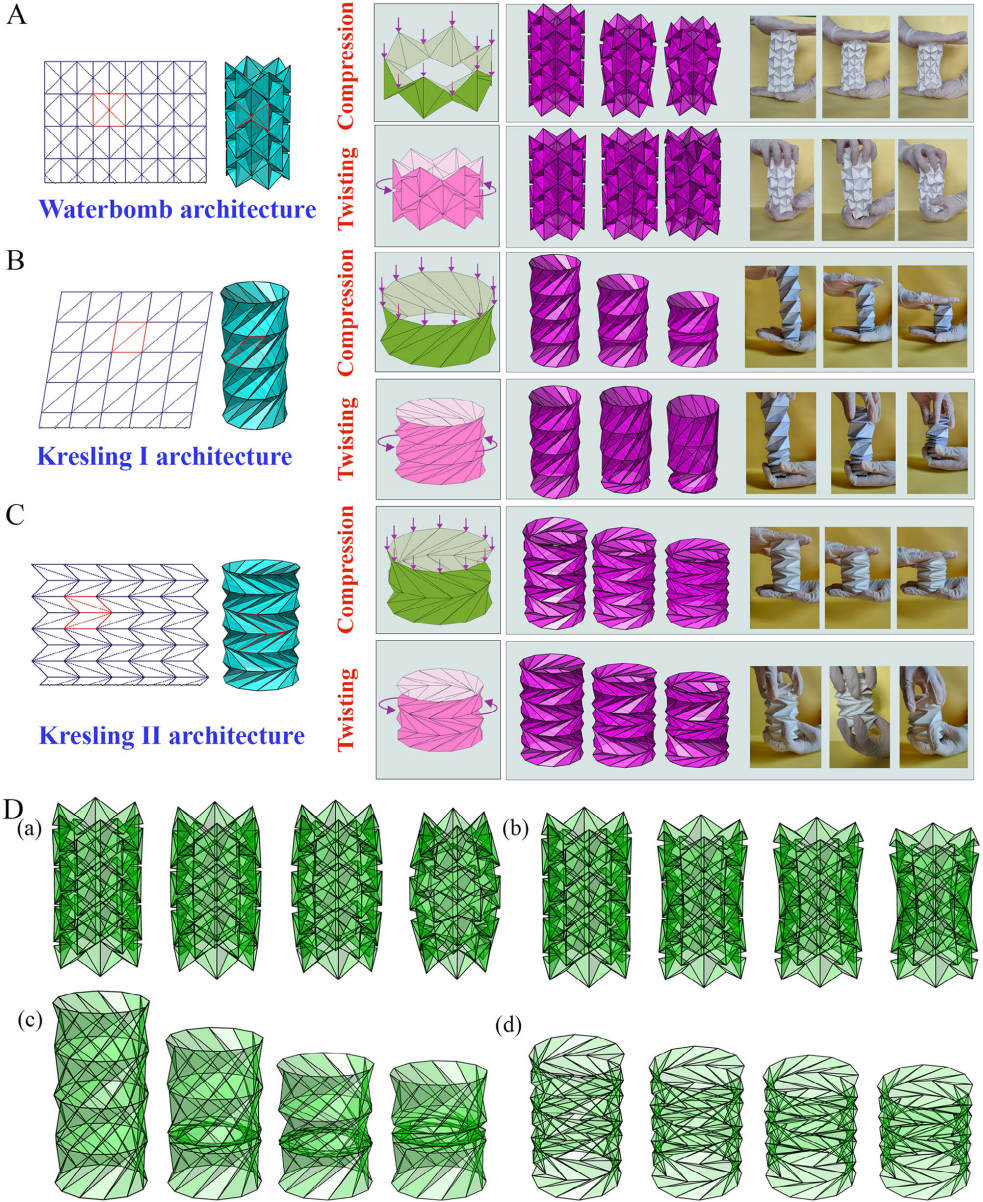
Crease‐architecture driven deformation‐mode coupling and programmable multi‐stability. A–C) Crease architecture and the resulting tubular model of waterbomb, Kresling I and Kresling II origami, wherein the deformation behavior under axial compression and twisting moment is demonstrated based on numerical models and experimental prototypes^[^ ^265^ ^]^ D) (a, b) Spatial shape modulation of waterbomb tubes with resulting convex and concave curvatures (c, d) Large‐scale axial deformation of Kresling I and Kresling II tubes where the former exhibits multistability.^[^ ^124^, ^265^ ^]^

The intriguing aspect of deformation mode coupling between the axial and twisting modes in different classes of origami tubes have not been explored adequately, barring a few literature.^[^ ^265^ ^]^ In this section, we present exploitable insights (based on computational and experimental results) on the coupling behavior of three different tubular architectures (refer to **Figure** **9**A–C) with a comparative understanding of their capabilities. Such tubular architectures are often made using triangualted crease patterns, but distinct orientation of crease geometry can lead to programmable mechanical behavior.^[^ ^265^ ^]^ We focus on exploring whether there can be twisting or axial deformation under the application of axial or twisting far‐field forces (i.e. at the two ends of the tube) in a compulsory or discretionary way as a function of the crease architecture. The aspect of monostability and multistability is also investigated in this context for tubular origami metamaterials (refer to Figure 9A–C) as a function of the crease architecture.

The current discussion centers around three major tubular metamaterial architectures with tri‐angulated crease pattern: waterbomb, Kresling I, and Kresling II (refer to Figure 9A–C). These structures may come across diverse loading conditions in wide‐ranging applications, which may impact their structural performance. Further, there exists a huge scope of deformation mode conversion including wave steering by exploiting axial‐twist coupling in a range of programmable metamaterials. To begin the discussion, we first describe the architecture of these three tubular origami metamaterials under consideration. The waterbomb base comprises a square sheet having six creases: two creases parallel to the vertical sides of the square represent the mountain crease, and four diagonal creases represent the valley creases. The Kresling I base comprises a parallelogram sheet with five creases: four sides of the parallelogram represent mountain creases, and one diagonal represents the valley crease. The Kresling II base comprises two identical parallelograms with mirror configurations connected to a common side. These parallelograms are arranged in series on the vertical axis, with nine creases: seven sides of the parallelogram represent mountain creases, and two diagonals represent valley creases. These unit cells are repeatedly arranged in series along vertical and circumferential directions to obtain a tubular configuration of waterbomb, Kresling I, and Kresling II (sometimes one row in the vertical direction is referred to as a storey). Note that the three origami architectures under consideration here are referred to as triangulated origami architecture I, II and III in the literature to emphasize on their common triangulated crease pattern.^[^ ^265^ ^]^

The characteristic mechanical behavior of the three different tubular architectures, can be noted as follows (refer to Figure 9 along with corresponding supplementary videos SM1 to SM3):^[^ ^265^ ^]^

- *Waterbomb Tubes*: 1) Under axial force it shows only axial deformation (this is true up to a threshold limit of compressive force, beyond which there will be both twist and axial deformation ‐ this aspect is discussed as critical remarks below). Thus under low value of compressive force, no axial and twist coupling exists. 2) Under the application of twisting moments, there will be both twisting deformation and axial deformation simultaneously. Thus, there exists a axial‐twist coupling under the application of twisting moment. 3) The waterbomb tubes always show a mono‐stable behavior. 4) There exists a significant lateral deformation under the application of axial and twisting forces, leading to a programmable spatially‐varying bulging along the length of tubes.^[^ ^124^ ^]^
- *Kresling I Tubes*: 1) Under axial force it shows both axial deformation and twisting simultaneously. 2) Under the application of twisting moments, there will be both twisting deformation and axial deformation simultaneously. Thus, there exists a axial‐twist coupling under the application of both axial force and twisting moment. 3) The Kresling I tubes may exhibit mono‐stable, bi‐stable and multi‐stable behavior depending on the crease architecture. 4) The lateral deformation under the application of axial force or twisting moment is less significant compared to the waterbomb tubes.
- *Kresling II Tubes*: 1) Under axial force it shows predominantly axial deformation. 2) Under the application of twisting moments, there will be predominantly twisting deformation. Thus, there exists no (or less significant) axial‐twist coupling under the application of both axial force and twisting moment. 3) The Kresling II tubes may exhibit mono‐stable, bi‐stable and multi‐stable behavior depending on the crease architecture. 4) The lateral deformation under the application of axial force or twisting moment is less significant compared to the waterbomb tubes.

In the context of the above discussion, we provide supplementary movies (SM1 to SM3) showing the axial‐twist coupling of the three origami patterns (for further detail and comparison with physical model, refer to Sharma et al.^[^ ^265^ ^]^). In SM1, we demonstrate the waterbomb tubes show monostability under both axial load and twisting moment. The aspect of post‐contraction twist (as discussed below) is also presented. In SM2 and SM3, we demonstrate that the Kresling I and II configurations can have both monostable and bi‐stable (or multi‐stable behavior for tubes with multiple storeys) configurations under axial load and twisting moment, depending on the crease architecture.

*Critical Remarks on Post‐Contraction Twist*: In the above discussions concerning the deformation process of waterbomb tubes we have highlighted that a predominantly axial mode of deformation is exhibited under the application of axial loads. However, this is not valid for a very high amount of applied axial force and a post‐contraction twisting deformation can be observed beyond a critical value of axial force. During this stage of deformation, the twist motion starts in the middle of the tube and successively spreads toward both ends.^[^ ^159^ ^]^ Note that as a result of this twisting deformation, there exists axial deformation as well during the second phase of the deformation. In other words, the predominant deformation modes (and axial‐twist mode coupling behavior) of waterbomb tubes are dependent on the stage of deformation under the application of axial force. 1) There exists only predominant axial contraction in the first stage (covered in the preceding paragraphs) 2) Beyond a critical compressive load value, in the post‐contraction second stage, there exists both axial and twisting deformations predominantly with a strong axial‐twist mode coupling. This post‐contraction twist behavior is demonstrated in literature based on the reduced‐order bar‐hinge based modelling approach.^[^ ^265^ ^]^

*Critical Remarks on Poynting Effect*: The deformation mode coupling behavior of tubular origami metamaterials, as presented in the preceding paragraphs, shows a numerical quantification of the extent of coupling between axial and twisting deformations under applied axial force or twisting moment. This behavior can be explained in terms of Poynting and inverted Poynting effect.^[^ ^279^ ^]^ The Poynting effect is a non‐linear elastic effect in which a cylinder expands axially under the application of torsion (or applied shear stress). A positive Poynting modulus causes shear‐induced (or torsion‐induced) normal expansion, while a negative Poynting modulus leads to normal contraction. When, on the contrary, an applied pure axial compression results in twisting (or shear) deformation, it is referred to as inverted Poynting effect. Generally, the Poynting effect adheres to the Maxwell‐Betti reciprocity theorem, which demonstrates that identical shear displacements in the opposite directions result in equal normal stresses. However, in some lattice‐based tubular metamaterials, this theorem is violated, leading to a phenomenon known as the non‐reciprocal Poynting effect, where identical shear displacements in opposite directions induces unequal normal stresses.^[^ ^280^ ^]^ In the current context, we deal with cylindrical tubes, where the Poynting and inverted Poynting effects are defined based on the coupling between axial and twisting modes. A programmable Poynting effect can be achieved in origami tubes as a function of crease architecture.^[^ ^265^ ^]^ If we only consider the predominant deformation modes of the three tubular origami architectures, the following inferences can be drawn. 1) Waterbomb tubes exhibit Poynting effect throughout the entire deformation under the application of twisting moment, but the inverse Poynting effect is only exhibited during the post‐contraction deformation stage under the application of axial force. Thus, the exhibited inverse Poynting effect here is dependent on the motion stage. 2) Kresling I architecture exhibits both Poynting effect and inverse Poynting effect throughout the deformation process. 3) The Poynting effect and inverse Poynting effect are negligible in the case of Kresling II tubes.

#### 3.1.6 Robotic Origami Metamaterials

Origami‐inspired designs have emerged as promising solutions in the field of soft robotics with enhanced efficiency, simplifying the system based on the mechanics of architected creases. Origami robots can modify their shape according to diverse working environments and specific tasks owing to their properties, such as geometric parameters, crease alignments and actuation (as discussed later in the paper) capabilities. Moreover, origami robots can be fabricated from a wide range of materials, including metals, papers, composites, and shape memory alloys/polymers, across scales ranging from milli to nano. These origami robots show multifunctional capabilities such as crawling, swimming, jumping, and grasping, where tubular origami has been widely used due to their exceptional mechanical shape‐morphing characteristics in a programmed and controlled framework. In this section, we will demonstrate the capabilities of origami‐based soft robots, considering a few critical instances of tubular architectures that essentially couple the large‐scale programmed shape‐morphing features (refer to Section 3.1.2) and multi‐physical actuation to dynamically control the motion (refer to Section 3.2).

**Figure 10:**
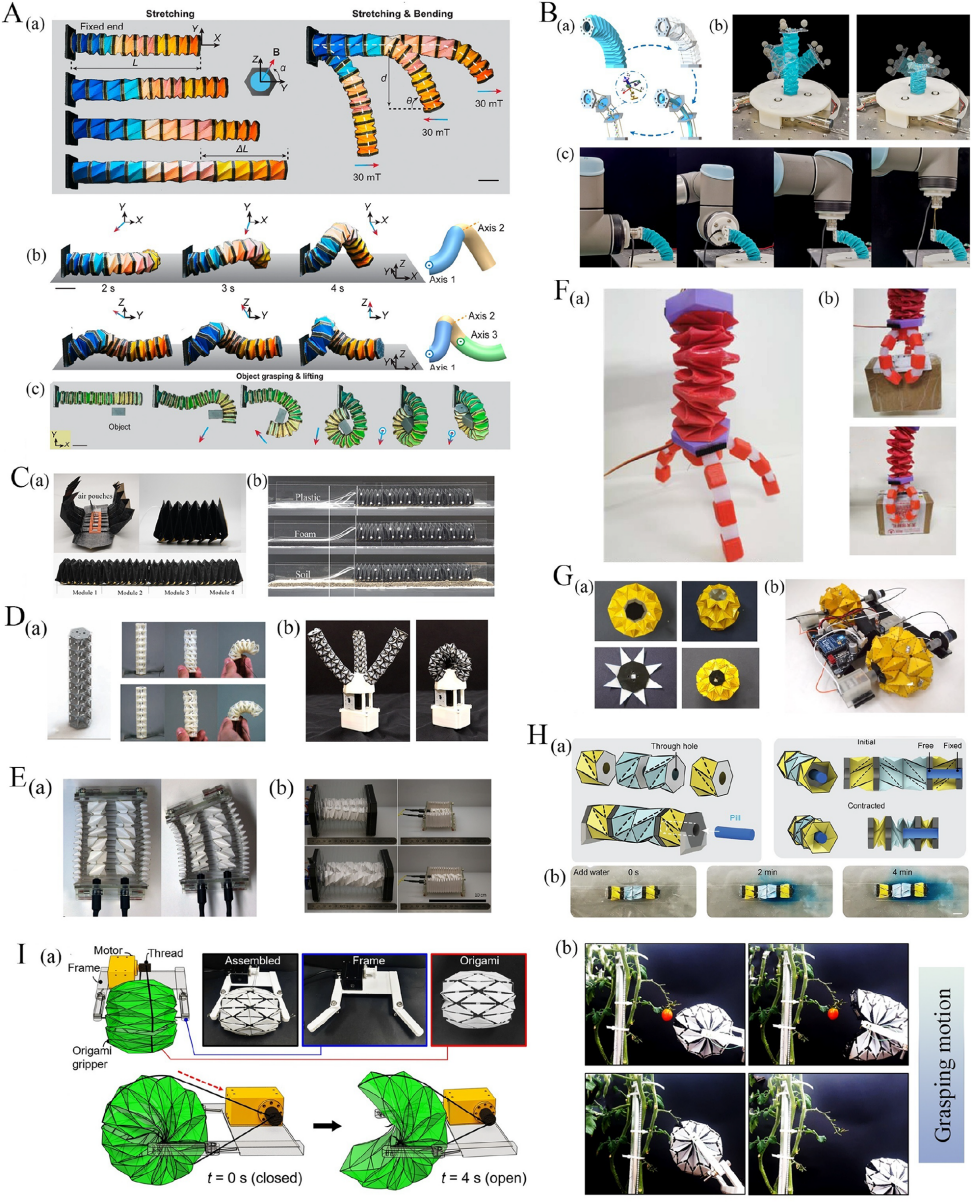
Tubular origami‐based robotic metamaterials. A) Kresling‐based robotic arm for biomimetic motion (a‐b) Octopus‐like robotic arm with stretching, bending, and multi‐axis twisting behavior (c) Object grasping and lifting motion (Reproduced under the terms of the CC‐BY 4.0 license.^[^ ^281^ ^]^ Copyright 2021, The Authors). B) Hybrid continuum origami robots (a) Deformation mechanism approximation (b‐c) Deformation behavior of hybrid robot at variable stiffness (Reproduced with permission.^[^ ^282^ ^]^ Copyright 2021, Elsevier). C) A textile origami snake robot showing rectilinear locomotion (Reproduced with permission.^[^ ^283^ ^]^ Copyright 2023, Elsevier). D) Under‐actuated robotic origami gripper showing 3D printed twister (Reproduced under the terms of the CC‐BY 4.0 license.^[^ ^284^ ^]^ Copyright 2021, The Authors). E) Multistable origami inspired crawling robot (a) Top‐view of crawling robot (b) Contraction and expansion based crawling motion (Reproduced with permission.^[^ ^285^ ^]^ Copyright 2017, IOP Publishing). F) Programmable robot based on origami spring, wherein we show a soft robotic arm with three attached arms and its grasping motion (Reproduced with permission.^[^ ^286^ ^]^ Copyright 2020, Sage Publication). G) Deformable wheel robot driven by origami structure showing different parts and its assembled view (Reproduced with permission.^[^ ^287^ ^]^ Copyright 2013, ASME). H) Kresling origami based soft mini crawling robot. The application of drug storage and release is demonstrated (Reproduced with permission.^[^ ^288^ ^]^ Copyright 2022, The Authors, some rights reserved; exclusive licensee). I) 3D printed origami soft gripper showing the assembled view and grasping motion (Reproduced under the terms of the CC‐BY 4.0 license.^[^ ^289^ ^]^ Copyright 2023, The Authors, Licensee MDPI).

Wu et al. developed a Kresling origami‐based bio‐inspired robotic arm which is engineered to mimic the versatile motions of an octopus arm, including walking, swimming, and capturing prey.^[^ ^281^ ^]^ The untethered robotic arm actuated by magnetic actuation exhibits controlled forward as well as backward stretching and bending motions (refer to **Figure** **10**A(a,b). Furthermore, the robotic arm also mimics the curled characteristic of an octopus arm, providing functions such as grasping and lifting objects (refer to Figure 10A(c).

Zhang et al. developed an origami‐inspired continuum, tendon robot, which has garnered considerable attention owing to seamless motion capabilities and lightweight structure.^[^ ^282^ ^]^ The continuum tendon robot is comprised of a soft structure that holds the internal framework and exhibits a high extension ratio (refer to Figure 10B. The motion and stiffness of the continuum robot were regulated by pneumatic actuators (refer to the following section for a detailed discussion on actuation). The gripper mechanism was formed by interconnecting various continuum tendons on a rigid surface, facilitating adaptable and controlled grasping motions.

Luo et al. proposed snake‐like robots which act as valuable and distinctive mobility platforms for search and rescue operations.^[^ ^290^ ^]^ Origami‐based snake robots mimic the lateral undulation and sidewinding locomotion observed in snakes. The origami snake is constructed from four origami modules, which are connected sequentially. These robots draw inspiration from the Yoshimura pattern and are fabricated from folded plastic material. Each module is equipped with a controller, providing smooth repair and modification in case of any defects. The locomotive motion of the origami is governed by an electric actuator, rendering it a versatile, lightweight, and economically viable robotic solution.

Seyidoglu et al. developed a modular textile origami snake robot to mimic snake‐like rectilinear locomotion, demonstrating potential for applications in a range of areas.^[^ ^283^ ^]^ The design features bio‐inspired fabric‐based origami modules which integrate pneumatic actuation to create movement through air pressure, along with friction modulation to improve the robot's grip surfaces for smoother locomotion. The combination of textile material and origami serves a dual role, functioning as both robotic skin and artificial muscles (refer to Figure 10C.

Lee et al. developed an origami‐based gripper which comprised three twisted towers.^[^ ^284^ ^]^ Each twisted tower was embodied with an origami‐based continuum‐compliant mechanism (refer to Figure 10D). This origami design was converted into a CAD model and subsequently 3D printed to produce the twisted tower structure. The motion of the gripper for adaptable grasping of objects of different shapes, sizes, weights, and textures was governed by an electric actuator.

Pagano et al. proposed a Kresling origami‐based crawling robot, which utilises the origami principles to mimic crawling locomotion and lateral motion (refer to Figure 10E.^[^ ^285^ ^]^ The robot consists of two Kresling origami towers incorporated by a protective folded bellow, which allows the rotation of moving components. The folding bellow was attached to a 3D‐printed plate which was further connected to an electric motor. The alternate expansion and contraction of towers drive the crawling motion of origami robots.

Hu et al. developed a fully programmable robot inspired by origami springs. The robot was formed using an origami spring model that exhibits dynamic geometry and enables a transition from transverse compression to longitudinal stretchability and curvilinear deployment (refer to Figure 10F).^[^ ^286^ ^]^ The soft robotic arm includes a 3D‐printed origami spring that was further integrated with a finger gripper. The electric actuator controls the contraction and elongation motion of the robotic arm, facilitating the grasping motion.

Lee et al. proposed a deformable wheel robot which was constructed using ball‐shaped waterbomb patterns, owing to its transformative properties from a long cylindrical tube to a circular tube.^[^ ^287^ ^]^ The magic ball was fabricated using polyimide film, and a shape memory alloy actuator was incorporated to regulate wheel deformation, driving, and steering motions (refer to Figure 10G. This innovative design enables the robot to move in forward, backward, and lateral directions. Furthermore, the manipulation of the wheel's deformation allows the robot to navigate through slits narrower than its wheel diameter.

Ze et al. developed a Kresling origami‐inspired soft crawler drawing inspiration from the locomotion patterns of earthworms.^[^ ^288^ ^]^ The assembly comprised of two Kresling dipoles exhibiting dual‐levels of symmetry. This small‐scale, untethered crawler possesses both crawling and steering capabilities governed by a magnetic actuator. The soft crawler robot can navigate through confined spaces due to anisotropic and magnetic tunable stiffness along the axial and lateral directions. It can have interesting applications in drug delivery, where this soft crawler's inner cavity stores the drug pill attached to the magnetic plates. The magnetic actuator can facilitate the contraction of the crawler and push the pill to the adjacent unit, resulting in the release of the pill at the desired target point (refer to Figure 10H.

Choi et al. proposed a soft origami gripper which was structured according to the Yoshimura pattern for its adaptable nature and simple configuration (refer to Figure 10I(a).^[^ ^289^ ^]^ The Yoshimura pattern can easily be modified by altering the geometric parameters. The origami grasper body was fabricated using shape memory alloy and the thermo‐electric actuation was used to govern the grasping motion of the origami gripper. This soft gripper is capable of grasping delicate objects, making it applicable in domains such as agriculture, space and other engineering fields (refer to Figure 10I(b).

Wu et al. proposed a Kresling origami‐inspired soft modular robot based on the locomotion of a caterpillar. This soft modular robot can demonstrate bidirectional and steering motion actuated by an electro‐thermal actuator. The origami robot consists of multiple Kresling units, and each alternative Kresling unit was incorporated by the electrothermal actuator to control the desired deformation.^[^ ^150^ ^]^

### 3.2 Active Multi‐Physical Programmability Through on‐Demand Actuation and Control

Tubular origami metamaterials and metastructures can be actively controlled (on‐demand, post‐manufacturing) for a range of advanced engineering and biomedical applications by introducing active or passive actuation mechanisms in the origami architectures. This exploitable nexus of folding mechanics, crease architectures and multi‐physical external stimuli can lead to a range of exotic mechanical properties, from active stiffness modulation to robotic motion and shape morphing without the need for intricate placement of local actuation mechanisms. Active origami structures utilize active materials to convert diverse forms of external energy into mechanical energy for realising origami motion. In other words, active origami enables the folding and unfolding of the structure without necessitating direct external mechanical load but utilises external stimuli (pneumatic, magnetic, electrostatic, and thermal). This section will focus on the predominant actuation approaches in the field of tubular origami for achieving a diverse range of functionalities.

#### 3.2.1 Magnetic Actuation of Tubular Origami Metamaterials

Magnetic control‐driven actuators have been extensively explored across mili‐, micro‐, and nano‐scales. Magnetic actuators possess the capability to perform multiple motions (on‐demand and contactless) through the manipulation of magnetic field intensity and direction. Furthermore, magnetic actuators provide the capability for shape control via untethered, ultrafast, and precisely controlled actuation speeds, along with the potential for distributed actuation. Magnetic actuation can be realized by incorporating an internal permanent magnet within the structure of an origami robot, and subsequently activating it through an external permanent magnet positioned variably relative to the internal permanent magnet. This magnet configuration induces torques and forces within the robot to induce motion.^[^ ^295^, ^296^, ^297^, ^298^ ^]^ In addition, a magnetic actuator can be constructed by employing magnetic soft materials such as iron, nickel, and alloys. These magnetic soft materials are either embedded within or coated onto the origami sheets or structures. These resulting magnetic origami structures are activated by necessitating a gradient in the magnetic field.^[^ ^299^ ^]^ Here, we will discuss some instances of magnetic actuation‐based origami functionalities that will provide a better understanding of the concept.

**Figure 11:**
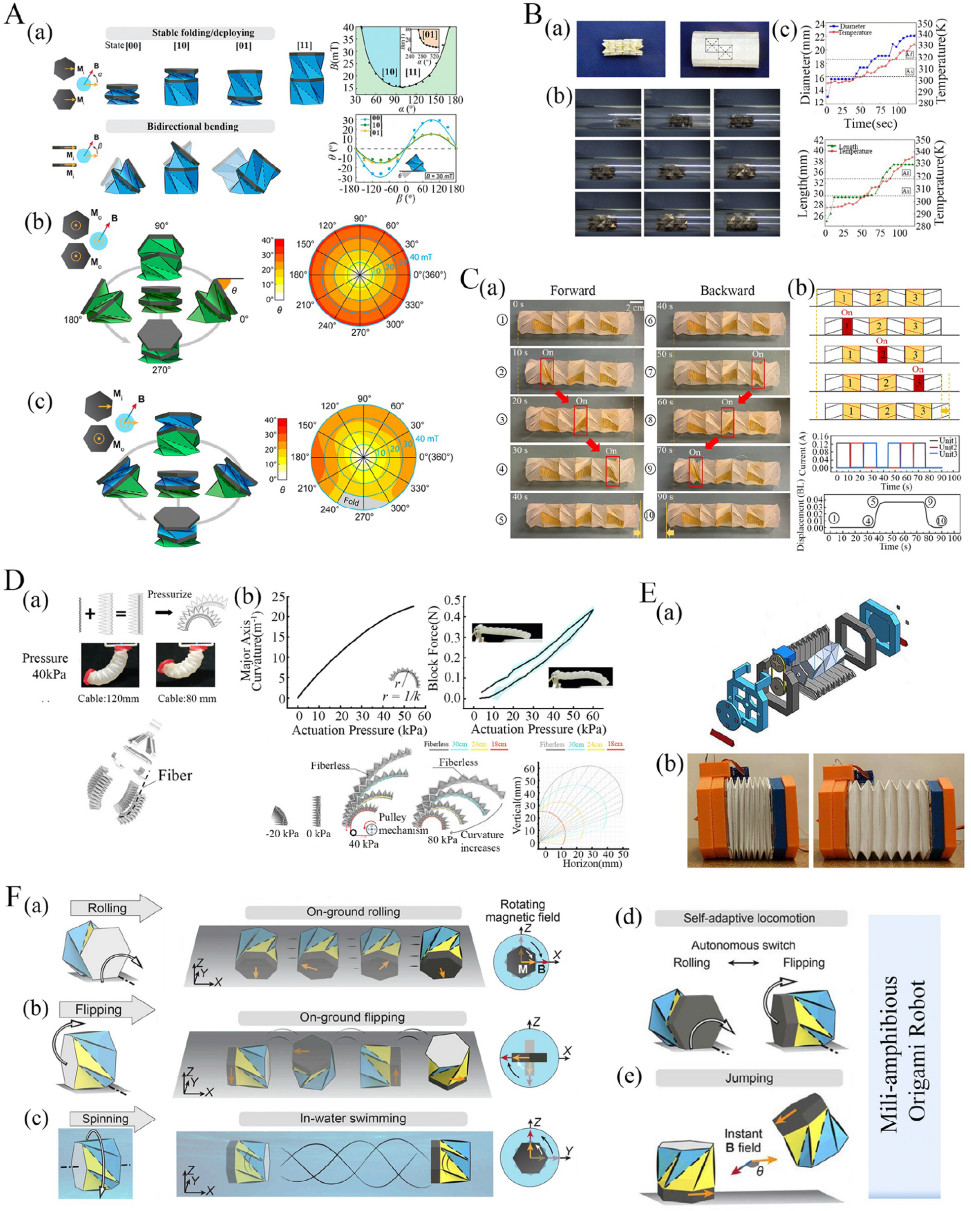
Multi‐physical actuation for programmable origami metamaterials. A) Magnetically actuated robotic tubes (a) State folding/deploying, bidirectional bending and phase diagram (b‐c) Omnidirectional bending and multi‐modal deformation showing experimental polar plots (Reproduced under the terms of the 4.0 license.^[^ ^281^ ^]^ Copyright 2021, The Authors). B) Thermally actuated self deployable origami stent graft (a) Fully folded and deployed configuration (b) Self‐deployment motion of stent under temperature variation (c) Temperature, geometric parameters vs heating time plot (Reproduced with permission.^[^ ^291^ ^]^ Copyright 2006, Elsevier). C) Soft origami robots with reprogrammable electric actuation showing bi‐directional locomotion (a) Soft robot in one cycle of forward motion followed by backward locomotion (b) Sequential activation and deactivation shown with programmed current input and corresponding rigid body displacement (Reproduced with permission.^[^ ^150^ ^]^ Copyright 2024, National Academy of Sciences). D) Origami gripper based on pneumatic actuation (a) Prototype of actuator with same pressure and variable cable lengths (b) Performance of gripper under curvature, blocked force, and scalable work range (Reproduced under the terms of the 4.0 license.^[^ ^292^ ^]^ Copyright 2021, The Authors). E) Origami‐based metameric crawling robots with electrical actuation showing exploded CAD model and locomotion (Reproduced with permission.^[^ ^293^ ^]^ Copyright 2017, ASME). F) Magnetically actuated mili‐amphibious origami robots showing variable locomotion capabilities (Reproduced under the terms of the 4.0 license.^[^ ^294^ ^]^ Copyright 2022, The Authors).

Origami‐based soft robots with various crease configurations made of iron sheets are actuated using permanent magnets to exhibit locomotive behaviors such as peristaltic motion, rolling, and turning in an environment such as sand, sandpaper, and board surfaces.^[^ ^300^ ^]^ It is noted that the displacement and velocity during actuation vary based on distinct design factors and environmental conditions. A compelling example is the Kresling robotic arm that draws (bio‐)inspiration from the integrated ability of octopus arms.^[^ ^281^ ^]^ The Kresling robotic arm utilises magnetic plates attached at the top and bottom of each unit cell. It demonstrates controlled multimodal deformation, including bistable folding/deploying, omnidirectional bending, and integrated deformation achieved through precise magnetic actuation (refer to **Figure** **11**A). The Kresling folding mechanism can achieve either deploying or bending but cannot simultaneously possess both. In the initial magnetization phase, both magnetic plates are designed for in‐plane magnetization in a folded state [00], with binary codes representing the assembly state. In‐plane torque is generated upon application of an in‐plane magnetic field, facilitating transformation between various folded states (refer to Figure 11A(a)). In the second magnetization phase, an out‐of‐plane magnetic field is applied to attain different bending states, as depicted by the experimental results (refer to Figure 11A(b)). In the third magnetization phase, both magnetic plates are designed with out‐of‐plane magnetization, and the application of an in‐plane magnetic field enables omnidirectional bending, as demonstrated in all directions by a polar plot (refer to Figure 11A(c)). Finally, in the fourth magnetization phase, the top Kresling plate undergoes in‐plane magnetization, while the bottom plate undergoes out‐of‐plane magnetization under the influence of an in‐plane magnetic field. This combination yields integrated deformation, demonstrated in all directions by a polar plot in Figure 11A(d).

Another example of a magnetically actuated robot is the milli‐scale robot, which is capable of wireless operation to navigate narrow spaces and morph its shape for specific tasks.^[^ ^294^ ^]^ The amphibious robot is designed based on the Kresling origami (refer to Figure 11F). A magnetic plate is fixed to the end of the Kresling origami. In the presence of a continuous magnetic field, the robot's magnetization aligns with the magnetic field, resulting in continuous locomotive motion such as rolling, flipping, and spinning. The specific locomotion mode demonstrated by the robot depends on the rotational axis of the robot and the robot's interaction with the surrounding environment. When the robot is on the ground, the locomotion modes of the robot, such as rolling and flipping, are achievable by rotating the magnetic field either perpendicular or parallel to the longitudinal axis of the robot, respectively (refer to Figure 11F(a,b)). In comparison, when in water, the robot swims (refer to Figure 11F(c)) by spinning about its longitudinal axis. When the robot encounters obstacles, the robot seamlessly switches between rolling and flipping locomotion modes without requiring adjustments to the magnetic field (refer to Figure 11F(d)). However, when the robot faces a large obstacle that cannot be overcome by rolling and flipping, then the robot employs a jumping motion (refer to Figure 11F(e)) provided by an instantaneous application of magnetic field.

Cai et al. proposed a magnetically actuated tubular origami robot^[^ ^301^ ^]^ based on a modified Tachi‐Miura origami crease architecture. By mounting permanent magnets into the origami crawler architecture, they demonstrated untethered shape‐morphing and reconfiguration between two different stable states (with folding and unfolding) under a changing magnetic field. Such characteristics of the origami tube can lead to active locomotion in confined spaces or load‐bearing applications. In this work, the advantage of bi‐stability is highlighted for achieving the desired functionalities.

#### 3.2.2 Pneumatic Actuation of Tubular Origami Metamaterials

Pneumatic control‐driven actuators have been given extensive attention due to their significant advantages, including large deformations, high energy efficiency, ease of implementation, and cost‐effectiveness. Pneumatic actuation is categorized based on injection pressure into two types, i.e., positive actuation and vacuum actuation. Positive actuation involves the application of positive pressure to generate a force that moves a mechanism.^[^ ^302^, ^303^, ^304^ ^]^ In contrast, vacuum actuation involves creating a partial vacuum to induce the required force for the movement of a mechanism. In pneumatic actuation, the entire structure must remain sealed to maintain the functionality.^[^ ^305^, ^306^ ^]^ Here, we will discuss a few instances of pneumatic actuation‐based origami, which will provide a better understanding of the concept.

Origami‐inspired grippers draw inspiration from the locomotive mechanism of leeches, characterized by a dual mode of morphing by elongation and bending.^[^ ^292^ ^]^ The origami gripper is formed by integrating four origami actuators, incorporating the geometry of the Yoshimura pattern to design its structural configuration (refer to Figure 11D(a)). The origami gripper exhibits a wide range of grasping motions provided by programmable adjustments of effective length. Under the action of pneumatic actuation, empirical observations reveal that the bending curvature of the origami actuator increases nonlinearly with inflation. Furthermore, by implementing the constraints on cable limitations, the soft origami actuator can increase the bending curvature without changing the elongation by increasing the internal pressure (refer to Figure 11D(a)).

An illustrative instance of vacuum actuation is found based on Kresling origami‐inspired vacuum pneumatic artificial muscles.^[^ ^307^ ^]^ Vacuum pneumatic artificial muscle comprises a 3D‐printed four‐story origami chamber with a reversed twist to counteract torsional forces and provide translational motion. Under vacuum actuation, the chamber undergoes complete deployment, in which the increasing pressure gradients induce a contraction of the chamber. However, the origami chamber is a thin elastic cylinder, so it may cause radial contraction, except longitudinal contraction, which leads to motion failure. A rigid internal support structure is provided between successive chamber layers to mitigate such challenges. Experimental observation, conducted under varying load conditions and with or without the presence of internal support, indicates that activation of the vacuum mechanism gives rise to a greater contraction in the chamber with internal support compared to the chamber without inner support.

A distinct variant among pneumatic actuators is the soft pneumatic actuator, which is distinguished by its high power‐to‐weight ratio and inherent safety characteristics arising from the utilization of flexible and compliant materials. Soft pneumatic actuators possess flexibility in design and operation by enabling tailored adjustments in pressure to match the specific assistance requirements of wearable devices due to their easy manufacturing. Furthermore, the integration of a pneumatic system with a pouch‐type soft pneumatic actuator fabricated from layered plastic sheets with an origami‐inspired structure has demonstrated exceptional compatibility and efficiency.^[^ ^308^, ^309^, ^310^, ^311^, ^312^ ^]^

#### 3.2.3 Thermal Actuation of Tubular Origami Metamaterials

A thermal control‐driven actuator is based on the principle of thermal expansion in materials to generate stress, thereby inducing self‐folding of origami. Typically, deformation in origami is initiated by employing the actuation layer with materials possessing a high coefficient of thermal expansion. Shape memory alloy/polymer‐based actuated origami offers the distinct advantage of demonstrating a temporary shape modulation under ambient conditions. One approach is to use shape memory alloy/polymer for achieving active origami involves the utilization of pre‐stretched sheets. These pre‐stretched sheets initially have a temporary shape but undergo relaxation and contraction upon heating, thereby providing the desired origami folding.^[^ ^313^ ^]^ Here, we will discuss a few investigations on thermal actuation‐based smart systems, which will provide better insights into the concept and its implementation.

An illustrative instance of thermal actuation is a microrobot which employs both thermal and electromagnetic actuation mechanisms to transport the targeted delivery of therapeutic agents.^[^ ^243^ ^]^ The microrobot structurally consists of distinct thermal and electromagnetic layers. Initially, by utilizing the thermally actuated layer, the microrobot undergoes self‐deployment at the specified location through external thermal activation. This process enables the microrobot to trap and subsequently release micro‐objects carrying therapeutic agents. Furthermore, by employing the electromagnetic layer, which responds to external magnetic fields (discussed in the earlier subsection), the microrobot attains the necessary capability to achieve pulling and rolling motions for steering.

Waterbomb‐based origami, formed from a nickel‐rich titanium‐nickel shape memory alloy, was selected for developing biomedical stents owing to its remarkable characteristics, like shape memory effect, high coefficient of expansion, and biocompatibility (refer to Figure 11B(a)).^[^ ^291^ ^]^ The waterbomb pattern on the origami stent was artificially engineered through photochemical etching techniques. The origami stent was subjected to cooling to induce its complete folding state. The stent remained compressed during insertion into the esophageal canal via a tube. However, upon encountering the body's ambient temperature, a transformative process resulted in the unfolding of origami stent, i.e., resulting in an expansion in both diameter and length with the temperature rise (refer to Figure 11B(b,c)).

It can be concluded from the above examples that heat is a reliable, often ambient, energy source characterized by its exceptional stability and controllability. Origami motions can be effortlessly achieved through exploiting the heating processes. However, fluctuations in temperature induce modifications in the characteristics of material properties, which may increase the unpredictability of origami actuation and compromise the durability of origami structures. Such issues need further attention and the development of appropriate digital twins is recommended for different critical engineering applications considering operational and ambient environmental influences.

#### 3.2.4 Electrical Actuation of Tubular Origami Metamaterials

The electric control‐driven actuator is based on the mechanics of converting electrical energy into mechanical energy, leading to the self‐folding motions in origami structures. Electric actuation can be classified into two primary forms: thermal‐electric actuation and external electric voltage actuation. In thermal‐electric actuation, an electric field is applied to generate thermal energy within a conductor, instigating the folding motion of origami, as observed in shape memory alloys/polymers.^[^ ^314^, ^315^, ^316^ ^]^ In contrast, external electric voltage actuation involves the application of an external voltage to induce motion, as observed in ionic polymer metal composites^[^ ^317^, ^318^ ^]^ and dielectric elastomers.^[^ ^319^, ^320^, ^321^ ^]^ Here, we will discuss a few instances of thermal electric actuation and external electric voltage‐based actuation in origami metamaterials for achieving origami motion.

A compelling example of thermal‐electric actuators is a non‐rigid cylindrical Kresling origami robot which mimics the multi‐degree‐of‐freedom behavior of a caterpillar.^[^ ^150^ ^]^ The robot consists of multiple Kresling units, each operating in either an active or passive mode. The active unit includes two electro‐thermal bimorph actuators, which are effectively integrated as a part of the origami panel, ensuring the structural integrity. The active unit can deform in two modes including, axial and bending deformation, by just controlling the current inputs of the two electro‐thermal bimorph actuators. The crawling motion of the caterpillar robot involves sequential activation of the multiple Kresling segments. The crawling robot consists of an interlaced series of three active Kresling units (each with two actuators, marked as yellow) and four passive units (without actuators, marked in white). Figure 11C(b) shows the sequential activation of the Kresling units 1, 2, and 3. The sequential activation of the units is done by activating the electric current, resulting in the heating of the bimorph actuator. When unit 1 is activated in the contraction mode, the friction force generated by the far‐left passive unit is lower than that of other passive units, causing the robot's tail to slide rightward on the ground. In the subsequent step, unit 1 is deactivated and begins to cool down while unit 2 simultaneously activates. During this phase, unit 1 extends to its original length as unit 2 contracts, resulting in a net force to translate the passive unit between units 1 and 2 to the right without moving the soft robot's tail or head. Since the heating and cooling cycle of the actuators requires approximately equal duration, the deactivation of unit 1 and activation of unit 2 are programmed seamlessly. This sequence continues with unit 2 deactivating and unit 3 activating, translating the next passive unit in line. In the final step, the deactivation of unit 3 pushes the far‐right passive unit forward, completing a full forward crawling cycle. For backward motion, the activation sequence of the units is reversed (refer to Figure 11C(a)). The study shows the programmed current applied on the active units and the rigid‐body displacement of the soft robot finishing a cycle of forward crawling, followed by a cycle of backward crawling.

Another compelling example of thermal‐electric actuators is a soft, metameric, origami, earthworm‐inspired robot with the capability of segmentation and docking.^[^ ^293^ ^]^ The robot's design employs modular segments constructed from a bistable origami tower which is externally supported by two additively manufactured face plates (refer to Figure 11E(a)). The internal bistable origami tower consists of three unit cells of the same chirality, which can extend and contract to enable crawling locomotion. The modular segments feature a docking system that uses SMA wire coils, allowing the robot to autonomously detach and reattach its segments using a directional magnetic arrangement. The SMA coils operate through electro‐thermal actuation, in which the application of electrical current induces heating, causing the coils to contract and generate a torque sufficient to shear apart the magnetic connection between segments. This modularity offers enhanced manoeuvrability and flexibility in various operational environments. As illustrated in Figure 11E(b), the bi‐stability of the origami tower is effectively utilised to achieve transitions between the fully contracted and expanded states of the robot segments, enabling forward motion.

One more notable instance of thermal‐electric actuators is an origami‐inspired worm robot actuated by a nickel‐titanium coil to achieve a worm‐like peristaltic locomotion.^[^ ^322^ ^]^ This innovative origami robot draws inspiration from the waterbomb origami structure. The crease pattern of the waterbomb is artificially generated by perforating a polymer sheet with controllable density, which regulates the stiffness of resulting folds. Upon activation by an electric current, the nickel‐titanium coil undergoes heating, inducing contraction, while upon termination of the current, passive cooling occurs, resulting in the relaxation. This results in the forward peristaltic motion of the worm robot.

An additional interesting example of a thermal‐electric actuator is an origami‐inspired scalable crawling robot actuated by programmable origami and electric adhesion.^[^ ^323^ ^]^ This soft crawling robot is designed with an origami body incorporated with a shape memory alloy coil actuator and two electrostatic pads, enabling dual‐mode forward motion. The robot's motion performance variations are investigated by three distinct origami cross‐sectional shapes. The robots achieve dual‐mode crawling motion through various muscle activation schemes by using a voltage source to power the shape memory alloy coil and electrostatic pads. A comparative study of the movement of the soft robots among different origami bodies is conducted, accompanied by a comprehensive analysis of cyclic performance and speed.

An implementation of external electric voltage actuation can be found to develop a soft, active origami robot.^[^ ^324^ ^]^ This innovative robot is constructed from two paper strips coated with a compliant electrode, serving dual roles as both body and actuator. It is capable of actively achieving deformation in response to external voltage. This robot shows several remarkable characteristics, such as simple structural design, robust performance, scalability in size, and good payload capability.

Another illustration of external voltage actuation is the dielectric elastomer‐based Kresling pleat box.^[^ ^325^ ^]^ Park et al. conducted a finite element analysis to examine both single‐cell and continuous Kresling origami‐based structures and analysed distinct placements of dielectric elastomer actuators within the Kresling‐origami pleat box. In order to simulate the electric actuation, the analysis was carried out using equivalent pressure and stresses induced by dielectric elastomer as load. The analysis concluded the incorporation dielectric elastomer actuators within the Kresling‐pleat box as a promising approach. Note that the findings of the investigation are solely based on finite element analyses, which further highlight the need of experimental validation to fully understand the practical implications and performance of this innovative design.

#### 3.2.5 Challenges in Achieving Active Multi‐Physical Programmability Through on‐Demand Actuation and Control

In the preceding section, we discussed the predominant actuation approaches in the field of active and programmable tubular origami, including magnetic actuation, pneumatic actuation, thermal actuation and electric actuation. In this subsection, we are going to discuss the challenges concerning practical engineering applications further. In many cases, such challenges depend on the field of application. For example, the magnetically actuated tubular origami finds application in minimally invasive surgery tools, soft robotics in constrained environments, and deployable space structures. The pneumatically actuated tubular origami finds application in soft grippers and medical robotics, deployable shelters, emergency structures and tunable energy absorption systems. The thermally actuated tubular origami finds application in temperature‐triggered aerospace deployment and biomedical stents. The electrically actuated tubular origami finds application in adaptive antennas and reconfigurable electronics, artificial muscles and actuators, and smart textiles. We outline the associated challenges below.

*Magnetic Actuation Challenges in Origami*: 1) For large‐scale and spatially distributed systems, precise control of the magnetic field in 3D space is quite difficult; 2) The actuation of magnetically controlled origami highly relies on magnetic susceptibility or remanence of the material, which often requires the integration of magnetic particles or permanent magnets, leading to increase in weight and reduced flexibility; 3) In sensitive environment such as around electronic or medical devices, the applicability of magnetically actuated tubular origami metamaterials can be limited due to potential magnetic interference, which may disrupt the operation of nearby components or systems.^[^ ^125^, ^328^, ^329^ ^]^

*Pneumatic Actuation Challenges in Origami*: 1) While 3D printing provides a more controlled fabrication approach for tubular origami, limitations in printing resolution and the complexity of removing support material pose significant practical challenges; 2) Traditional on‐off control methods for pneumatic actuation can be insufficient for complex and dynamic applications, requiring more advanced feedback systems; 3) Balancing structural simplicity with pneumatic actuator performance remains a significant challenge, particularly for applications demanding high performance and reliability; 4) Achieving sufficient strength and durability in pneumatic origami structures, especially when dealing with high pressures or complex geometries, can be difficult.^[^ ^308^, ^310^, ^330^ ^]^

*Thermal Actuation Challenges in Origami*: 1) Actuation speed is inherently constrained by thermal inertia, as both heating and cooling cycles tend to be slow and often require substantial energy input; 2) It is difficult to achieve spatial selectivity without localised heating strategies to avoid unintended global deformation; 3) Repeated thermal cycling may induce material fatigue or structural degradation, limiting long‐term reliability.^[^ ^150^, ^331^, ^332^ ^]^

*Electrical Actuation Challenges in Origami*: 1) Traditional actuation methods using motors or external forces can be noisy and slow, hindering the precise control needed for complex origami movements; 2) high‐voltage requirements in some electroactive polymers pose safety and material challenges.^[^ ^322^, ^324^, ^333^ ^]^

## 4 Computational Modelling and Physical Realization

### 4.1 Deformation Mechanics of Origami Metamaterials

Origami possesses three general deformation modes, including folding, crease, and facet deformation. Folding deformation is the most common mode involving bending of crease along the axis to form a fold. Crease deformation denotes an alteration of crease alignment or orientation, and facet deformation is an alteration of facet shapes. Most structures require a minimum four‐degree vertex for foldability. Meanwhile, three‐degree vertex origami possesses vertex constraints and non‐foldability. Thus, origami structures having three‐degree vertex results in vertex‐level constraint, and non‐foldability due to global vertex constraint. This limitation can be overcome by inducing the in‐plane shear deformation of sheets, known as shearigami.^[^ ^334^ ^]^ In shearigami, the panel is removed from the three‐degree vertex, removing the vertex‐level constraints, and the sheet becomes kirigami. The removed panels are replaced with shear panels, causing vertex‐level constraints to be removed through in‐plane shear deformation. The shear panel removes the origami's non‐foldability, resulting in self‐foldable origami. In this section, we will briefly discuss the predominant approaches of computational modelling of origami structures capturing the common modes of deformation, followed by a detailed discussion on manufacturing and fabrication of origami tubes.

### 4.2 Computational Modelling of Tubular Origami Metamaterials

Computational modelling of origami metamaterials and metastructures involves designing the model of tubular metamaterials and analysing the structural behaviors, including deformation, foldability, stiffness, and stability under different loading conditions and external actuation. The results predicted by the computational models provide an indication of its applicability in real‐world scenarios. We discuss here the predominant approaches of computational origami modelling, including the kinematic analysis, idealized bar‐hinge based models and finite element analysis.

#### 4.2.1 Kinematic Analysis Model

Kinematic analysis is a computational method based on the study of the geometric motion of a mechanism without considering the forces acting on the mechanism. In many practical origami applications, the structure behaves like a rigid‐body mechanism, making kinematic analysis a suitable modelling approach. In this framework, the creases of origami are treated as a revolute joint, and facets of origami are considered as links. Most origami patterns consist of creases intersecting at a common vertex, resulting in a spherical linkage mechanism.^[^ ^335^, ^336^, ^337^, ^338^, ^339^ ^]^ For instance, Miura‐ori, one of the simplest origami pattern with four creases, is considered a 4R spherical linkage,^[^ ^340^ ^]^ whereas, the waterbomb base with six creases forms a 6R spherical linkage.^[^ ^341^ ^]^ Kinematic analysis is often conducted using matrix method based on the Denavit–Hartenberg (D–H) matrix notations, which systematically provides a straightforward way to describe the motion of each joint and its relationship with other joints in the origami mechanism.

**Figure 12:**
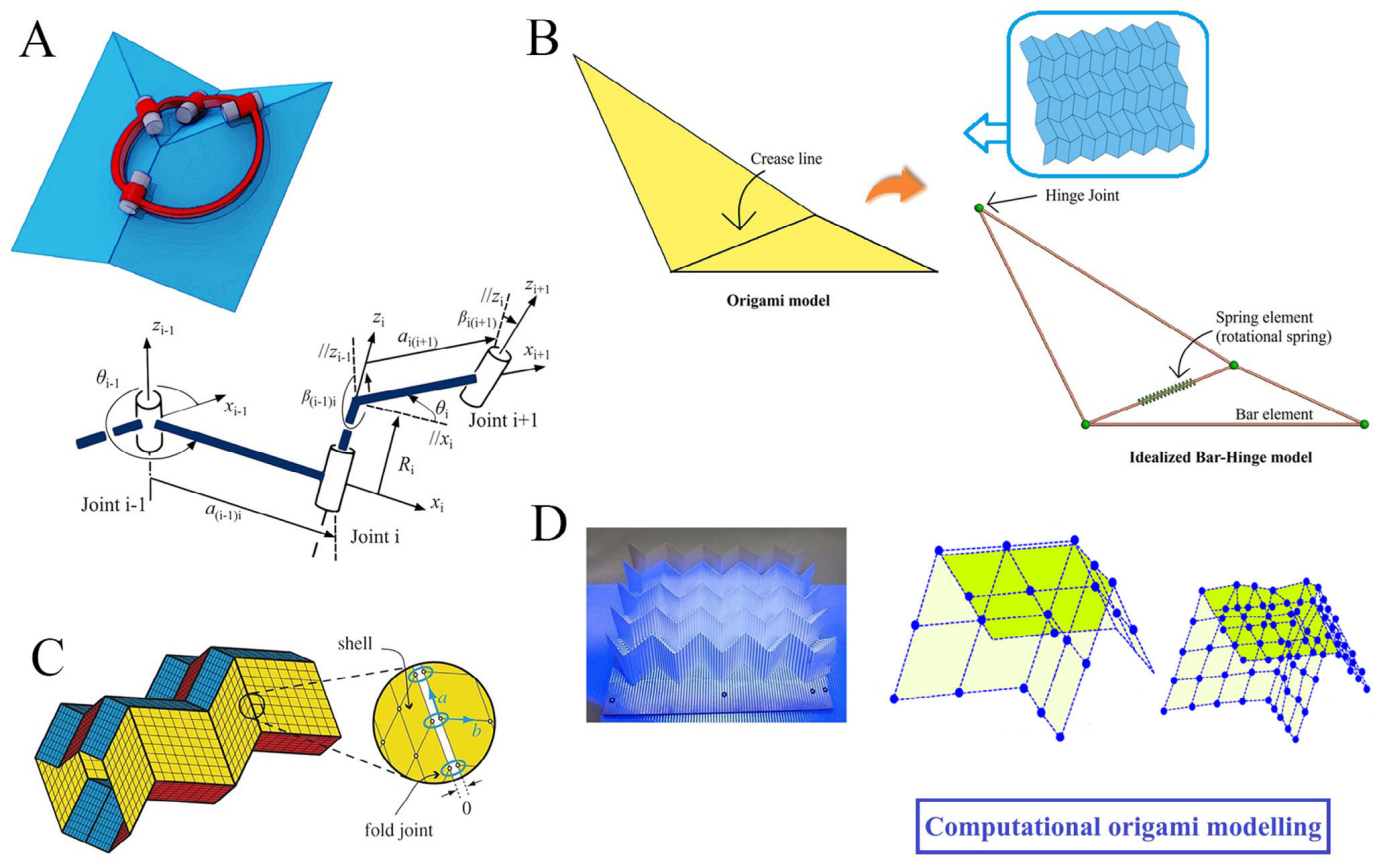
Computational modelling of origami metamaterials. A) Kinematic analysis model showing origami as a mechanism with Denavit–Hartenberg (D–H) notation of contiguous links connected by revolute joints (Reproduced with permission.^[^ ^326^ ^]^ Copyright 2020, Elsevier). B) Idealised bar‐hinge based reduced‐order model depicting an origami structure and its simplified bar‐hinge representation C) Finite element analysis model illustrating shell elements (Reproduced with permission.^[^ ^21^ ^]^ Copyright 2016, The Royal Society). D) Isogeometric analysis (IGA) model showing 3D scanning of a foldcore sample and subsequent manipulation through refinement of the NURBS control mesh (Reproduced with permission.^[^ ^327^ ^]^ Copyright 2021, International Association for Shell and Spatial Structures (IASS)).

The configuration of each joint is defined by four parameters: the link length *a*_*i*(*i* + 1)_, the link twist angle β_*i*(*i* + 1)_, the joint offset *R*_*i*_, and kinematic variable θ_*i*_ which measures the rotation between two links joined by the revolute joint (refer to **Figure** **12**A).

For a single‐loop connection with *k* links, the closure equation is given as

(1) $$\begin{array}{c} T_{21}T_{32}\text{…}T_{1K}=I_{4} \end{array}$$

where the transformation matrix *T*_(*i* + 1)*i*_ is

(2) $$\begin{array}{c} T_{(i+1)i}=\left[\begin{array}{cccc} \cos\theta_{i} & -\cos\beta_{i(i+1)}\sin\theta_{i} & \sin\beta_{i(i+1)}\sin\theta_{i} & a_{i(i+1)}\cos\theta_{i}\\ \sin\theta_{i} & \cos\beta_{i(i+1)}\cos\theta_{i} & -\sin\beta_{i(i+1)}\cos\theta_{i} & a_{i(i+1)}\sin\theta_{i}\\ 0 & \sin\beta_{i(i+1)} & \cos\beta_{i(i+1)} & R_{i}\\ 0 & 0 & 0 & 1 \end{array}\right] \end{array}$$

If *i* + 1 > *k*, then 1 is used instead. A (*i* + 1)^*th*^ coordinate system expression is converted to the *i* ^*th*^ coordinate system expression in this way.

In the context of origami mechanisms, this transformation matrix describes the relative motion between the adjacent rigid panels connected by creases, which acts as a revolute joint. Each panel is considered as a rigid link, while the creases connecting them introduce a rotation defined by the kinematic variable θ_*i*_. The D‐H matrix parameters capture the geometric relationship between these links and give a comprehensive kinematic description of the folding motion of the origami structure. By employing the closure equation, the global motion of the origami system can be modelled as a multi‐loop kinematic chain.

This method is efficient for predicting folding kinematics and motion trajectories of origami‐based mechanisms. It is possible to extend this framework for calculating the global force‐deformation behavior by assuming suitable constitutive laws for idealized rotational springs along the creases. However, such an analysis is normally not very accurate as it does not take into account all the modes of deformation energy. It is also important to note other primary limitation: it assumes rigid folding, and neglects effects such as material properties of facets and creases, joint compliance, and fabrication imperfections. As a result, kinematic analysis models cannot predict stress concentrations, fatigue, or failure, and are not suited for performance assessment under real‐world actuation or loading conditions. Despite this, it remain a powerful tool for initial design and motion planning of tubular origami structures.

Popular origami simulation software such as ORIPA,^[^ ^342^ ^]^ Freeform origami and Rigid origami simulator^[^ ^343^ ^]^ are built on the principles of kinematic analysis,^[^ ^344^, ^345^ ^]^ offering accessible platforms for modelling rigid‐foldable designs.

#### 4.2.2 Idealized Bar‐Hinge Based Model

The bar‐hinge model is a simplified representation of the deformation mechanics of origami structures, capturing the motion kinematics, where forces are considered to analyse the behavior of origami structures. This model characterises the kinematic space of origami structure using a bar framework which undergoes three fundamental forms of deformation: in‐plane stretching and out‐of‐plane crease folding, and bending. In the bar hinge model, the bars are positioned along straight fold lines and within panels to model in‐plane stiffness, whereas rotational spring are introduced at creases (along fold lines) to model the crease folding and across panels to represent the bending of panels. The bar‐hinge model reduces the degree of freedom of the overall model, resulting in efficient prediction of the mechanical behavior of origami structures.

For instance, the triangular facet is modelled by bar elements, whereas the crease is considered equivalent to a rotational spring stiffness in this idealisation(refer to Figure 12B). Assuming that the structure exhibits non‐linear elasticity, we can express the total potential energy of this simplified origami model:

(3) $$\begin{array}{c} \Pi =U_{bar}+U_{spring}-V_{ext} \end{array}$$

The potential energy contains three terms: *U*_*bar*_ denotes strain energy stored in bars, accounting for the in‐plane deformation strain energy of the origami sheet, *U*_*spring*_ denotes strain energy stored in the bending and folding deformation accounting for the out‐plane deformation strain energy and *V*_*ext*_ denotes the work done by external load. The equilibrium equation for an idealised bar‐hinge‐based model can be expressed as:

(4) $$\begin{array}{c} \frac{\partial U_{\text{bar}}}{\partial u}+\frac{\partial U_{\text{spring}}}{\partial u}=F \end{array}$$

where **u** denotes the nodal displacement vector. Note that the stored energy in the system would depend upon the axial stiffness of bars *EA*, the rotational stiffness of springs along the fold lines *K*_*f*_ and bending stiffness of facets *K*_*b*_ (for the cases where the facets are not triangular).^[^ ^346^, ^347^ ^]^ The above equation is solved following the modified generalised displacement control method, which is an arc‐length type method leading to the whole equilibrium path of the displacement‐controlled system.^[^ ^347^ ^]^

This formulation is numerically implemented by discretising the bar‐hinge model using mesh‐dependent triangular schemes. The placement of bars and rotational springs in this mesh‐dependent triangulation scheme corresponds to specific meshing configurations such as N4B5 and N5B8, which have a direct influence on the accuracy of the model and its ability to capture complex deformations. In the N4B5 scheme, the quadrilateral panel is divided into two triangles by one of its diagonals, forming 4 nodes and 5 bars belonging to each panel. This N4B5 scheme can capture the bending behavior across one diagonal axis of the quadrilateral panel, but tends to introduce skew deformation under in‐plane axial loading. In contrast, in the N5B8 scheme, the quadrilateral panel is divided into four triangles by adding an extra internode, hence forming 5 nodes and 8 bars per panel. This configuration was proposed to mitigate the skewed deformation. The additional bars in the N5B8 scheme provide improved fidelity in simulating deformation under the applied axial loading and allow to model to capture the bending across both diagonals of the panel. The bar‐hinge model can essentially be referred to as a reduced‐order modelling approach where the important features concerning load and deformation are captured accurately by adopting a simplified equivalent structural form of the origami. This type of model captures the creases folding, panel bending, panel stretching, and panel shearing deformation. However, it does not account for extensional and torsional crease motions that can occur in compliant creases. To overcome this limitation, the bar‐and‐hinge model has been extended to incorporate compliant crease behavior, particularly in the context of active origami. In this extended formulation, each compliant crease is represented using seven nodes, twelve bars, and eight rotational springs. This enhancement enables the model to further capture the extensional strain energy in folding creases, which is essential for accurately simulating the bistable behaviors observed in origami structures.^[^ ^348^, ^349^, ^350^ ^]^

To date, idealised bar‐and‐hinge model techniques have been effectively employed in a wide range of applications, including the analysis of bi‐stability and multi‐stability, capturing folding motions in tubular origami,^[^ ^285^ ^]^ investigating the behavior of compliant crease origami,^[^ ^351^ ^]^ designing origami‐based metamaterials,^[^ ^205^ ^]^optimising structural performance,^[^ ^352^ ^]^ and studying the impact of defects in origami architectures.^[^ ^353^ ^]^ However, despite their computational efficiency and utility in capturing global deformation behaviors, these models fall short in representing localised phenomena such as crease buckling, panel buckling, stress concentrations, and local plastic deformation. As such, the bar‐and‐hinge model serves best as a reduced‐order approach, suitable for initial design and global behavior predictions, but less reliable for assessing detailed structural responses under complex loading conditions.

Software such as Origami Simulator,^[^ ^354^ ^]^ Rhino3D^[^ ^355^ ^]^ and Rigid Origami Folder uses N4B5 triangular scheme to analyse the origami structure. In contrast, MERLIN2 incorporates both N4B5 and N5B8 meshing scheme to analyse the origami structure, enabling more versatile and accurate analysis of deformation behaviors in origami‐based model.^[^ ^346^, ^347^, ^356^, ^357^, ^358^, ^359^, ^360^, ^361^ ^]^

#### 4.2.3 Finite Element Analysis Model

The finite element analysis model is a numerical simulation framework used to predict the behavior of an origami model under various physical conditions. The finite element analysis model is the most detailed and most expensive approach, while it is more accurate than the bar hinge model, as it takes into account the complicated local behaviors such as local buckling, stress concentration, and material non‐linearity that occurs during the folding process of origami structures. The finite element model commonly represents panels using two types of elements to analyse origami structures: shell and 3D solid (refer to Figure 12C).^[^ ^59^, ^104^, ^362^, ^363^, ^364^, ^365^ ^]^

The shell‐based finite element analysis model is seamlessly used due to the smaller thickness of the origami panel. In this approach, the panels are considered as shell elements, and the connection between the panels can be considered as rotational hinges or fixed, or rigid creases. The first approach is to connect the shell elements through rotational springs^[^ ^20^, ^21^, ^230^ ^]^ which is particularly useful to analyse the mechanical properties of origami tubular structures. This approach has been adopted to analyse the tubular origami structure in order to obtain the load‐bearing capacity and the eigen properties of the origami tube.^[^ ^20^, ^21^ ^]^ The second approach is to connect the shell elements through fixed or rigid fold lines. This formulation is usually used to study the behavior of origami‐inspired devices such as metamaterials, crash boxes, and sandwich cores. This method has been used to analyse both the static^[^ ^366^ ^]^ and dynamic response,^[^ ^367^ ^]^ evaluate the energy absorption capabilities,^[^ ^167^, ^368^ ^]^ investigate the influence of imperfection sensitivity,^[^ ^369^ ^]^ and capture the complex material failures in origami cores.^[^ ^370^ ^]^ In this method, the deployment of the origami structure is restricted, resulting in a kinematically over constrained system. In this system, typical deformations include panel buckling and stretching, while local crease deformations are generally considered negligible in terms of their effect on global behavior.

A 3D solid‐based finite element analysis model is used to analyse the origami structure having a finite thickness.^[^ ^243^, ^371^ ^]^ This approach requires fine meshing across the panel thickness to accurately capture bending behavior, making it computationally demanding. As a result, it is usually used for cases where thickness plays a critical role in the structural response. One significant drawback of the finite analysis method is that it can be time‐consuming, particularly when the origami base is non‐repetitive. Although the finite element analysis method offers several advantages over the reduced order method (particularly for interogating the local structural behavior such as stress concentration and failure), the reduced order method like bar‐hinge based modelling is often preferred over finite element analysis in global behavior analysis due to its lower computational cost and greater conceptual clarity. The most commonly used finite element analysis‐based software is ABAQUS,^[^ ^372^ ^]^ ANSYS,^[^ ^373^ ^]^ and LS‐DYNA.^[^ ^370^ ^]^

#### 4.2.4 Isogeometric Analysis (IGA) Model

The isogeometric analysis (IGA) model is a high‐fidelity computational modelling approach which combines a CAD‐based geometry model with numerical simulation by using either non‐uniform rational basis‐splines (NURBS) or spline‐based surfaces directly in the analysis. Traditional finite element analysis models approximate geometry through meshing, while the isogeometric analysis model enables exact representation of complex folded structures, reducing discretisation errors and mesh dependency. This makes the isogeometric analysis method particularly advantageous for simulating origami metamaterials. In the context of origami foldcore structures, the isogeometric analysis model defines geometry using non‐uniform rational basis‐splines (NURBS), parametrised by major and minor crease line vectors within a control mesh. This direct geometric control through simple modification of the control mesh enables the inclusion of experimentally measured imperfections, including crease curvature and panel deformation. A 3D scan of manufactured fold core samples can be utilised to extract real‐world geometric data, which is then incorporated into the IGA model through local manipulation of control points (refer to Figure 12D). In the isogeometric analysis model, the incorporation of imperfections without altering the model topology is enabled by three different refinement techniques, such as knot insertion (similar to *h*‐refinement in classical finite element analysis model), order elevation (similar to *p*‐refinement in classical finite element analysis model), and *k*‐refinement (no equivalent in classical finite element analysis model). A shell formulation with Reissner‐Mindlin elements is typically employed in the isogeometric analysis model simulations to capture the bending and buckling behavior of thin‐walled origami structures under loading. These simulations often use implicit time integration schemes and boundary conditions applied through non‐uniform rational basis‐splines based specific commands to enforce realistic deformation constraints. The isogeometric analysis model, compared to the classical finite element analysis model, offers improved accuracy, convergence, and reduced degrees of freedom, making it particularly suitable for optimisation and parametric studies.^[^ ^327^, ^374^, ^375^, ^376^ ^]^ The geometric exactness and adaptability of the isogeometric analysis model make it an emerging and powerful tool for computational analysis and design of origami structures. However, the IGA approach is still in the nascent stage for origami modelling and a significant attention is needed for incorporating the complex behavior of creases in foldable origami.

### 4.3 Manufacturing and Physical Realization of Origami Tubes

Tubular origami metamaterials have evolved from conceptual designs to practical real‐world applications as depicted in Figure 4 and the concerning discussions. This evolution has increased the need to manufacture robust and durable tubes that can be fabricated in large quantities through automated and repeatable processes. Traditionally, these origami‐based tubes were created through manual paper folding. While such an approach is suitable for artistic exhibition and developing preliminary insights for engineering designs, it is not often suitable for real‐world engineering applications. Thus, alternative methods have been developed to suit the demand of modern engineering industries (refer to **Table** **1**). Most of these manufacturing methods aim to reduce stress concentrations at the creases, enhance consistency and repeatability, improve fatigue resistance, ease in actuation and self‐deployment and increase load‐bearing capacity, making them more suitable for practical engineering applications.

**Table 1:** Manufacturing approaches of tubular origami metamaterials.

Fabrication process	Description
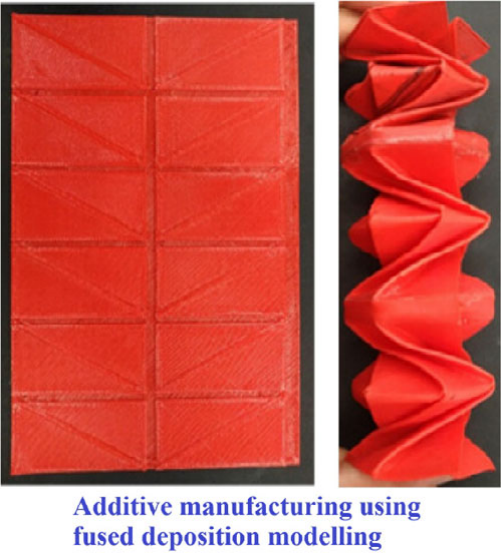	Fused deposition modelling method is a layered manufacturing technology which creates parts with complex geometry by layering the extruder material. This method involves creating 3D CAD model including the pockets of creases and can programmably control the mechanical properties. This method can also be used to create multimaterial origami such as thick origami in which the rigid panel consists of a rigid material wrapped by soft material.^[^ ^289^, ^382^ ^]^ (Reproduced with permission.^[^ ^286^ ^]^ Copyright 2020, Sage Publication).
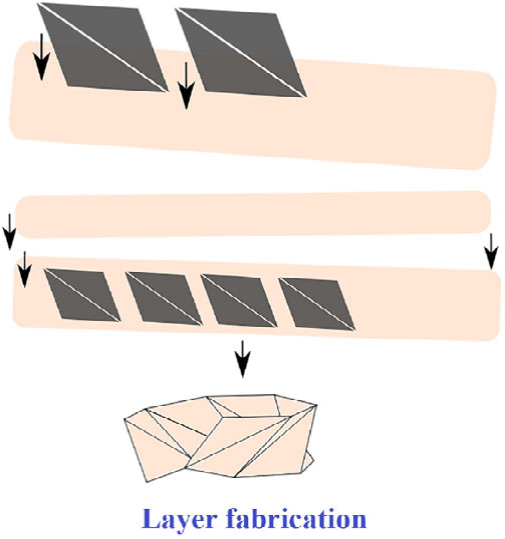	The fibre sheet is glued between air tightened fabric and form a sandwich structure that improves the air tightness. Rolling the sandwich structure with two caps results in the conversion of 2D structures to 3D structure. The origami can have programmable stiffness depending on crease pattern.^[^ ^282^, ^377^ ^]^
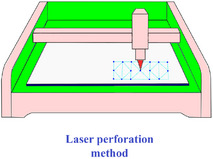	Laser perforation method involves laser cutting a series of small holes on a straight line. The perforations are symmetrical, and as only a part of the fold line is affected by the laser, the fold is robust to variation with no repeatability issue. The adjustment of perforation density results in controlling the stiffness of the fold.^[^ ^291^, ^322^ ^]^
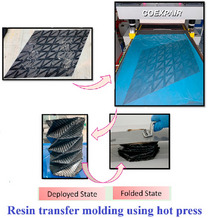	Resin Transfer Molding (RTM) is a manufacturing process used to create high‐strength composite materials by injecting resin into a mold containing a pre‐shaped fiber reinforcement. When combined with a hot press, this technique can be adapted to fabricate origami‐inspired composite structures with precise folds and complex geometries. The architecture pattern prepreg is bonded to dry fabric by curing the prepreg resin using hot press. During curing, the resin flows through the dry fabric onto which the prepreg was resting, causing the structure to become rigid. The folding region is formed by combining dry carbon fabric with rubber polymer to obtain the required strain energy for recovery and desired bending stiffness.^[^ ^383^ ^]^ (Reproduced with permission.^[^ ^378^ ^]^ Copyright 2020, Elsevier).
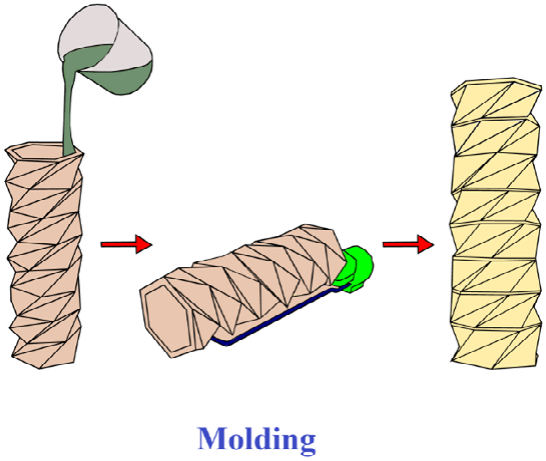	Molding uses a 3D printed mold resembling the shape of tubular origami. Uncured silicone resin or thermoplastic polyurethane (TPU) is poured into the mold. To create a homogeneous thin layer, the mold is spun at a speed of 10 rad s^−1^ ensuring an even coating of silicone on inner surface of mold. As the deposited layer gradually solidifies, it conforms to the shape of mold, and finally the entire assembly is then cured at 45^*o*^C for 30 min before the mold is removed.^[^ ^380^, ^384^ ^]^
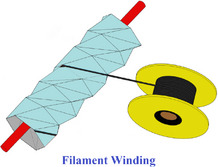	Filament winding technique uses tubular origami‐shaped mandrel and a carbon fiber tow which is allowed to rotate about and translate along its axis. The first fiber layer is wrapped around the mandrel, held down with masking tape, and coated with resin before adding another fiber layer. The tube is cured at 130^*o*^ F for 6 h with controlled rotation to prevent resin accumulation, then cooled for 12 h. After curing, excess material is removed to leave a origami tube (Reproduced with permission.^[^ ^250^ ^]^ Copyright 2022, Elsevier).
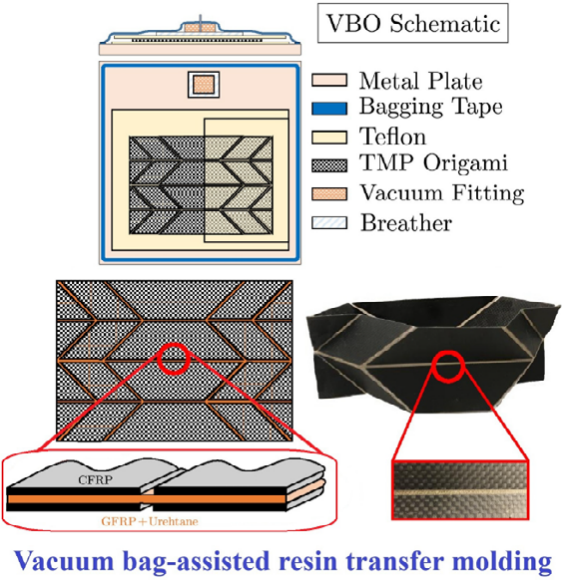	Vacuum bag method uses pre‐impregnated plies instead of dry fibres. A sandwich origami shape is formed from carbon fibre reinforced at top and bottom, and Uthrene resin at middle, which is packed inside vacuum bag. The resulting origami structure is obtained by pulling the air out of system, leading to a self‐deployable, high‐compactness structure.^[^ ^385^ ^]^ (Reproduced with permission.^[^ ^379^ ^]^ Copyright 2020, Elsevier).
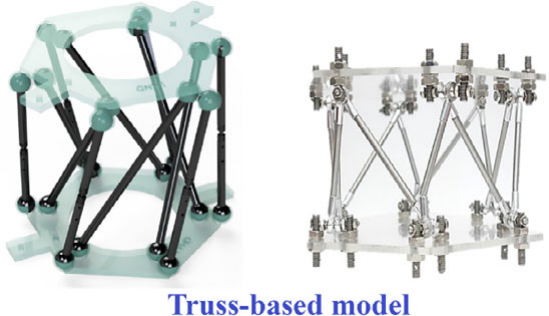	In truss‐based models, facets and creases are substituted with elastic or rigid bars (linkages) that may have linear springs attached at one or both end. The ends of linkages rest on distinct joints, including frictionless ball socket joints, universal joints, and universal spring joints, which are attached to a rigid top and base plate. The flexibility in linkages can be incorporated by placing tensile springs in the middle of linkages.^[^ ^61^, ^386^ ^]^ (Reproduced with permission.^[^ ^121^ ^]^ Copyright 2024, IOP Publishing).
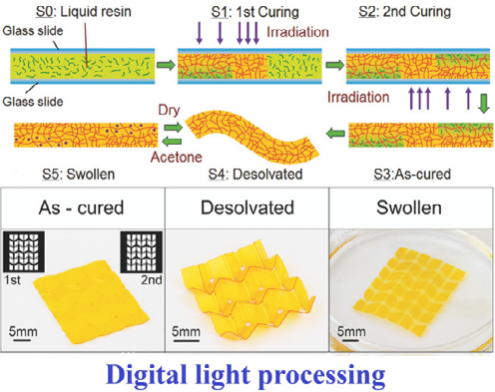	The digital light processing method uses photo polymerization printing to fabricate origami architectures. This method involves light projection on the photo‐curable resin, which cures the resin layer. The cross‐linked density of the resin can be varied by controlling the light intensity and total exposure energy. Origami structure is fabricated by varying the cross‐linked density in which the fully cured panels would not deform when exposed under high intensity, and the differentially cross‐linked hinges could bend after desolvation exposed under low intensity.^[^ ^387^, ^388^ ^]^ (Reproduced with permission.^[^ ^381^ ^]^ Copyright 2016, Wiley).
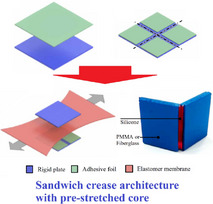	In the sandwich form of origami architectures, two face sheets are comparatively stiff and the core is a stretchable material. Each of the face sheets is disconnected along the crease lines, while they are attached to a pre‐stretched polymeric membrane. The sandwich architecture in origami folding can show programmable crease stiffness, rest angles and improved mechanical functionalities.^[^ ^389^ ^]^ (Reproduced with permission.^[^ ^390^ ^]^ Copyright 2018, The Authors, some rights reserved; exclusive AAAS).
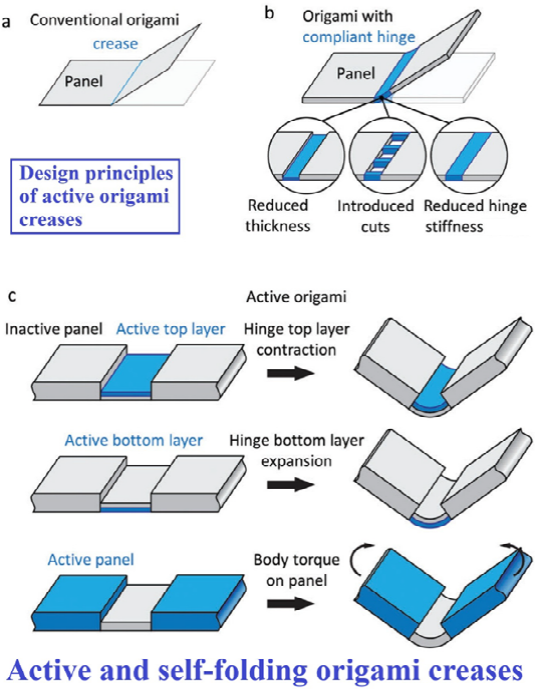	The conventional method followed in origami papers and other thin polymers includes folding these through the crease line to create plastic deformation and reduced stiffness along the fold line. Other slightly advanced approaches adopted for engineering‐grade materials include different ways of reducing bending stiffness along the crease line such as reduced thickness, introduced cuts and introduction of softer material along the crease. Active origami and self‐folding can be achieved by making the crease actively bendable or making the facets active so that they can mutually interact.^[^ ^391^ ^]^ (Reproduced with permission.^[^ ^313^ ^]^ Copyright 2016, Wiley).

For realising physical prototypes, the first step is selecting the appropriate material. While origami traditionally uses paper, for engineering tubular origami, materials like metal, plastic, or composites are commonly used due to their strength and ability to hold their shape. The material needs to be flexible enough to fold but durable enough to retain its form once shaped. Based on appropriate designs of crease patterns, most common methods used to fabricate tubular metamaterials include 3D printing using the fused deposition method,^[^ ^286^ ^]^ layer perforation method,^[^ ^291^ ^]^ layer fabrication method,^[^ ^377^ ^]^ resin transfer method using a hot press,^[^ ^378^ ^]^ vacuum bag‐assisted resin transfer molding method,^[^ ^379^ ^]^ molding,^[^ ^380^ ^]^ filament winding,^[^ ^250^ ^]^ and digital light processing method^[^ ^381^ ^]^ (refer to Table 1 for a concise summary).

After the material is fabricated, the folds are manually or mechanically applied. In some cases, automated folding techniques are used, especially for high‐volume manufacturing. Besides manufacturing of traditional origami structures with facets and creases, truss‐based physical models^[^ ^61^ ^]^ have been proposed recently. This is an idealized reduced‐order manufacturing approach derived based on the idea of modelling discussed in Section 4.2.2, wherein it retains the effective origami motion behavior.

Generally, origami is divided into two types depending on the elastic energy landscape. Rigid origami involves folding and unfolding without inducing deformation within the panel, with energy predominantly stored within the folding creases. Deformable origami involves energy storage within creases and panels throughout folding.^[^ ^54^ ^]^ In all the above‐mentioned manufacturing methods, the selection of appropriate material and the model's thickness can result in the formation of deformable and rigid origami, while hybrid origami and kirigami‐based metamaterials employ the concept of both cutting and folding.

A rapidly progressing research area is active origami metamaterials with self‐folding capability incorporating a range of stimuli‐responsive materials, as discussed in the preceding sections. The underlying manufacturing concepts including stimuli‐active material are presented in Table 1. Apart form adopting stimuli‐responsive materials in the origami architecture, a promising approach to develop smart origami is through hybrid designs such as tensegrity‐origami, the design and manufacturing approaches of which can be found in literature.^[^ ^392^, ^393^, ^394^, ^395^ ^]^ In this context, it may be noted that, while the fundamental mechanics of crease deformations and motion behavior according to architected crease patterns in tubular origami remain scale‐independent, the ability of physical realization in micro and nano‐scale is an evolving area of research.^[^ ^66^, ^101^, ^396^, ^397^, ^398^ ^]^

## 5 Evolving Trends and Future Roadmaps

Origami‐inspired mechanical metamaterials and their multi‐physical self‐folding capabilities are not older than a decade as a recognized scientific field, and it is continuously evolving with new ideas and advances in manufacturing capabilities across the length scales. Intense research activities over the last few years have brought forth a few emerging trends and research directions that need further attention in the near future. In this section, we will highlight such research trends and evolving areas in the field of tubular origami metamaterials and metastructures that warrant specific attention in the coming years to achieve remarkable engineering milestones with meaningful impact.

### 5.1 Optimum Multi‐Physical Functionalities with Appropriate Identification of Intrinsic Materials

Tubular metamaterials have been investigated in recent years, with most work focusing on demonstrating tailored mechanical features, including stiffness, Poisson's ratios, surface profile modulation and multi‐stability, under the application of mechanical loads or considering kinematic motion behavior. The emerging works in the case of tubular metamaterials, which involves on‐demand programming of multi‐stability and shape morphing properties, acoustic bandgaps, coefficient of thermal expansion, and electromagnetic wave propagation, will lead to broadening the application landscape of tubular origami architectures, besides dealing with purely motion kinematics, stiffness and other mechanical properties. Such research will cater to the demand for multi‐functionality in advanced mechanical systems through adopting appropriate multi‐objective optimisation goals. In this context, more efficient exploitation of stimuli‐responsive physics of smart materials coupled with tailored crease architectures would lead to enhanced dynamic control and contact‐less programmability.

The research on tubular metamaterials has demonstrated that materials such as paper and polymer sheets primarily represent the folding mechanism of tubular metamaterials. While the folding mechanics are mostly independent of materials as long as the intended mechanical characteristics of facets and creases are satisfied, a wide range of conventional materials used to demonstrate mechanical capabilities in origami structures are less applicable in real‐world applications such as space deployable structures, robotics, and biomedical devices. Therefore, exploring new material alternatives for desirable mechanical properties considering surrounding environments and multi‐functionalities is necessary for solving real‐world critical engineering problems. The use of biodegradable and eco‐friendly materials to align with sustainability goals may also be considered in this context for applications in packaging, construction, and disposable devices.^[^ ^399^, ^400^ ^]^

The incorporation of smart materials such as shape memory alloys/polymers, piezo‐active elastomers, and magnetic materials, which can self‐fold themselves under actuation, will find great potential in future applications. In the context of smart materials, the scope can be broadened as per application‐specific demands, such as photo‐sensitive active materials and the exploitation of chemically active materials, including hydrogels.

### 5.2 Inverse Design for Multi‐Objective Goal Attainment

Tubular metamaterials demonstrate unprecedented properties such as programmable stiffness, shape morphing, Poisson's ratio, energy absorption, and multi‐stability. These mechanical properties are often complex nonlinear functions of crease architectures and geometric dimensions of the base origami patterns along with folding percentage. Further, in case of active origami metamaterials, different fold percentages and stable states can be achieved as a function of a given intensity and direction of external stimuli. There exists a strong rationale of developing inverse design and optimization frameworks to achieve a range of single or multi‐objective goals for identifying the most suitable origami architectures and folding configurations, wherein the aspect of required external stimuli can further be integrated into the computational framework for self‐folding origami.

In general, the investigations on origami‐based tubular metamaterials for multi‐functional objectives reveal that the crease architectures should be chosen based on a number of conflicting objectives, where a tremendous scope of multi‐objective design optimization can be identified considering the crease architectures, different patterns, their geometries and intrinsic materials along with folding percentage. Efficient machine learning models can be exploited in such optimum designs for improving computational efficiency and exploring a large design space. The aspect of gradation (and its optimal distribution) to deal with application‐specific strain rates (for energy absorption applications) and achieving non‐uniform curvatures^[^ ^119^ ^]^ (for large‐scale shape morphing) is largely unexplored, which holds significant potential for achieving true multi‐functionality. Another aspect concerning different mechanical functionalities that needs significant attention is the practically realistic situation of having combined loading scenarios, including axial, bending, sharing and twisting modes, and subsequent inverse design of crease architecture (particularly for applications concerning energy absroption and load‐bearing structural components).

### 5.3 Higher Order Metamaterial Architectures

Tubular origami architectures can be idealized as one‐dimensional elements with rich and programmable constitutive properties and there is a tremendous scope of having higher‐order architectures by assembling such elements in a periodic network.^[^ ^401^, ^402^ ^]^ This will open up a new field of research with expanded programmability under high‐dimensional input parameter space. In this context, it is worthy to highlight that origami‐based tubular architectures are often suitable for seamless integration in larger host structures to enrich the behavior of the overall system.^[^ ^247^ ^]^

### 5.4 Mechanical Computing

Mechanical computing using tubular origami is an emerging field that combines principles of mechanical engineering, computational design, and origami‐based folding of structures including their bi‐stability characteristics.^[^ ^403^, ^404^ ^]^ Based on the exploitation of bistable configurations, applying a pre‐defined force can switch the structure between two stable states, representing the binary digits, 1 or 0. By connecting multiple such bi‐stable structures in a network or considering multi‐stable origami architectures, one could create a mechanical equivalent of a computer processor, capable of performing basic calculations or logic operations. Further, Cellular Automata‐inspired multistable tubular origami architectures can be developed for performing complex computational tasks by incorporating reservoir computing.^[^ ^220^ ^]^

### 5.5 Selective Radial bi‐Stability Programming

An interesting, yet vastly unexplored aspect in tubular origami architectures is radial bi‐stability programming. It may be noted that since each of the units in a wide range of tubular metamaterials (such as waterbomb tube) is inherently bistable along the radial direction, it leads to a possibility of further programming the mechanical behavior based on selective stable state distribution (inward or outward state).^[^ ^405^ ^]^ For example, in a waterbomb tube, the outward state leads to more structural stiffness locally and based on a designed distribution of such stable states, the constitutive behavior including spatial shape profile, Poisson's ratios, dynamics and wave propagation behavior can be altered post‐manufacturing. Further, active actuation can be incorporated in the radial direction for on‐demand programmability of the constituiting origami units.

### 5.6 Programmable Dynamics Including Multi‐Physical Actuation

The use of origami tubes as a signal attenuation and controlling device is a subject of extensive research in the field of dynamics and wave propagation. However, the aspect of on‐demand programmable signal attenuation and band‐gap tuning is largely unexplored. This can be achieved by incorporating active materials in the origami architecture for self‐folding and contactless post‐manufacturing tunability. Further, there exists a tremendous possibility of mode steering based on axial‐twist coupling in architected tubular metamaterials like the Kresling origami.

### 5.7 Enhanced Programmability Under Multiple Physical Stimuli

The interaction of multiple physical stimuli, including magnetic, thermal, electrical, mechanical and pneumatic fields, opens an avenue for developing more robust, adaptive, and programmable tubular origami metamaterials. While each actuation method has distinct advantages, the leverage of hybrid actuation approaches may make it possible to achieve complex, multifunctional responses that would not be possible with a single physical stimulus alone. For instance, thermal actuation provides a smooth and gradual shape change, while electrical or magnetic actuation can provide fast, localised control. Pneumatic actuation offers large, reversible deformations with minimal structural complexity. The integration of these multiple physical stimuli fields leads to future metamaterial systems that could achieve tunable stiffness, direction‐dependent responses, and spatially selective actuation.^[^ ^243^ ^]^ Such kind of programmability is crucial for future applications demanding adaptability in real‐time, such as soft robotics operating in unstructured environments, deployable aerospace components, or smart biomedical devices.

### 5.8 Thick Panel Tubular Origami

Most of the origami architectures in tubular form used till now primarily uses paper and polymer sheets, assuming the thickness of the panel is negligible. However, for many large‐scale practical applications, the thickness of the panel can not be ignored. There exists a strong rationale to extend the current works for considering the mechanics of thick‐panel origami in tubular architectures including suitable manufacturing processes.

### 5.9 Geometrical Derivatives of Tubular Origami

Most studies in the field of tubular origami concentrate on cylindrical architectures for a range of programmable mechanical characteristics as a function of the crease geometry. However, there exists a tremendous untapped potential to investigate other geometric derivatives like conical configurations^[^ ^247^ ^]^ and a range of other tubular configurations with spatially varying diameter for modulating static and dynamic properties as per application‐specific demands.

### 5.10 Development of Hybrid Local‐Global Origami Simulation Framework

As discussed in Section 4.2, besides kinematic analysis for capturing the global motion behavior, two of the most prominent origami simulation approaches are bar‐hinge based reduced order modelling and a detailed finite element modelling. While the bar‐hinge based approach is computationally efficient and provides a global behavior of the origami in terms of motion kinamatics and associated forces, this simulation framework lacks the ability to address critical local behavior such as stress concentration and failure. On the other hand, the finite element simulations are quite detailed and computationally expensive ‐ but these are able to analyse the global load‐deformation features as well as the local attributes like failure and stresses. Considering the optimal design aspect in mind, where a large number of iterations need to be performed, there exists a strong potential for developing a hybrid local‐global origami simulation framework to reduce the computational intensiveness of pure finite element simulations. In such a hybrid framework, the bar‐hinge based model would capture the global behavior, while the finite element simulations can be integrated at the local levels to investigate failure and stresses. Along with conventional finite element simulations, there exists a further potential to integrate advanced and evolving numerical techniques^[^ ^406^, ^407^ ^]^ at the local level to capture the distribution of stresses, initiation and propagation of damage etc.

### 5.11 Artificial Intelligence (AI) and Machine Learning Including Generative AI

The investigations on origami‐based tubular metamaterials for multi‐functional objectives reveal that the origami designs should be chosen based on a number of conflicting and often uncorrelated objectives, with which there exists a functional nonlinear relationship of the crease architecture and folding percentage. Such relationships are computationally intensive or cumbersome to characterize experimentally in most instances, particularly when it is necessary to investigate the crucially important parametric variation of the dimensions and architectures of creases. Further, the static and dynamic properties of the origami tube change at different instances of the motion path i.e. as a function of the folding percentage. It is necessary to have an efficient computational framework relating the design input parameters (such as crease architectures, intrinsic material properties, and folding percentage) and output quantities of interest (such as instantaneous stiffness, Poisson's ratios, spatial shape, energy landscape and stability features, dynamics and wave propagation behavior), leading to the identification of optimal origami configurations. Machine learning can be exploited in this context to develop data‐driven computationally efficient mapping and investigation of the design space in depth.^[^ ^173^, ^217^, ^408^, ^409^, ^410^, ^411^ ^]^ It has been realized by a large section of the scientific community that while the physics of materials and the related mechanics of origami architecture provide the governing rules for achieving a range of effective mechanical properties, the rise of artificial intelligence can truly spearhead predictive design for attaining multi‐functional objectives. Further, there exists a significant potential of exploiting physics‐informed machine learning^[^ ^180^ ^]^ and image‐based artificial intelligence algorithms^[^ ^412^ ^]^ for improved prediction, identification and unraveling new insights.

The aspect of generative artificial intelligence (Gen AI) has not been adequately explored in origami‐based metamaterials or general metamaterial designs. AI is not just about data analytics or developing a prediction tool (data‐driven, physics‐informed or image based) – it can also drive innovation in thoughtful design of novel bespoke metamaterial architectures, as if the AI has its own brain to imagine unseen patterns. Generative models, including variational autoencoders, diffusion models, autoregressive models, and generative adversarial networks, can be leveraged to generate or imagine novel origami creases and forms that has never been explored before, by analysing data (i.e. learning) from existing architectures. This progression brings us closer to autonomous metamaterial discovery with dynamic adjustments, where AI will generate ideas for new architectures that can further be computationally tested (based on physics‐based computational modelling approaches discussed in Section 4.2) in an iterative closed‐loop process.

### 5.12 Miniaturization and Nano‐Scale Tubular Metamaterials

Most studies on tubular origami metamaterials have primarily focused on the micro and macro scales. The trend of miniaturization, as sparked due to the progress in precision manufacturing at micro and nano scales, supports advanced applications in soft robotics, targeted drug delivery, and optical devices. The scientific community has recently started focusing on the field of nano‐tubular metamaterials^[^ ^101^ ^]^ including graphene origami architectures,^[^ ^413^, ^414^, ^415^ ^]^ and this field has a long way to go in developing functional materials and systems with fundamentally tunable features. Besides advanced nano‐fabrication capabilities, the development of such nano‐scale architectures will need appropriate modification of the computational frameworks accounting for the scale effects.

### 5.13 Additive Manufacturing and 4D Printing of Tubular Metamaterials

Extensive research in the field of manufacturing tubular origami metamaterials has enabled the physical realization of complex crease patterns using additive manufacturing. However, some of the challenges that need further attention in this context includes the appropriate utilization of multi‐material 3D printing for achieving crease folding with softer materials and stiffer facets, durability and fatigue performance of 3D printed origami structures considering that most of these metamaterials are likely to undergo a large number of loading and unloading cycles, inherent defects of additively manufactured components and their effect on the long‐term mechanical features, expansion of the material selection choices for origami additive manufacturing according to application demands, detailed characterisation of the stochastic variation involved in the additive manufacturing process, additive manufacturing capabilities at smaller length scales, and time‐consuming nature of origami 3D printing.

One of the emerging trends in tubular origami metamaterials is on‐demand active modulation and self‐folding through stimuli‐responsive actuation. This may be achieved by incorporating active materials in the origami architectures based on 4D printing.^[^ ^416^ ^]^ 4D printing concerning origami is still in the initial stage of development, and more attention is needed to increase the precision and functionality.

### 5.14 Scalability

For industry‐scale applications of various origami metamaterials two aspects concerning scalability need scientific attention: 1) addressing challenges in scaling up the production of origami metamaterials while maintaining precision and the intended mechanical functionality 2) development of efficient assembly techniques including self‐assembly for large‐scale manufacturing.

While downscaling to the microscale and nanoscale, achieving the intricate folds required by origami patterns becomes increasingly difficult. Conventional folding techniques may not be feasible, necessitating innovative fabrication methods like photolithography or atomic layer deposition. While upscaling to larger scales, maintaining structural integrity and accurate folds is challenging, especially under self‐weight or external forces. Manual folding is impractical for large‐scale or industrial applications. Automation systems, like robotic arms or self‐folding materials, need to be highly precise and adaptable to handle complex origami designs with a large number of creases. In this context, one effective approach could be breaking down large origami structures into smaller, modular units that can be independently fabricated and assembled to construct origami structures with large domain sizes. The modular origami approach can allow for better control over scalability without compromising complexity.

### 5.15 Uncertainty Quantification and Development of Digital Twins

Manufacturing of origami‐based metamaterials and metastructures is quite complicated compared to lattice‐based counterparts due to the distinct mechanical properties involved in the creases and facets that require a multi‐stage manufacturing process with creasing and folding. The manufacturing process becomes even more challenging in the case of smart origami architectures involving active materials. Besides the traditional manufacturing approaches, additive manufacturing (3D and 4D printing) has emerged as the most prominent way of realizing complicated origami architectures. However, additively manufactured structures often have different defects such as unwanted prestress, micro‐void, plastic deformation and variations in the manufactured geometry, which can significantly influence the mechanical behavior. Moreover, the mechanical properties are dependent on the type of additive manufacturing and manufacturing process parameters. Manufacturing defects and other related uncertainties need to be addressed thoroughly in origami architectures considering the effective mechanical behavior and the influence of cyclic folding and unfolding over the operational period, before these structures are adopted in advanced engineering applications. Additionally, uncertainties are often involved in the computational modeling approach including assumptions of material deformation models, their evolution over time and rest angles. A more cautionary approach with accurate modeling considering nonlinear stiffness and plastic deformation should be considered for a precise prediction of the mechanical behavior with the least variability.^[^ ^417^ ^]^

Besides manufacturing variations and uncertainties, the service‐life conditions of origami metamaterials need significant attention including the influence of the surrounding environment, material degradation, and damage accumulation over time. Long‐term mechanical properties that are particularly relevant for origami‐inspired structures due to folding and unfolding motions, such as fatigue and effects of viscoelasticity and creep have not received much attention for static or dynamic analyses. In this context, comprehensive digital twins can be developed considering the manufacturing and service‐life parameters to effectively predict and monitor the mechanical performances of origami structures throughout their life‐time.

## 6 Concluding Remarks

This article presents a critical review of an emerging class of origami‐inspired metamaterial possessing cylindrical shapes and their derivatives, wherein the tubular origami architectures can be formed by folding flat sheets of intrinsic material along strategically‐architected creases into 3D, hollow, cylindrical configurations. Increased attention on such origami‐based tubular metamaterials and metastructures has made it possible to achieve unique and unconventional mechanical behavior, often having conflicting and uncorrelated multi‐objective goals such as geometric efficiency and compactness, deployability and reconfigurability, structural integration ability in complex shapes, stiffness and strength modulation, constitutive programming and deformation mode coupling, high specific energy absorption capability, multi‐stability and programmable dynamic behavior, thereby pushing the limits of mechanical and multi‐physical properties. This paper has reviewed the critical recent progress in tubular origami metamaterials with a particular emphasis on the multi‐physical functionalities, including the on‐demand property modulation and self‐folding through stimuli‐responsive actuation, leading to diverse applications in the field of mechanical, robotics, space, electronic devices and communication, biomedical, and architecture.

Followed by a brief overview of emerging classes of tubular metamaterials based on functionalities and architecture, we emphasise tubular origami and kirigami architectures, including their geometry, functionalities and active modulation. The conventional approaches, limitations and scope of improvements for computational modelling and physical realisation are discussed systematically. As an integral part of this paper, we have presented the exploitable insights of deformation mode coupling exploring whether there can be twisting or axial deformation under the application of axial or twisting far‐field forces in a compulsory or discretionary way as a function of the crease architecture. We conclude the paper by critically analysing the evolving trends and future research roadmaps in the field of tubular origami involving the notions of optimum multi‐physical functionalities and real‐time programming, identification of sustainable intrinsic materials, inverse design for multi‐objective goal attainment, higher order metamaterial architectures, mechanical computing, selective radial bi‐stability programming, programmable dynamics including multi‐physical actuation, thick panel tubular origami, geometrical derivatives of tubular origami architectures, development of hybrid local‐global origami simulation framework, exploitation of artificial intelligence and machine learning including generative intelligence, miniaturization and nano‐scale tubular metamaterials, additive manufacturing and 4D printing of tubular metamaterials, scalability, uncertainty quantification and digital twin development.

It may be noted that origami metamaterials and structures have been widely studied and there are existing review papers in this field. However, the current paper offers a novel and focused perspective by specifically reviewing multi‐physically programmable tubular origami metamaterials, which is a relatively underexplored yet rapidly evolving subset of origami‐based systems. Having said this, it may be noted that some of the critical discussions are also valid for the generic class of origami and kirigami metamaterials and metastructures. Thus, the scope and contribution of this paper is much broader than it would be superficially perceived. In summary, a comprehensive review and evolving perspectives on origami‐based tubular metamaterials, as presented in this paper, would contribute to myriad possibilities to explore the exploitable nexus of crease architecture, folding mechanics and stimuli‐responsive physics for developing advanced engineering systems with improved and unprecedented functionalities.

## Conflict of Interest

The authors declare no conflict of interest.

## Supporting information

Supplemental Video 1

Supplemental Video 2

Supplemental Video 3

## Data Availability

All data sets used to generate the results are available in the main paper. Further details could be obtained from the corresponding authors upon reasonable request.
